# Supplementary material for: Structure of Full-Length SMC and Rearrangements Required for Chromosome Organization
Source: Mol Cell. 2017 Jul 20;67(2):334–347.e5. doi: 10.1016/j.molcel.2017.06.010 (PMC5526789; doi:10.1016/j.molcel.2017.06.010)
Supplement: Document S1. Figures S1–S7 and Tables S1–S3 [file mmc1.pdf]

Molecular Cell, Volume *67*

Supplemental Information

Structure of Full-Length SMC

and Rearrangements Required

for Chromosome Organization

Marie-Laure Diebold-Durand, Hansol Lee, Laura B. Ruiz Avila, Haemin Noh, Ho-Chul Shin, Haeri Im, Florian P. Bock, Frank Bürmann, Alexandre Durand, Alrun Basfeld, Sihyun Ham, Jérôme Basquin, Byung-Ha Oh, and Stephan Gruber

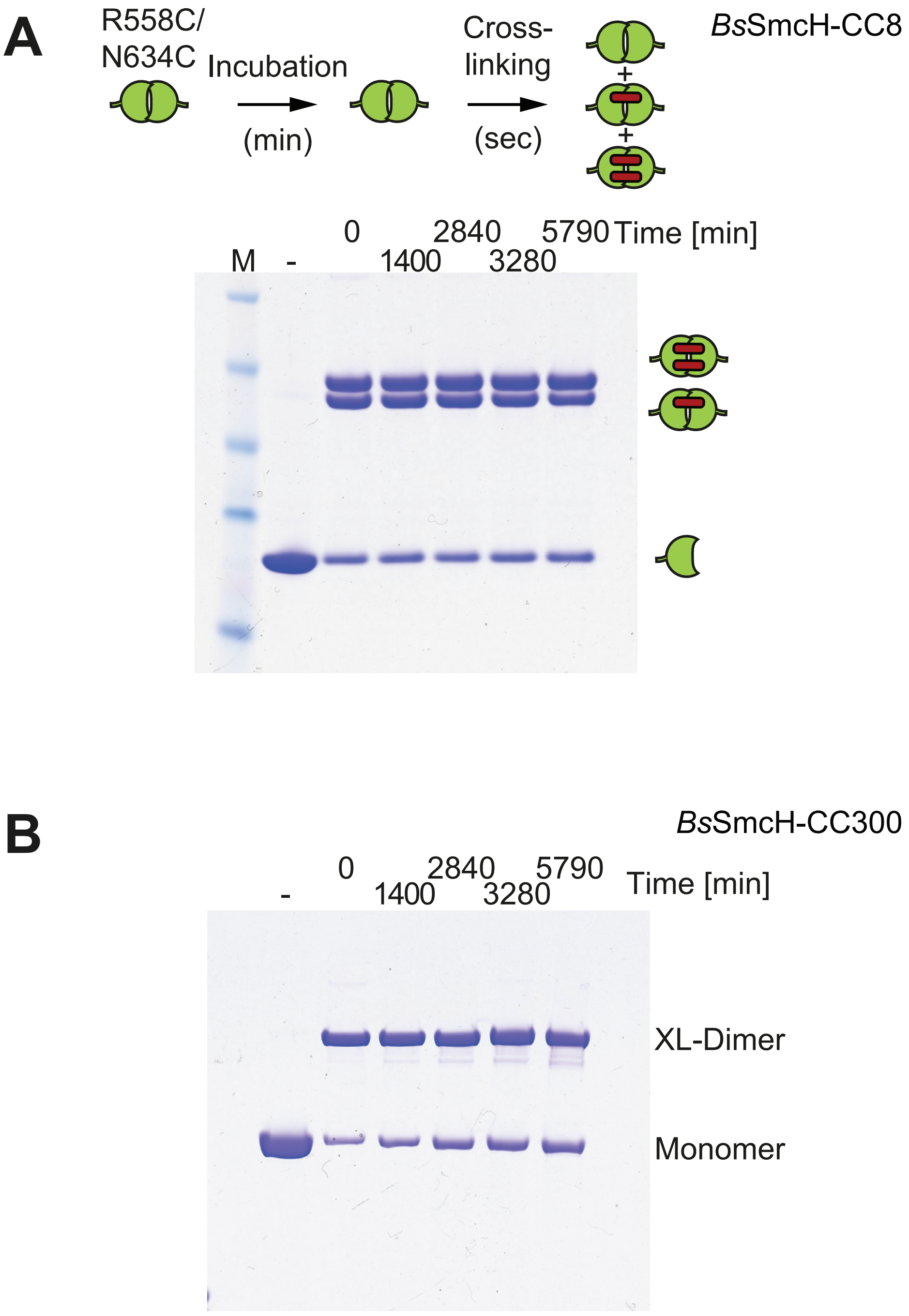
A
R558C/
N634C
Incubation
(min)
Cross-
linking
(sec)
BsSmcH-CC8
M
-
0
1400
2840
3280
5790
Time [min]
B
BsSmcH-CC300
-
0
1400
2840
3280
5790
Time [min]
XL-Dimer
Monomer

Figure S1

Figure S1. Robust long-term stability of cysteine cross-linking. Related to Figure 1.

(A) Purified preparations of *Bs*SmcH-CC8(R558C, N634C) were incubated at 37°C. At the indicated time points samples were cross-linked by BMOE. Monomer and cross-linked dimer species were separated by SDS-PAGE and detected by Coomassie staining. ´-´ denotes samples treated with DMSO instead of BMOE.

(B) Same as in (A) with *Bs*SmcH-CC300(R558C, N634C). Please note that robust cysteine cross-linking is observed after extended periods of incubation at 37°C. The lack of cross-linking observed in Figure 1C is thus due to slow subunit turn-over.

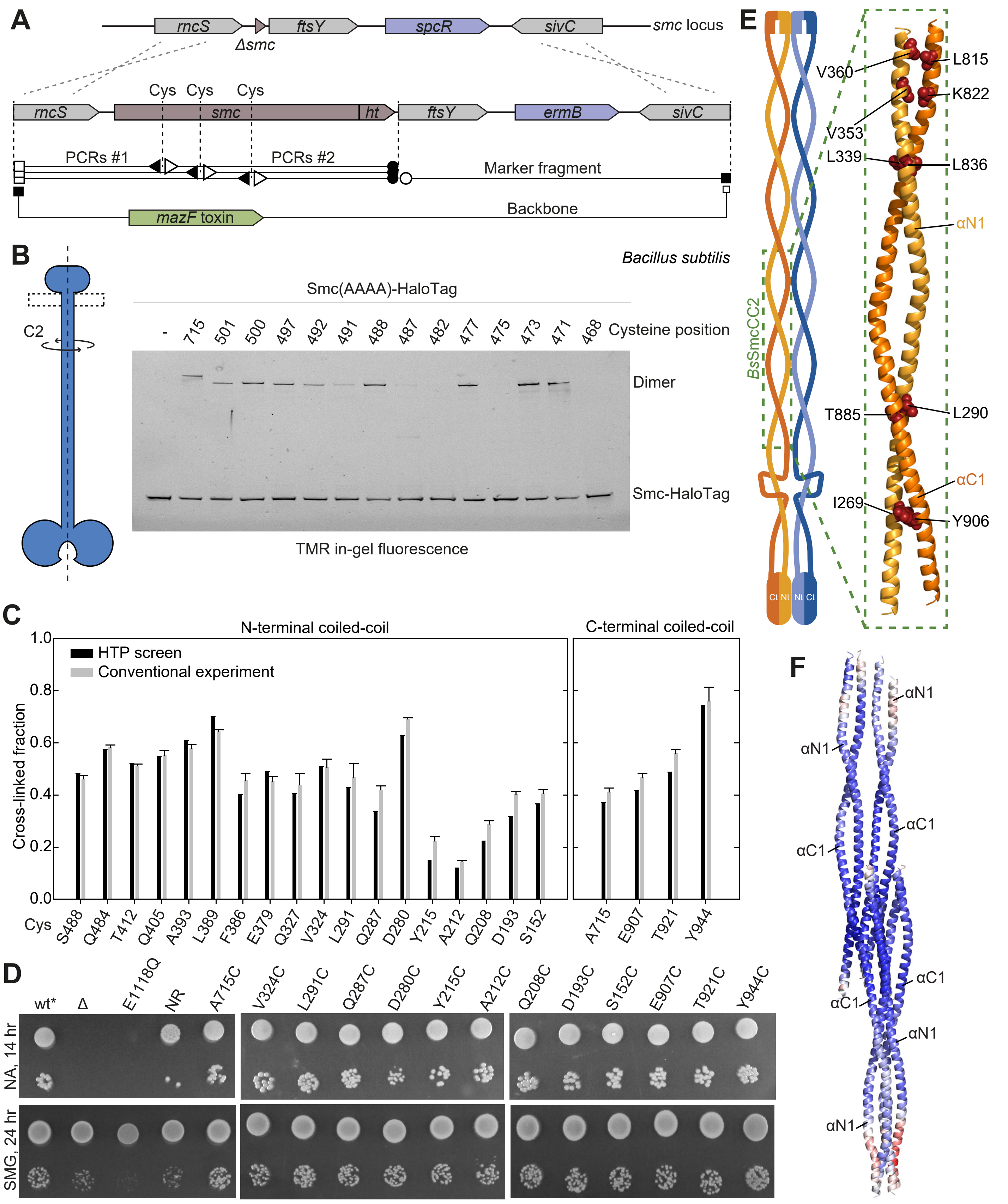
A
rncS
ftsY
spcR
sivC
smc locus
Δsmc
Cys Cys Cys
smc
ht
ermB
PCRs #1
PCRs #2
Marker fragment
mazF toxin
Backbone
B
C2
Bacillus subtilis
Smc(AAAA)-HaloTag
- 715 501 500 497 492 491 488 487 482 477 475 473 471 468
Cysteine position
Dimer
Smc-HaloTag
TMR in-gel fluorescence
C
N-terminal coiled-coil
C-terminal coiled-coil
HTP screen
Conventional experiment
Cross-linked fraction
1.0
0.8
0.6
0.4
0.2
0.0
Cys
S488 Q484 T412 Q405 A393 L389 F386 E379 Q327 V324 L291 Q287 D280 Y215 A212 Q208 D193 S152
A715 E907 T921 Y944
D
wt* Δ E1118Q NR A715C V324C L291C Q287C D280C Y215C A212C Q208C D193C S152C E907C T921C Y944C
NA, 14 hr
SMG, 24 hr
E
BsSmcCC2
Ct Nt Nt Ct
V360
L815
K822
V353
L339
L836
αN1
T885
L290
αC1
I269
Y906
F
αN1
αC1

Figure S2

Figure S2. Mapping of the Smc rod by cysteine cross-linking and structural analysis.

Related to Figure 2.

(A) Gene targeting strategy.

(B) Exemplary output of cysteine mapping screen for Smc residues located close to the Smc hinge domain in the N-terminal α-helix. Smc-HaloTag species labelled by HaloTag-TMR are separated by SDS-PAGE and detected by in-gel fluorescence scanning. Smc(A715C) serves as a positive control (Soh et al., 2015). Please note that the migration of the cross-linked dimer fraction depends on the position of the cysteine residue. All original gel images are available on Mendeley Data.

(C) Selected Smc(Cys) mutants were generated by conventional allelic replacement without selection for Smc function during growth. Cysteine cross-linking efficiency was compared to corresponding results from the high-throughput screen ('HTP'). Mean and standard deviation from three technical replicates are shown for the conventional experiment.

(D) Growth of selected Smc(Cys) mutants on nutrient rich ('Oxoid nutrient agar', 'ONA') and nutrient poor medium ('SMG'). Serial dilutions of overnight-cultures were spotted. Strain denoted as 'NR' is not related to this study.

(E) Organization of the Smc coiled coil in *Bs*SmcCC2 in cartoon representation. N- and the C-terminal helices (residues 246-379 and 793-929, respectively) are colored in bright and dark orange colors, respectively. Previously identified heptad residues (positions 'a' and 'd') are displayed as spheres in red colors (Minnen et al., 2016; Waldman et al., 2015).

(F) Organization of the two dimers of BsSmcCC2 in the crystal unit. Molecules are colored according to the local B-factor, lowest in blue, highest in red. The two dimers are organized top-to-tail in the asymmetric unit, the head proximal region of the coiled coils are juxtaposed. Probably due to crystal packing, the electron density of the head-proximal region is better defined, the main- and side-chains being clearly visible in the density.

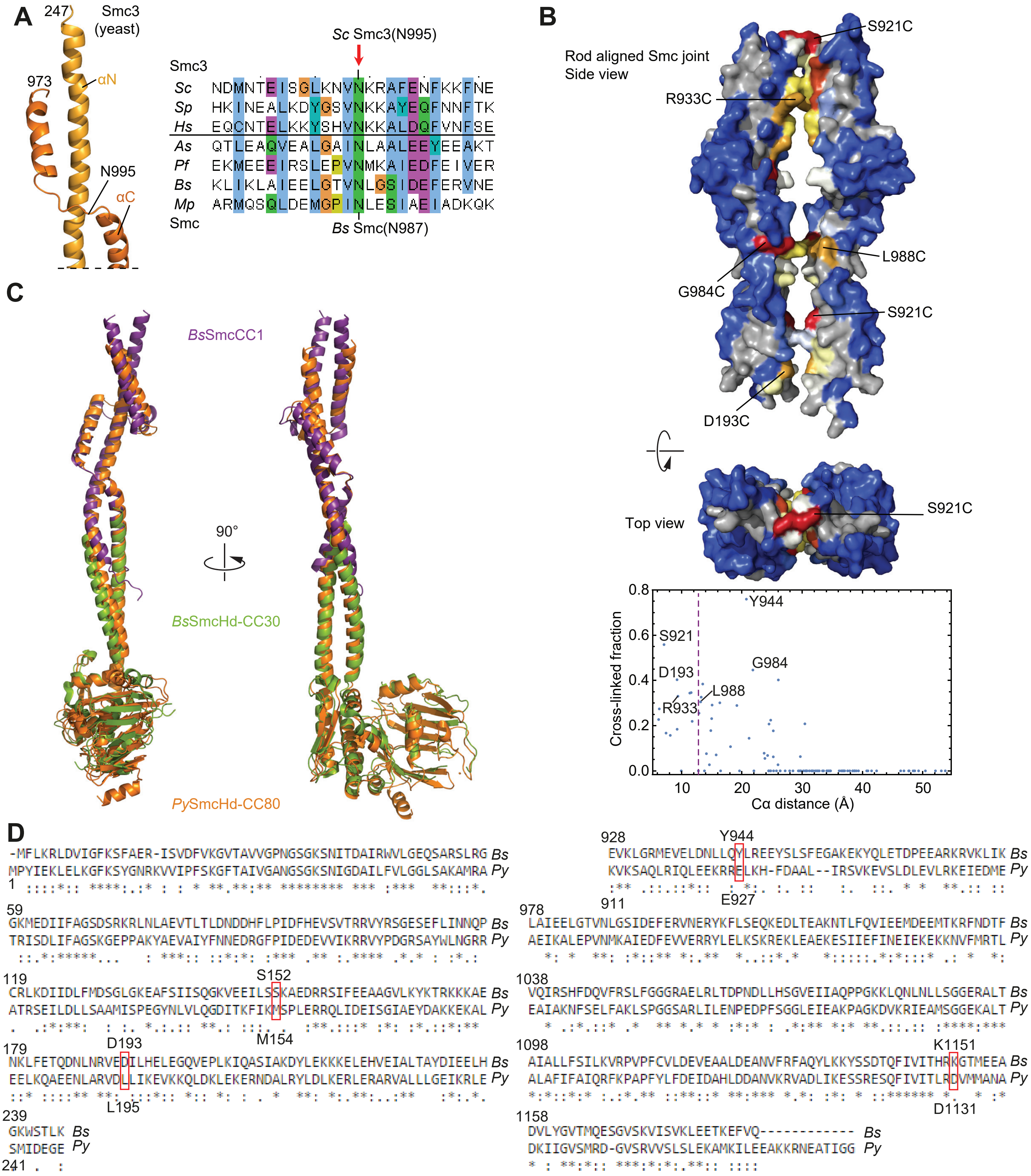
A
247
Smc3
(yeast)
973
αN
N995
αC
Sc Smc3(N995)
Smc3
Sc NDMNTEISGLKNVNKRAFENFKKFNE
Sp HKINEALKDYGSVNKKAYEQFNNFTK
Hs EQCNTELKKYSHVNKKALDQFVNFSE
As QTLEAQVEALGAINLAALEEYEEAKT
Pf EKMEEEIRSLEPVNMKAIEDFEIVER
Bs KLIKLAIEELGTVNLGSIDEFERVNE
Mp ARMQSQLDEMGPINLESIAEIADKQK
Smc
Bs Smc(N987)
B
Rod aligned Smc joint
Side view
S921C
R933C
L988C
G984C
S921C
D193C
Top view
S921C
Cross-linked fraction
Cα distance (Å)
Y944
S921
G984
D193
L988
R933
C
BsSmcCC1
90°
BsSmcHd-CC30
PySmcHd-CC80
D
-MFLKRLDVIGFKSFAER-ISVDFVKGVTAVVGPNGSGKSNITDAIRWVLGEQSARSLRG Bs
MPYIEKLELKGFKSYGNRKVVIPFSKGFTAIVGANGSGKSNIGDAILFVLGGLSAKAMRA Py
GKMEDIIFAGSDSRKRLNLAEVTLTLDNDDHFLPIDFHEVSVTRRVYRSGESEFLINNQP Bs
TRISDLIFAGSKGEPPAKYAEVAIYFNNEDRGFPIDEDEVVIKRRVYPDGRSAYWLNGRR Py
S152
CRLKDIIDLFMDSGLGKEAFSIISQGKVEEILSSKAEDRRSIFEEAAGVLKYKTRKKKAE Bs
ATRSEILDLLSAAMISPEGYNLVLQGDITKFIKMSPLERRQLIDEISGIAEYDAKKEKAL Py
M154
D193
NKLFETQDNLNRVEDILHELEGQVEPLKIQASIAKDYLEKKKELEHVEIALTAYDIEELH Bs
EELKQAEENLARVDLLIKEVKKQLDKLEKERNDALRYLDLKERLERARVALLLGEIKRLE Py
L195
GKWSTLK Bs
SMIDEGE Py
Y944
EVKLGRMEVELDNLLQYLREEYSLSFEGAKEKYQLETDPEEARKRVKLIK Bs
KVKSAQLRIQLEEKRRELKH-FDAAL--IRSVKEVSLDLEVLRKEIEDME Py
E927
LAIEELGTVNLGSIDEFERVNERYKFLSEQKEDLTEAKNTLFQVIEEMDEEMTKRFNDTF Bs
AEIKALEPVNMKAIEDFEVVERRYLELKSKREKLEAEKESIIEFINEIEKEKKNVFMRTL Py
VQIRSHFDQVFRSLFGGGRAELRLTDPNDLLHSGVEIIAQPPGKKLQNLNLLSGGERALT Bs
EAIAKNFSELFAKLSPGGSARLILENPEDPFSGGLEIEAKPAGKDVKRIEAMSGGEKALT Py
K1151
AIALLFSILKVRPVPFCVLDEVEAALDEANVFRFAQYLKKYSSDTQFIVITHRKGTMEEA Bs
ALAFIFAIQRFKPAPFYLFDEIDAHLDDANVKRVADLIKESSRESQFIVITLRDVMMANA Py
D1131
DVLYGVTMQESGVSKVISVKLEETKEFVQ------------ Bs
DKIIGVSMRD-GVSRVVSLSLEKAMKILEEAKKRNEATIGG Py

Figure S3

Figure S3. The Smc joint domain in *Bs* Smc and *Py* Smc. Related to Figure 3.

(A) Structure of part of the Smc3 joint domain present in PDB: 4UX3 (left panel). Sequence alignment of C-terminal SMC joint residues indicating highly-conserved residue including an invariable asparagine.

(B) Two monomers of *Bs*SmcCC1 (in side view and top view) were arranged side-by-side in a manner that minimizes the overall distances between cross-linkable cysteine side chains in the Smc joint domain and the adjacent coiled coil. Rendering, color coding and distance/cross-linking efficiency graph as in Figure 2B.

(C) Superimposition of *Py*SmcHd-CC80 (in orange colors) with *Bs*SmcCC1 (in purple colors) and the Smc moiety of *Bs*SmcHd-CC30:ATPγS–ScpA^C (in green colors). Two side views are displayed in the left and right panels. Overall, the structures display excellent overlap.

(D) *Bs* Smc residues corresponding to residues in *Py*SmcHd-CC80. N-terminal Smc sequences (left panel) and C-terminal Smc sequences (right panel) are displayed. Numbers above and below the sequence lines denote *Bs* and *Py* Smc positions, respectively. Selected residues are highlighted by red boxes.

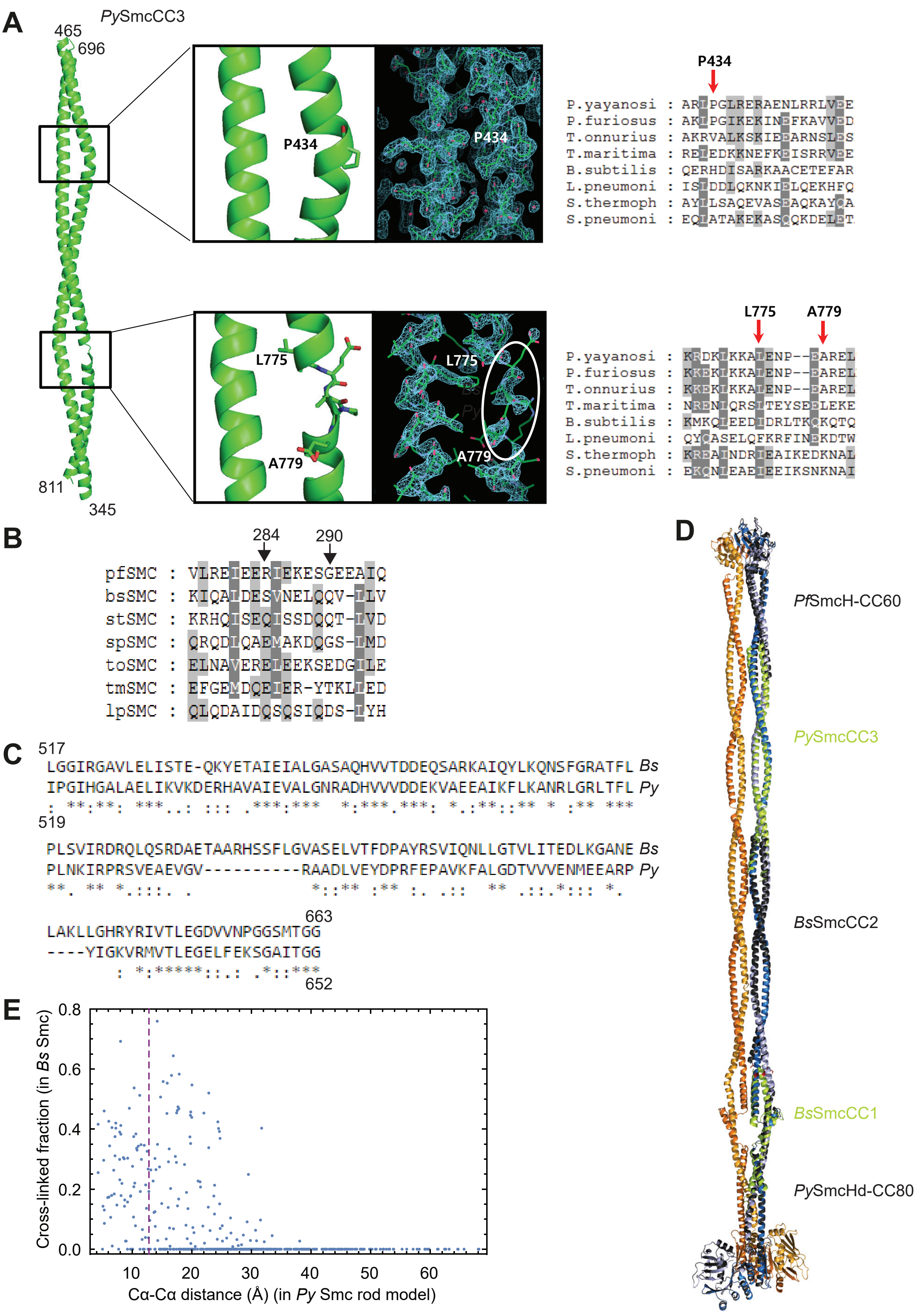

A
PySmcCC3
465
696
811
345
P434
L775
A779
P.yayanosi : ARIPGLRERAENLRRLVEE
P.furiosus : AKLPGIKEKINEFKAVVED
T.onnurius : AKRVALKSKIEEARNSLES
T.maritima : RELEDKKNEFKEISRRVEE
B.subtilis : QERHDISARKAACETEFAR
L.pneumoni : ISIDDLQKNKIELQEKHFQ
S.thermoph : AYLLSAQEVASEAQKAYCA
S.pneumoni : EQLATAKEKASQQKDELET
P.yayanosi : KRDKIKKALENP--EAREL
P.furiosus : KKEKLKKALENP--EAREL
T.onnurius : KKEKLKKALENP--EAREL
T.maritima : NRENLQRSLTEYSEELEKE
B.subtilis : KMKQIEEDIDRLTKQKQTQ
L.pneumoni : QYQASELQFKRFINEKDTW
S.thermoph : KREAINDRIEAIKEDKNAL
S.pneumoni : EKQNIEAEIEEIKSNKNAI
B
284
290
pfSMC : VLREIEERIEKESGEEAIQ
bsSMC : KIQAIDESVNELQQV-ILV
stSMC : KRHQISEQISSDQQT-IVD
spSMC : QRQDIQAEMAKDQGS-IMD
toSMC : ELNAVEREIEEKSEDGILE
tmSMC : EFGEMDQEIER-YTKLIED
lpSMC : QLQDAIDQSQSIQDS-IYH
C
517
LGGIRGAVLELISTE-QKYETAIEIALGASAQHVVTDDEQSARKAIQYLKQNSFGRATFL Bs
IPGIHGALAELIKVKDERHAVAIEVALGNRADHVVVDDEKVAEEAIKFLKANRLGRLTFL Py
519
PLSVIRDRQLQSRDAETAARHSSFLGVASELVTFDPAYRSVIQNLLGTVLITEDLKGANE Bs
PLNKIRPRSVEAEVGV----------RAADLVEYDPRFEPAVKFALGDTVVVENMEEARP Py
663
LAKLLGHRYRIVTLEGDVVNPGGSMTGG
----YIGKVRMVTLEGELFEKSGAITGG
652
D
PfSmcH-CC60
PySmcCC3
BsSmcCC2
BsSmcCC1
PySmcHd-CC80
E
Cross-linked fraction (in Bs Smc)
0.0
0.2
0.4
0.6
0.8
10
20
30
40
50
60
Cα-Cα distance (Å) (in Py Smc rod model)

Figure S4

Figure S4. Reconstruction and evaluation of the Smc rod structure. Related to Figure 4.

(A) Structural overview (left panel) of *Py*SmcCC3. Regions displaying deviations from the canonical coiled coil architecture are shown in boxed insets (middle panels). Residues P434 and G435 result in the kinking of the N-terminal α–helix (top panel), while two residues lacking in the C-terminal helix cause a deformation of the α–helical pattern. Sequence alignments are shown for these selected regions (right panels).

(B) A single amino-acid insertion in the N-terminal *Pf* Smc coiled coil. Sequences were aligned taking into account the predicted coiled coil register.

(C) *Bs* Smc hinge domain residues corresponding to *Py* Smc residues. Numbers above and below the sequence lines denote *Bs* and *Py* Smc residues, respectively.

(D) Superimposition of the Smc rod model with individual crystal structures. Five structures were superimposed with the blue monomer of the Smc rod model shown in Figure 4B. The top and bottom ends of crystal structures were slightly adjusted manually to connect them into a straight molecule.

(E) Plot of Cα-Cα distance against cross-linking efficiency for the *Py* Smc rod model shown in Figure 4C. The data is available in Table S5.

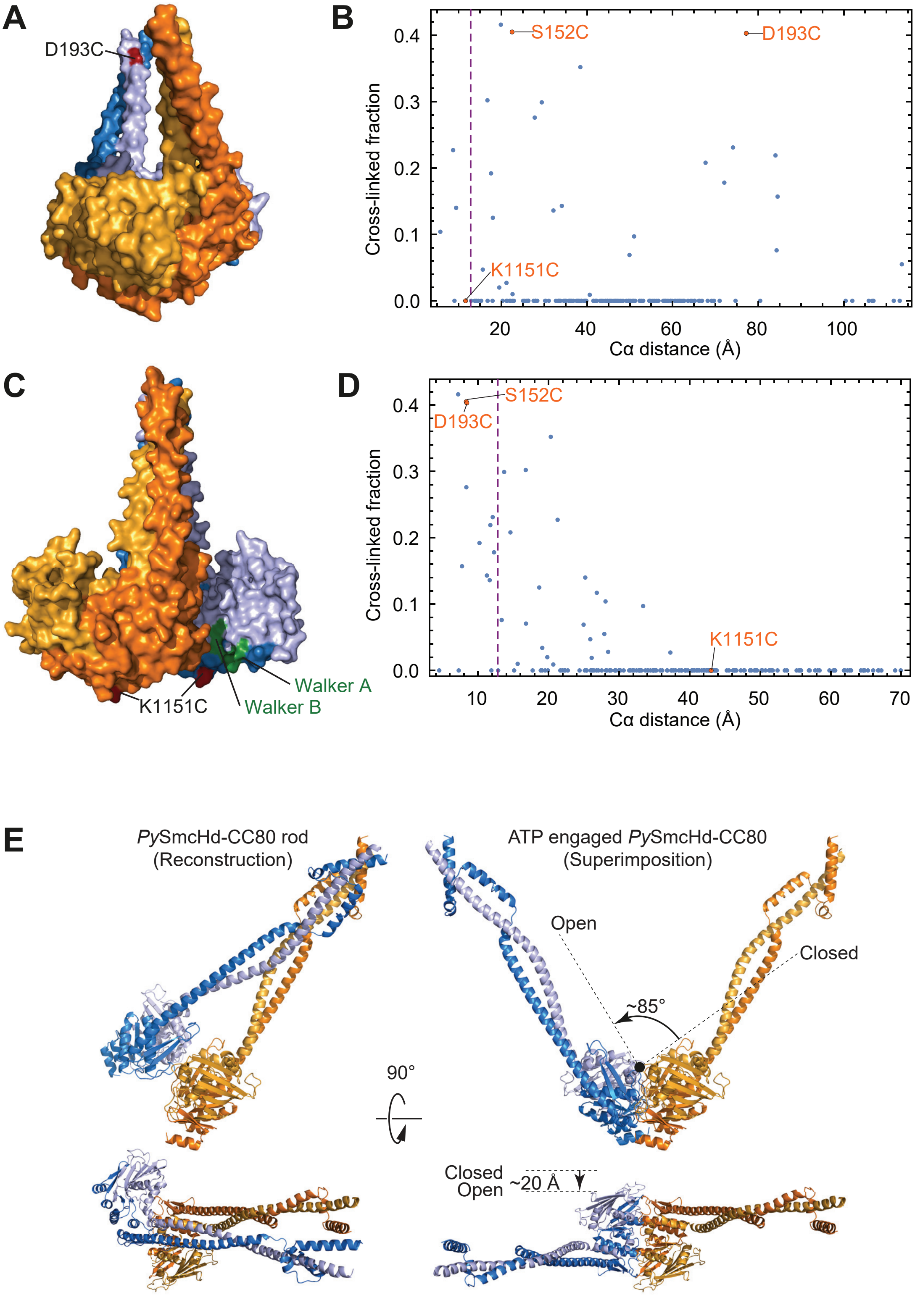
A
D193C
B
S152C
D193C
K1151C
Cross-linked fraction
Cα distance (Å)
C
K1151C
Walker A
Walker B
D
S152C
D193C
K1151C
Cross-linked fraction
Cα distance (Å)
E
PySmcHd-CC80 rod
(Reconstruction)
ATP engaged PySmcHd-CC80
(Superimposition)
Open
Closed
~85°
90°
Closed
Open
~20 Å

Figure S5

Figure S5. Structural comparison of rod-aligned and ATP-engaged Smc heads. Related to Figure 5.

(A-D) Characterization of engaged (A, B) and juxtaposed (C, D) Smc heads. Side views of models shown in Figure 5B and 5C (A, C, respectively). Plotting Cα-Cα distance distribution against cross-linking efficiencies shown in Figure 2A (B, D).

(E) Comparison of rod-aligned (left panels; taken from Figure 4B) and ATP-engaged *Py*SmcHd-CC80 (right panels; superimposed onto PDB: 1XEX) in side (top panels) and top views (bottom panels). The position and orientation of the monomer in orange colors in identical in the two top and the two bottom panels. The monomer in blue colors tilts by about 85° relative to the other monomer (top panel) and translates by about 20 Å.

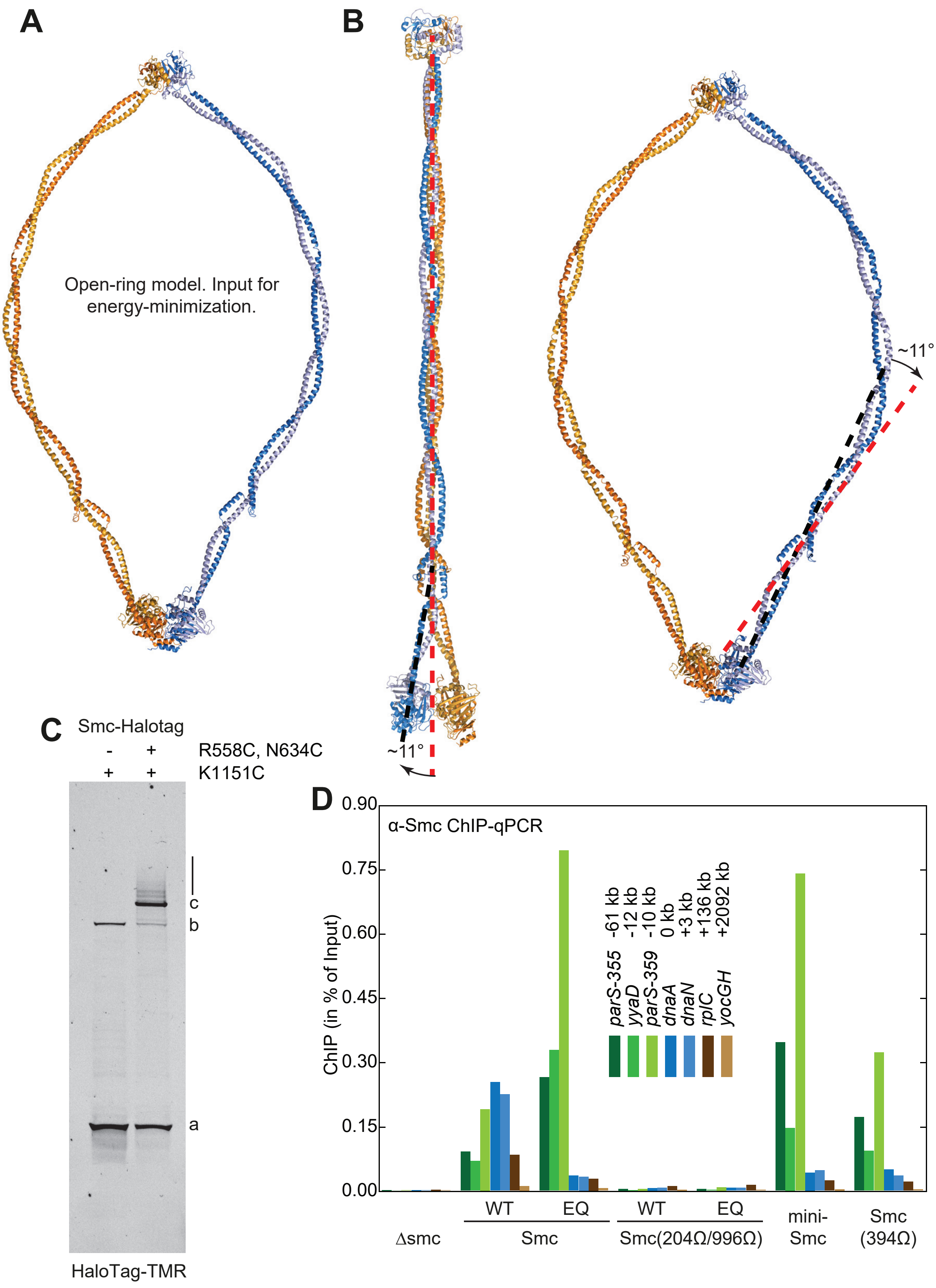
A
B
Open-ring model. Input for energy-minimization.
~11°
~11°
C
Smc-Halotag
- + R558C, N634C
+ + K1151C
c
b
a
HaloTag-TMR
D
α-Smc ChIP-qPCR
ChIP (in % of Input)
0.90
0.75
0.60
0.45
0.30
0.15
0.00
parS-355 -61 kb
yyaD -12 kb
parS-359 -10 kb
dnaA 0 kb
dnaN +3 kb
rplC +136 kb
yocGH +2092 kb
Δsmc
WT
EQ
Smc
WT
EQ
Smc(204Ω/996Ω)
mini-Smc
Smc (394Ω)

Figure S6

Figure S6. Model of an open Smc dimer. Related to Figure 6.

(A) Input model for energy-minimization constructed by bending of the coiled coil from the Smc hinge to the Smc joint.

(B) The Smc joint – as observed in the model of the Smc rod (left panel) – bends the coiled coil away from the symmetry axis possibly to support wide opening of the Smc ring upon head engagement (right panel – energy minimized model).

(C) Simultaneous engagement of the Smc hinge and ATP dimer Smc heads. Similar to the experiment shown in Figure 6B using Smc proteins with wild-type ATPase domain. Please note that the contrast is enhanced to display low-abundance species.

(D) Chromosome localization of Smc proteins with modified coiled coils. Similar to the experiment shown in Figure 6F using Smc proteins lacking a HaloTag and cysteine modifications.

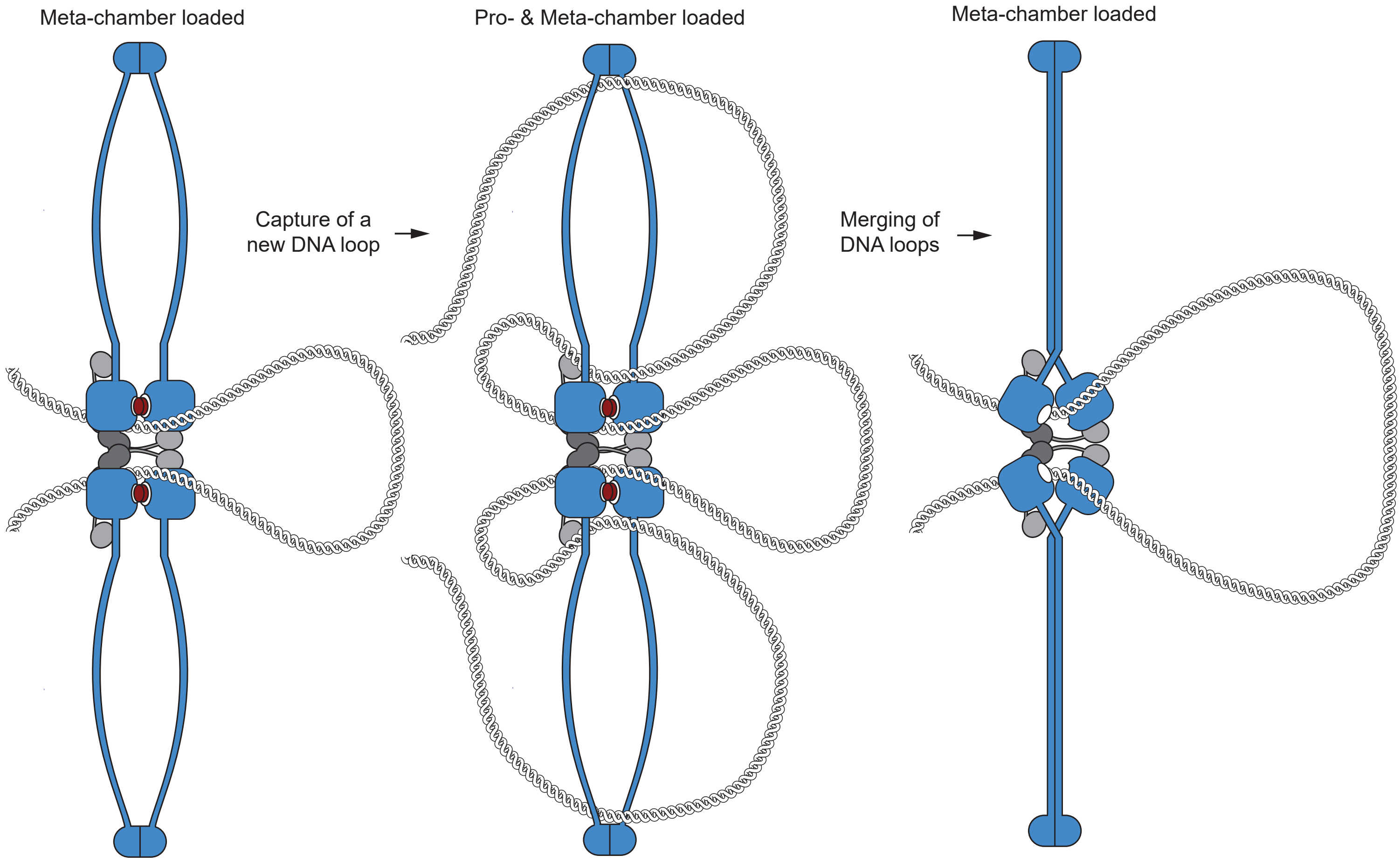
Meta-chamber loaded
Capture of a
new DNA loop
Pro- & Meta-chamber loaded
Merging of
DNA loops
Meta-chamber loaded

Figure S7

Figure S7. A tentative SMC handcuff model. Related to Figure 7.

As in Figure 7, involving a putative ‘handcuff’ Smc-ScpAB dimer. The two Smc-ScpAB complexes translocate in opposite orientation on the DNA, each being topologically loaded onto a single DNA double helix.

Table S1, related to Figure 2 and 3. Data collection and structure refinement statistics.

**Data Collection**	***Bs*SmcCC2 SeMet**	***Bs*SmcCC1 SeMet**	***Bs*SmcCC1 native**
X-ray source	SLS PXII XD10SA[a]	DESY BL P11[b]	DESY BL P11[b]
Space group	P1	$P2_12_12$	$P2_12_12$
a, b, c (Å)	44.30, 42.73, 174.60	64.88, 77.32, 83.98	64.83, 78.03, 83.86
α, β, γ (°)	89.97, 93.64, 89.97	90, 90, 90	90, 90, 90
Wavelength (Å)	0.9792	0.9793	0.9806
Resolution (Å)	87.12 - 2.892	50.0-2.38	50.0-1.8
R_{sym} (%)[d]	8.3 (61)	5.8 (84.4)	4.0 (76.0)
$I/\sigma(I)$	12.33 (2.58)	20.45 (2.03)	16.95 (1.75)
Completeness (%)	98.54 (96.20)	99.8 (99)	99.7 (99.2)
Redundancy	3.6 (3.6)	6.9 (6.9)	4.3 (4.2)
Refinement			
Resolution (Å)	50.00 - 3.295		50.00 - 1.898
No. of reflections	37323		65454
R_{work} / R_{free} (%)	27.27 / 29.73		18.27 / 21.35
bond lengths (Å) / angles (º)	0.003 / 0.84		0.011 / 1.035
Average B-values (Å^2)	109.6		
Favored	98.29		98.63
allowed	1.49		1.03
Disallowed	0.21		0.34

[a]Villigen, Switzerland, [b]Hamburg Germany

[d]The numbers in parentheses are the statistics from the highest resolution shell.

Table S1, related to Figure 3, 4 and 5. Continued.

Data Collection	**PySmcHd-CC80**	***Bs*SmcHd-CC30: ATPγS–ScpA[c]**	***Py*SmcCC3**
X-ray source	BL5C, PAL[a]	BL5A, PF[b]	BL5C, PAL[a]
Space group	C2	$P2_12_12_1$	$P2_1$
a, b, c (Å)	120.32, 50.84, 122.06	88.10, 104.78, 185.22	36.18, 53.71, 71.40
α, β, γ (°)	90, 118.09, 90	90, 90, 90	90, 90.83, 90
Wavelength (Å)	0.97941	1.0000	0.97941
Resolution (Å)	50.0-2.6	50.0-3.5	50.0-2.0
R_{sym} (%)[d]	7.9 (32.4)	5.6 (27.9)	5.4 (23.5)
$I/\sigma(I)$	13.7 (2.6)	12.3 (3.7)	23.8 (2.1)
Completeness (%)	92.8 (69.1)	92.6 (86.5)	94.4 (78.5)
Redundancy	4.2 (2.0)	5.4 (1.8)	4.3 (2.5)
Refinement			
Resolution (Å)	50.0-2.6	50.0-3.5	50.0-2.0
No. of reflections	18871	35074	17586
R_{work} / R_{free} (%)	23.1 / 28.6	25.2 / 30.1	22.8 / 24.7
bond lengths (Å) / angles (°)	0.006 / 0.878	0.009 / 1.256	0.006 / 0.975
Average B-values (Å^2)	69.6	57.51	49.6
Most favored/Favored	89.6 / 9.0	87.6 / 10.6	97.7 / 2.3
Generously allowed	1.1	1.3	0.0
Disallowed	0.4	0.4	0.0

[a]Pohang Accelerator Laboratory, [b]Photon Factory

[d]The numbers in parentheses are the statistics from the highest resolution shell.

Table S2, related to Figures 2, 5 and 6. Strain usage.

Figure	Strains
5D	BSG2264, BSG2300, BSG1457, BSG2588, BSG2608, BSG1607, BSG2589, BSG2606, BSG1600, BSG2265, BSG2313, BSG1488, BSG2314, BSG2315, BSG1598
6B	BSG1488, BSG1640, BSG1666
6C	BSG1457, BSG1007, BSG2694
6D	BSG1457, BSG1488, BSG2694, BSG2693, BSG2408, BSG2135
6E	BSG1921, BSG1457, BSG1922, BSG1488, BSG2680, BSG2694, BSG2692, BSG2693
6F	BSG1007, BSG1457, BSG1488, BSG2694, BSG2693, BSG2408, BSG2135
S2C	BSG2326, BSG2324, BSG2382, BSG2381, BSG2270, BSG2322, BSG2063, BSG2320, BSG2413, BSG2299, BSG2379, BSG2378, BSG2267, BSG2377, BSG2376, BSG2375, BSG2300, BSG2264, BSG1921, BSG2383, BSG2385, BSG2386
S2D	BSG1360, BSG1007, BSG1008, BSG1921, BSG2299, BSG2379, BSG2378, BSG2267, BSG2377, BSG2376, BSG2375, BSG2300, BSG2264, BSG2383, BSG2385, BSG2386
S6C	BSG1638, BSG2638
S6D	BSG1007, BSG1002, BSG1008, BSG2648, BSG2688, BSG2091, BSG2018

Table S3, related to STAR methods. Primers used for qPCR

**Locus**	**Primer 1**	**Primer 2**
parS-355	taattcatcatcgcgctcaa	aatgccgattacgagtttgc
yyaD	cttgcgatttttgcttctcc	acatcaccatacgtggacga
parS-359	aaaaagtgattgcggagcag	agaaccgcatctttcacagg
dnaA	gatcaatcggggaaagtgtg	gtagggcctgtggatttgtg
dnaN	gaattccttcaggccattga	gatttctggcgaattggaag
rplC	ttgacgacaagcgtgaaaag	ttcatacgcatccatttcca
yocGH	tccatatcctcgctcctacg	attctgctgatgtgcaatgg
