# Supplementary figures and images for: Structure of Full-Length SMC and Rearrangements Required for Chromosome Organization
Source: Mol Cell. 2017 Jul 20;67(2):334–347.e5. doi: 10.1016/j.molcel.2017.06.010 (PMC5526789; doi:10.1016/j.molcel.2017.06.010)

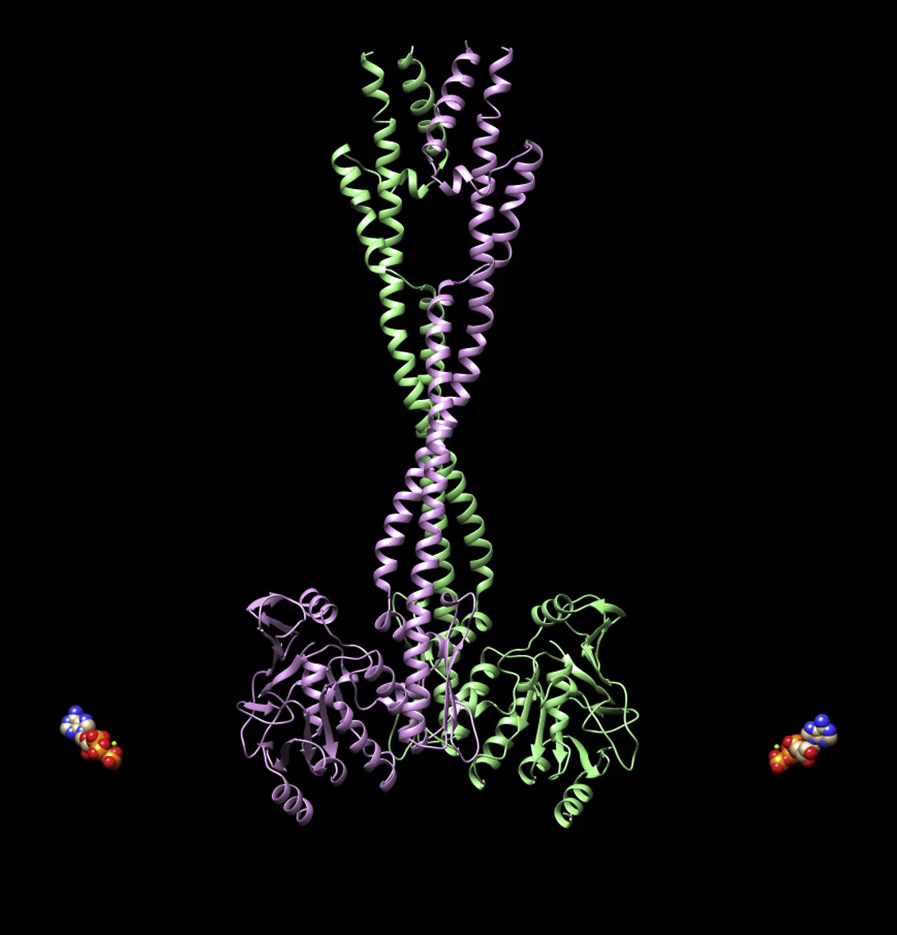

Supplement: Movie 1, Related to Figure 5. Morph Movie of the Rod-to-Ring Transition—Side View [file mmc7.jpg]

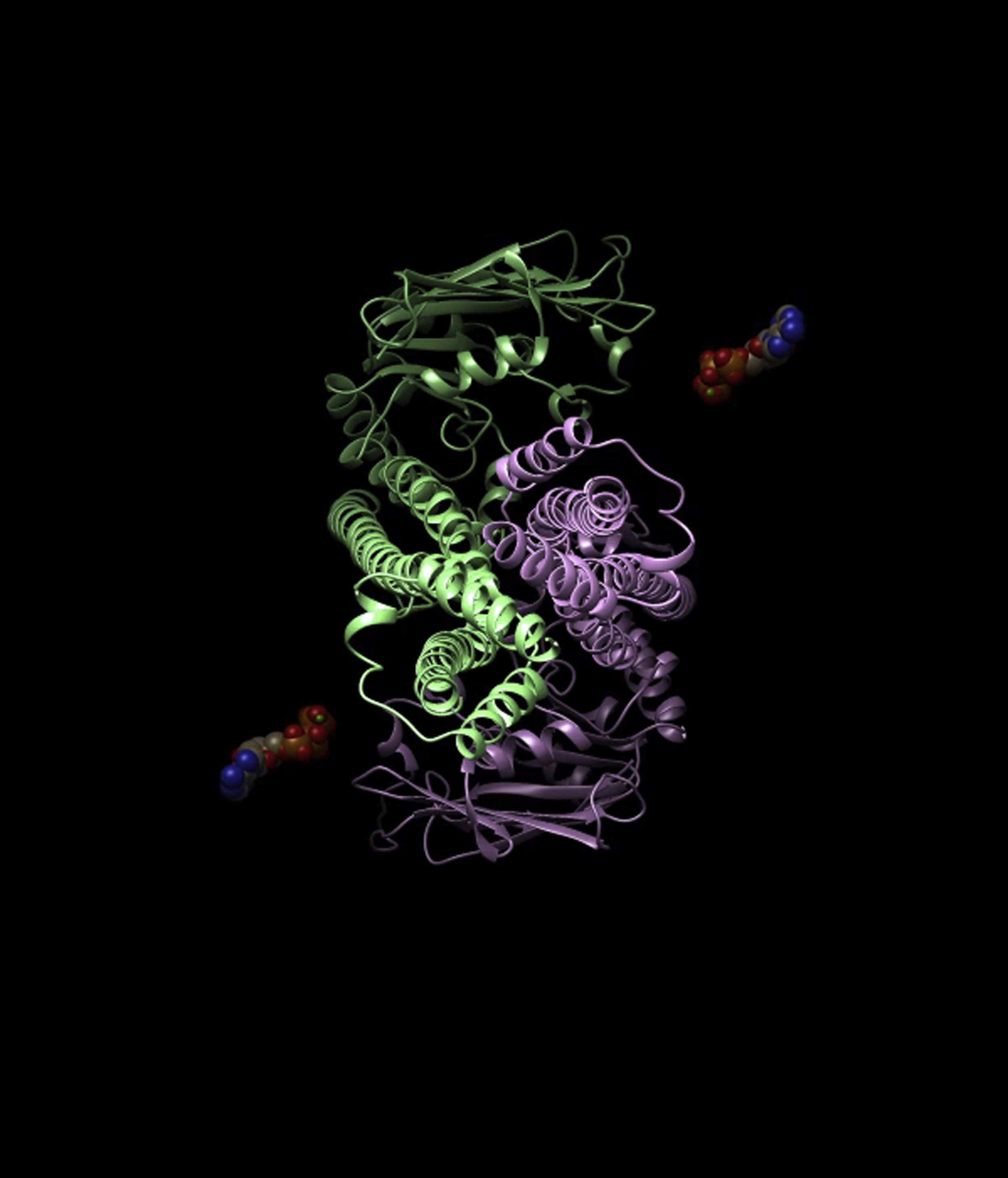

Supplement: Movie 2, Related to Figure 5. Morph Movie of the Rod-to-Ring Transition—Top View [file mmc8.jpg]
