# Supplementary material for: Structure of Full-Length SMC and Rearrangements Required for Chromosome Organization
Source: Mol Cell. 2017 Jul 20;67(2):334–347.e5. doi: 10.1016/j.molcel.2017.06.010 (PMC5526789; doi:10.1016/j.molcel.2017.06.010)
Supplement: Document S2. Article plus Supplemental Information [file mmc9.pdf]

Molecular Cell

Article

Structure of Full-Length SMC and Rearrangements Required for Chromosome Organization

Graphical Abstract

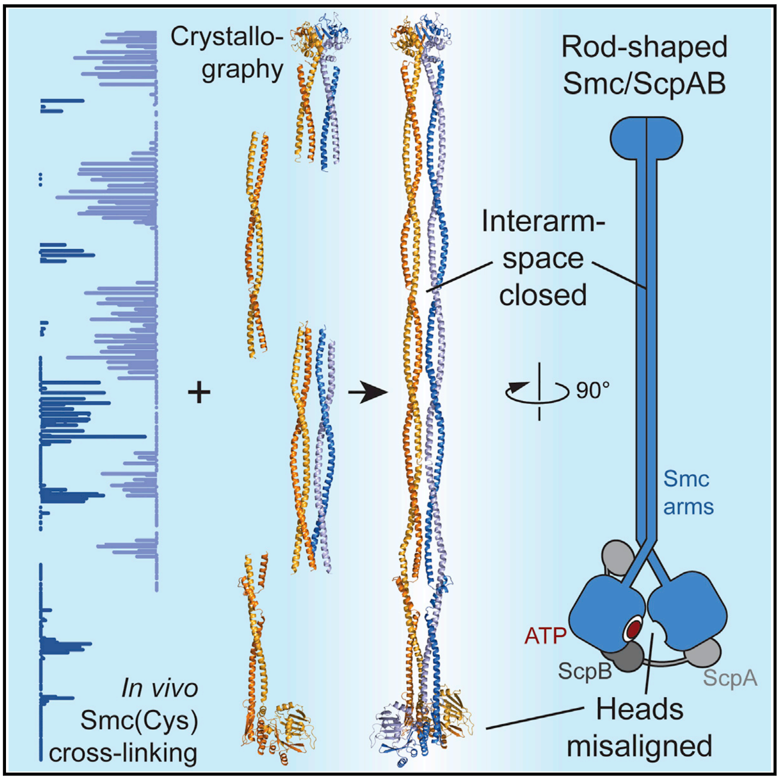

Highlights

- Crystallography and in vivo cross-linking reveal the architecture of prokaryotic Smc
- Juxtaposition of the Smc arms misaligns the two Smc ATPase domains
- Smc head engagement mechanically opens an interarm space
- A model for DNA loop extrusion driven by the SMC ATPase cycle is presented

Authors

Marie-Laure Diebold-Durand, Hansol Lee, Laura B. Ruiz Avila, ..., Jérôme Basquin, Byung-Ha Oh, Stephan Gruber

Correspondence

bhoh@kaist.ac.kr (B.-H.O.), stephan.gruber@unil.ch (S.G.)

In Brief

By combining high-throughput in vivo cysteine cross-linking and crystallography, Diebold-Durand et al. construct a high-resolution model of full-length prokaryotic Smc. It reveals that the rod-shaped Smc dimer lacks chambers for DNA and features misaligned head domains. Smc head engagement mechanically opens an interarm space.

Diebold-Durand et al., 2017, Molecular Cell *67*, 334–347
July 20, 2017 © 2017 The Authors. Published by Elsevier Inc.
http://dx.doi.org/10.1016/j.molcel.2017.06.010

CellPress

Molecular Cell
Article

Structure of Full-Length SMC and Rearrangements Required for Chromosome Organization

Marie-Laure Diebold-Durand,[1,3,8] Hansol Lee,[2,8] Laura B. Ruiz Avila,[1,8] Haemin Noh,[2] Ho-Chul Shin,[2,6] Haeri Im,[4] Florian P. Bock,[1,3] Frank Bürmann,[1,7] Alexandre Durand,[1,3] Alrun Basfeld,[1] Sihyun Ham,[4] Jérôme Basquin,[5] Byung-Ha Oh,[2,*] and Stephan Gruber[1,3,9,*]

[1]Chromosome Organisation and Dynamics, Max Planck Institute of Biochemistry, Am Klopferspitz 18, 82152 Martinsried, Germany
[2]Department of Biological Sciences, KAIST Institute for the Biocentury, Cancer Metastasis Control Center, Korea Advanced Institute of Science and Technology, Daejeon 305-701, Korea
[3]Department of Fundamental Microbiology, University of Lausanne, Bâtiment Biophore, 1015 Lausanne, Switzerland
[4]Department of Chemistry, Sookmyung Women's University, Cheongpa-ro-47-gil 100, Yongsan-ku, Seoul 04310, Korea
[5]Department of Structural Cell Biology, Max Planck Institute of Biochemistry, Am Klopferspitz 18, 82152 Martinsried, Germany
[6]Present address: Disease Target Structure Research Center, Korea Research Institute of Bioscience and Biotechnology, Daejeon 305-806, Korea
[7]Present address: Structural Studies, MRC Laboratory of Molecular Biology, Francis Crick Ave, Cambridge CB2 0QH, UK
[8]These authors contributed equally to this work and are listed in alphabetical order
[9]Lead Contact
*Correspondence: bhoh@kaist.ac.kr (B.-H.O.), stephan.gruber@unil.ch (S.G.)
http://dx.doi.org/10.1016/j.molcel.2017.06.010

SUMMARY

Multi-subunit SMC complexes control chromosome superstructure and promote chromosome disjunction, conceivably by actively translocating along DNA double helices. SMC subunits comprise an ABC ATPase "head" and a "hinge" dimerization domain connected by a 49 nm coiled-coil "arm." The heads undergo ATP-dependent engagement and disengagement to drive SMC action on the chromosome. Here, we elucidate the architecture of prokaryotic Smc dimers by high-throughput cysteine cross-linking and crystallography. Co-alignment of the Smc arms tightly closes the interarm space and misaligns the Smc head domains at the end of the rod by close apposition of their ABC signature motifs. Sandwiching of ATP molecules between Smc heads requires them to substantially tilt and translate relative to each other, thereby opening up the Smc arms. We show that this mechanochemical gating reaction regulates chromosome targeting and propose a mechanism for DNA translocation based on the merging of DNA loops upon closure of Smc arms.

INTRODUCTION

Proper expression of genetic information, as well as the faithful duplication and segregation of genomes during cell division, relies on a number of SMC proteins in eukaryotes (Hirano, 2016; Jeppsson et al., 2014; Merkenschlager and Nora, 2016; Peters and Nishiyama, 2012). SMC dimers assemble into SMC-kleisin complexes with a characteristic ring topology, which are thought to co-entrap chromosomal DNA double helices (Gligoris et al., 2014). Each complex, however, fulfills a unique set of functions, such as sister chromatid cohesion and the regulation of gene expression (Smc1/3 cohesin), chromosome condensation (Smc2/4 condensin), and the repair and disjunction of sister DNA molecules (Smc5/6). To do so, they must capture distinct combinations of genomic DNA segments during or after chromosomal loading. Elucidating the fundamental basis and the specific features of the loading processes is thus pivotal for our understanding of chromosome biology. In many prokaryotes—including those with an artificially minimized genome—normal growth and survival depends on a single SMC complex (Hutchison et al., 2016). In *Bacillus subtilis* (*Bs*), the Smc-ScpAB complex loads onto the chromosome near the single origin of replication and then moves toward the replication terminus in a manner that concomitantly aligns the two chromosome arms (Gruber and Errington, 2009; Minnen et al., 2016; Sullivan et al., 2009; Wang et al., 2017). Thereby, it gives the bacterial chromosome a distinct shape and promotes the timely individualization of nascent sister chromosomes (Gruber et al., 2014; Marbouty et al., 2015; Wang et al., 2014, 2015). The underlying molecular mechanism, however, remains poorly understood.

The enzymatic core of SMC complexes belongs to the family of ATP-binding cassette (ABC) ATPases, all comprising a pair of nucleotide-binding domains (NBDs), called "head" domains in SMC proteins (Hirano et al., 2001). The two NBDs engage upon ATP binding and disengage during ATP hydrolysis to regulate and drive biological processes (Hopfner, 2016; Lammens et al., 2004). In ABC transporters, engaged and disengaged NBDs stabilize alternative conformations of the associated transmembrane domain, thus allowing access to a substrate-binding pocket first from one side of the membrane and then from the other (Locher, 2016). How the NBDs of SMC complexes

 © 2017 The Authors. Published by Elsevier Inc.
This is an open access article under the CC BY license (http://creativecommons.org/licenses/by/4.0/).

drive chromosome organization is largely unclear. In the case of SMC proteins, the NBD head connects via an intramolecular coiled coil to a globular hinge dimerization domain, giving rise to SMC homodimers in prokaryotes and SMC heterodimers in eukaryotes (Haering et al., 2002). The binding of the head domains to opposite ends of a kleisin subunit creates closed tripartite protein complexes for the entrapment of DNA (Bürmann et al., 2013; Gligoris et al., 2014; Wilhelm et al., 2015).

The SMC ATPase regulates chromosomal localization of SMC-kleisin rings (Arumugam et al., 2003; Minnen et al., 2016; Weitzer et al., 2003). In *B. subtilis*, a ParB/*parS* nucleoprotein complex recruits Smc toward the replication origin (Gruber and Errington, 2009; Sullivan et al., 2009). Targeting to *parS* requires ATP binding to Smc, as well as Smc head engagement, while ATP hydrolysis is needed for the subsequent release of Smc from *parS* loading sites and its translocation onto flanking DNA in chromatin immunoprecipitation experiments (Minnen et al., 2016). While Smc head engagement is crucial for chromosomal targeting, heads are kept mostly disengaged through Smc dimerization at the hinge implying a long-distance communication between hinge and heads (Hirano and Hirano, 2002; Minnen et al., 2016). The Smc coiled coils undergo conformational changes at least near the Smc hinge domain upon DNA and ATP binding (Minnen et al., 2016; Soh et al., 2015). The coiled coils are thus excellent candidates to mediate the head/hinge communication to define SMC activity on the chromosome. Whether SMC coiled coils are sufficiently rigid to allow for mechanical communication between head and hinge domains, however, is disputed (Bürmann et al., 2017; Eeftens et al., 2016).

A high-resolution structure of ATP-engaged SMC heads has been solved several years ago, revealing striking similarities to the corresponding parts of ABC transporters (Lammens et al., 2004). While distinct architectures of open NBD conformations have been reported for several ABC transporters (Locher, 2016), these conformations have remained elusive for SMC complexes. Here, we show by crystallography and cross-linking that the prokaryotic Smc coiled coils are not flexible tethers of hinge and heads. They are rigidly anchored onto each other to form a straight rod-like state of the Smc-ScpAB complex. The Smc rod—with the help of a characteristic nonhelical region in the Smc arm—brings together the two head domains. By doing so, the Smc rod prevents the formation of integral ATP-binding pockets by the opposing heads. ATP closure of Smc heads requires wide opening of the Smc coiled coils at the heads, thus strictly coupling Smc head engagement to the opening of the interarm space. Our findings put forward a mechanochemical principle, which can explain active DNA loop extrusion based on recurrent ATP driven reorganization of the Smc complex.

RESULTS

The Smc Coiled Coil Stabilizes the Smc Hinge Dimer

Crystal structures and cross-linking of hinge-proximal SMC coiled coils, as well as electron micrographs, implied that prokaryotic Smc and eukaryotic condensin display a highly defined rod-like architecture (Anderson et al., 2002; Soh et al., 2015). However, this proposition was recently challenged by AFM imaging, suggesting that yeast condensin Smc2/4 might harbor unusually flexible coiled coils (Eeftens et al., 2016). To test whether the Smc coiled coil stabilizes bacterial Smc dimers—as expected for rod-shaped dimers but not for dimers with flexible arms—we measured the exchange of subunits in Smc dimers in vitro. We mixed equal amounts of two purified Smc hinge domains, designated as *Bs*SmcH-CC8, harboring R558C and N634C, respectively (Soh et al., 2015). At selected time intervals, we determined the presence of Smc heterodimers by chemical cross-linking of R558C to N634C at the hinge dimer interface. Based on the accumulation of cross-linked dimer over time, we estimated a half-life of about 49 min for the isolated Smc hinge dimer at 37°C (Figures 1A and 1B). In contrast, little, if any, turnover of subunits was detected even after extended periods of incubation when a Smc hinge domain with long coiled coil, called *Bs*SmcH-CC300, was used (Figures 1B and 1C). Long Smc arms thus prevent the dissociation of the hinge dimer, possibly by directly associating with one another in a large majority of Smc dimers at any given moment.

Mapping the Smc Rod by Cysteine Scanning

To delineate the architecture of Smc coiled coils in the Smc dimer, we next performed an extensive cysteine cross-linking screen in vivo. Predicated on a symmetric nature of the putative bacterial Smc rod (Figure 2A), we aimed to identify residues located at the axis of the rod-shaped dimer. These ''axial'' residues are, per se, positioned in close vicinity of their symmetry mates in the Smc homodimer and should thus allow chemical cross-linking when mutated to cysteine. To do so, we systematically substituted individual amino acids by cysteine and performed thiol-specific cross-linking in intact *B. subtilis* cells. To generate a collection of Smc(Cys) mutants, we targeted the endogenous *smc* locus—harboring an in-frame *smc* deletion—using Halo-tagged *Smc* constructs assembled from Golden Gate fragments (Figure S2A). We selected functional *smc(cys)* alleles by growing transformants on nutrient-rich medium (Gruber et al., 2014) and cross-linked cells using the cysteine-reactive compound bis(maleimido)ethane (BMOE). The fraction of cross-linked Smc species was quantified by in-gel detection of fluorescently labeled Smc-HaloTag protein (Figures S2B–S2D) (Bürmann et al., 2013). In total, we screened 440 aa positions in Smc (excluding heptad ''a'' and ''d'' residues) and identified out of these more than 80 residues giving strong cross-linking (>20% cross-linked dimers). The distribution of these putative axial residues over the length of the Smc coiled coil follows a distinctive pattern (Figure 2A). In the N-terminal coiled-coil helix, several stretches display efficient cysteine cross-linking: near the Smc hinge domain (residues 466–501), around position 395 (residues 372–419), and around position 300 (residues 266–331). Between these stretches, however, cross-linking of N-terminal residues was virtually undetectable, while selected positions on the corresponding section of the C-terminal α helix displayed robust cross-linking. The striking pattern strongly suggest that the two long Smc coiled coils adopt defined conformations in the Smc-ScpAB holo-complex in vivo. The findings are consistent with a side-by-side alignment of the Smc coiled coil over the length of almost 300 α-helical residues from the Smc hinge (residues 216–501) (Figure 2A).

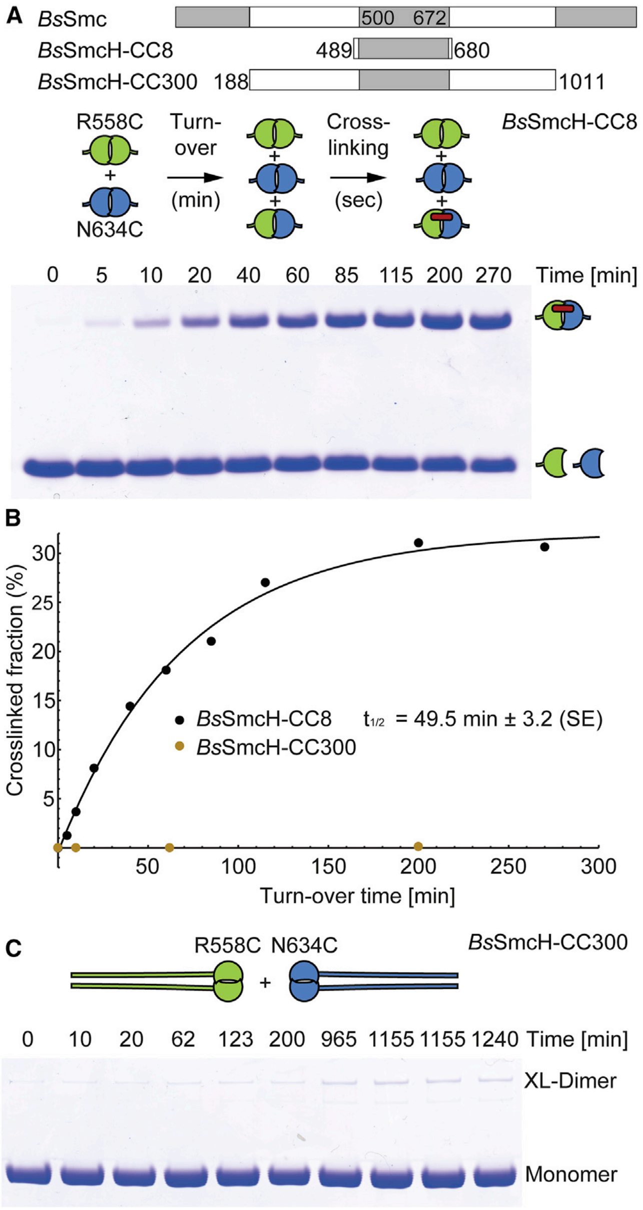

Figure 1. Turnover of Smc Protein Fragments

(A) Equimolar mixtures of *Bs*SmcH-CC8(R558C) and *Bs*SmcH-CC8(N634C) were incubated at 37°C and cross-linked by BMOE at indicated time points. Monomer and cross-linked dimer species were analyzed by electrophoresis and Coomassie staining. A representative image from three replicate experiments is shown.

(B) Quantification of turnover of *Bs*SmcH-CC8 and *Bs*SmcH-CC300 using an Agilent 2100 Bioanalyzer. Cross-linking efficiency is given as fraction of cross-linked protein to total protein. The data points were fitted to an exponential curve. The half-life is given as mean from three replicate experiments. Its standard error (SE) is denoted.

(C) Same as in (A) using *Bs*SmcH-CC300(R558C) and *Bs*SmcH-CC300(N634C) proteins.

See also Figure S1.

In the head-proximal region of Smc (residues 180–260 and 920–1,010), however, the pattern of cross-linking is more irregular. The structure of the Smc rod thus appears to deviate from a coils/coils alignment (Figure 2A). Crucially, several residues on the Smc head also cross-link efficiently to their symmetry mates, indicating that the two Smc head domains are frequently juxtaposed in Smc-ScpAB.

The Rod-Shaped Middle Segment of the Smc Coiled Coil

To elucidate the molecular basis for the formation of the Smc rod, we performed crystallographic studies on *Bs* and *Pyrococcus* Smc coiled coils. Several attempts with full-length Smc coiled coils only yielded poorly diffracting crystals. Taking advantage of recently gained knowledge on the register of the *Bs* Smc coiled coil, we thus designed shorter *Bs* Smc fragments (Minnen et al., 2016; Waldman et al., 2015). A structure of a Smc middle segment, designated as *Bs*SmcCC2, comprising residues 246–379 and 793–929 connected via a short linker (Figure 2A), was solved by crystallography at a resolution of 3.2 Å (Table S1). The *Bs*SmcCC2 monomer comprises two ~120 residues long α helices (designated as αN1 and αC1) (Figure S2E), whose arrangement presents the knob-into-hole organization of a canonical coiled coil.

The asymmetric unit is formed by two dimers of *Bs*SmcCC2 (Figure S2F). In each dimer, the two molecules are aligned side by side along a pseudo-symmetry axis (Figure 2B), which is reminiscent of the hinge-proximal Smc rod structure (Soh et al., 2015). In the middle of *Bs*SmcCC2, residues of αN1 located in heptad positions "b," "e," and "f" are involved in the contact between the two coiled coils via charged and hydrophobic side chains. At the top of the structure, residues of αC1 form a similar dimer interface. To establish whether the organization of the *Bs*SmcCC2 dimer in the crystal is compatible with the cysteine cross-linking pattern described above (Figure 2A), we mapped the cross-linking efficiency onto the *Bs*SmcCC2 structure and plotted it against the corresponding Cα-Cα distances (Figure 2B, right and bottom panels, respectively). The excellent agreement between the crystal structure and the in vivo cross-linking pattern implies that both datasets faithfully represent the architecture of Smc-ScpAB complexes in vivo.

The Smc Joint—a Head-Proximal Interruption in the Smc Coiled Coil

Next, we solved the crystal structure of a head-proximal Smc segment, named *Bs*SmcCC1, at a resolution of 1.9 Å (Table S1). While the N-terminal α helix αN1 (residues 188–253) is continuous, albeit being considerably bent, the C-terminal part (residues 922–1,011) splits into four separate α helices (Figure 3A).

The top and bottom helices αC1 and αC4, respectively, form coiled-coil contacts with the top and bottom segment of αN1 in the *Bs*SmcCC1 structure (Figure 3A). Helix αC2 lies perpendicular to αN1 to connect helix αC1 with αC3, which is located on the opposite side of αN1 (Figure 3B). Helix αC3 makes a coiled-coil-like interaction with the middle part of αN1. A short loop joins αC3 to αC4 across αN1.

Overall, *Bs*SmcCC1 folds into a peculiar, elongated structure, where C-terminal sequences form three helix-loop repeats that

CellPress

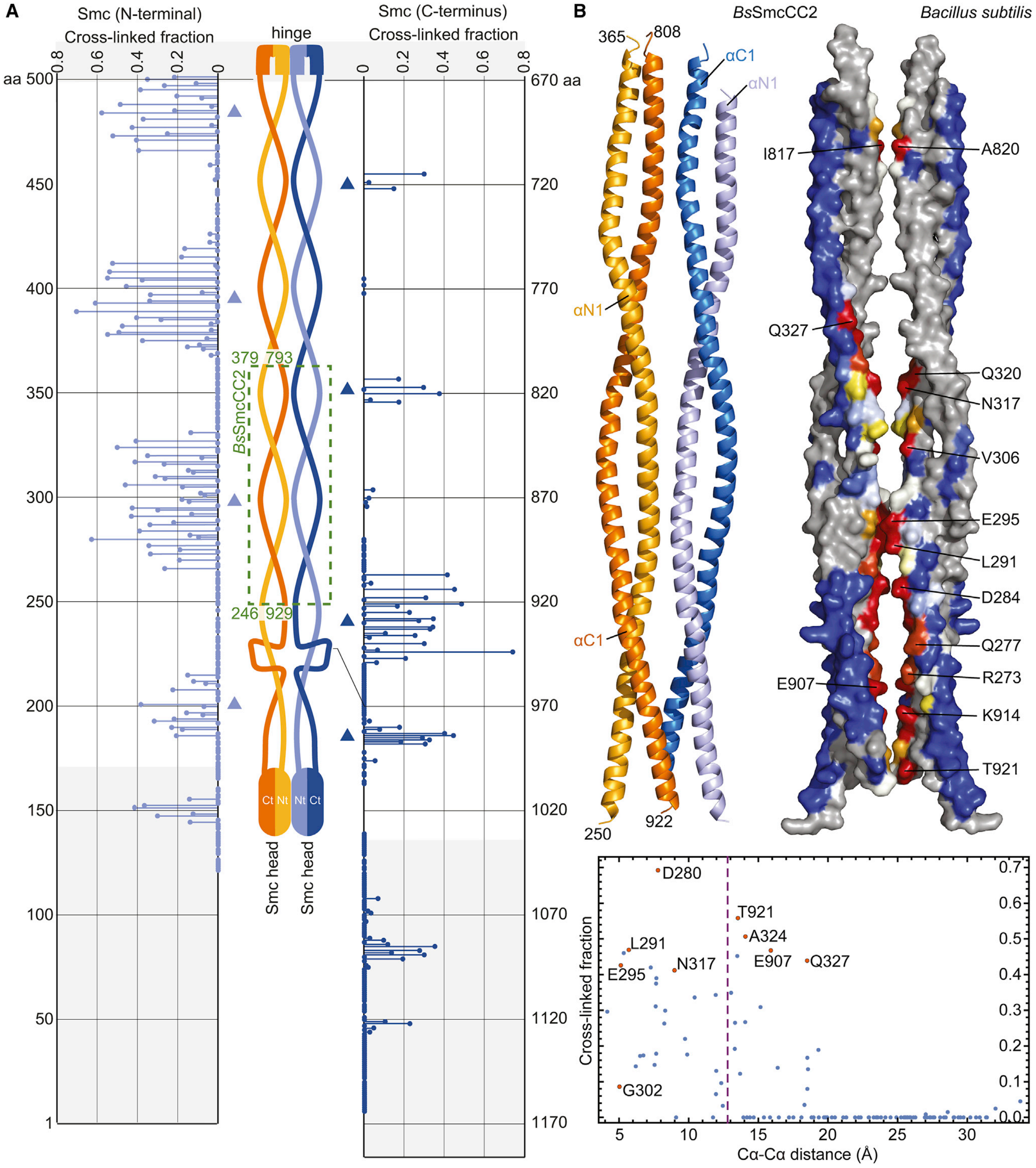

Figure 2. Mapping of the Smc Rod by Cysteine Cross-Linking and Structural Analysis

(A) HTP cysteine cross-linking. Schematic representation of the bacterial Smc homodimer (middle). Cross-linking efficiency is given as fraction of cross-linked to total Smc-HaloTag species. For N- and C-terminal Smc(Cys) residues, the data points are displayed on the left and right graphs in light and dark blue colors, respectively. Head and hinge regions are indicated by gray shading. Triangles in light and dark blue colors denote interfaces at N- and C-terminal Smc regions, respectively. A table with corresponding cross-linking efficiencies is available (Table S4).

(B) The structure of a middle segment of the *Bs* Smc rod reveals the longitudinal alignment of the Smc coiled coils. Cartoon representation of the structure of the *Bs*SmcCC2 dimer in side view (left). Monomers are displayed in orange and blue colors, respectively. Mapping of in vivo cross-linking efficiency onto *Bs*SmcCC2

(legend continued on next page)

spirally contact the continuous α helix αN1. We term the coiled-coil interruption "Smc joint," as it connects two straight parts of the Smc protein at a slight angle. A crystal structure of a yeast cohesin Smc3 head (PDB: 4UX3) overlaps with the bottom part of the *Bs*SmcCC1 structure (Figure S3A) (Gligoris et al., 2014), showing a highly similar structural organization. Moreover, several loop residues are highly conserved between prokaryotic Smc and cohesin Smc3, implying that the SMC joint is of fundamental importance for SMC function (Figure S3A).

Crystal packing does not involve the formation of a "rod-like" dimer of *Bs*SmcCC1. To establish how two *Bs*SmcCC1 monomers might be arranged within the Smc dimer, we mapped cysteine cross-linking efficiencies onto the structure of *Bs*SmcCC1 (Figure 3C; Figure S3B). The axial residues are distributed over the entire length of *Bs*SmcCC1 but are excluded from αC2 and its adjacent peptides. Importantly, almost all side chains of these residues point toward the same face of the elongated *Bs*SmcCC1 structure (Figure 3C) as is expected for an element of a rod-shaped Smc dimer (Figure S3B).

To understand how the Smc joint connects to the Smc head at the end of the Smc rod, we next solved the structure of *Py*SmcHd-CC80, a *Pyrococcus yayanosii* Smc head attached to a long coiled coil that includes the Smc joint (Figure 3D). The highly elongated structure superimposes well with the *Bs* Smc head (PDB: 3ZGX) and the *Bs* Smc joint (*Bs*SmcCC1) (Figure S3C). Based on sequence and structure comparison (Figures S3C and S3D), we mapped the *Bs* Smc cross-linking efficiencies onto *Py*SmcHd-CC80 (Figure 3D, right). Remarkably, these residues form a straight line on the surface of the molecule, the presumptive 2-fold symmetry axis of the Smc rod. We then manually aligned two *Py*SmcHd-CC80 molecules aiming to minimize the distance between all cross-linkable residues (Figure 3E). The resulting Smc rod fragment shows an intimate association between the two *Py*SmcHd-CC80 monomers with little steric clashes. While the Cα-Cα distance is relatively large (>20 Å) for some residues, indicating that the model of the *Py*SmcHd-CC80 dimer is not a perfect representation of the structure of native *Bs* Smc-ScpAB, overall there is excellent alignment of most pairs of experimentally identified *Bs* Smc axial residues.

Reconstruction of Smc Rods

Encouraged by the fitting of the *Py*SmcHd-CC80 dimer, we next aimed to obtain a reliable atomic-resolution model for the entire Smc rod. To do so, we first determined the structure of the upper region of the *Py* Smc coiled coil, designated as *Py*SmcCC3, comprising residues 345–468 and 694–814 connected via a SGGS linker (Table S1). The structure refined to 2 Å resolution showed that, while the N-terminal α helix is continuous, the C-terminal helix contains a short non-helical region (Figure S4A). This region (residues 774–779) exhibits barely traceable electron densities, and the opposing portion of the N-terminal helix displays better, but weak, electron densities. Helical wheel analysis and sequence alignment indicate that the non-helical region in *Py* Smc arises from a 2 aa deletion in comparison with other Smc protein, e.g., *Bs* Smc, which completes a heptad repeat in this region (Figure S4A, right). In addition, the N-terminal helix bends noticeably at residue P434, which is unlikely to bear functional importance since P434 is not a conserved residue (Figure S4A).

To reconstruct the entire Smc structure, two copies of *Py*SmcCC3 were superimposed onto the *Pf*SmcH-CC60 structure (RMSD of 0.66 Å for 39 Cα atoms), comprising the *Pf* Smc hinge and its proximal coiled coil (PDB: 4RSJ) (Soh et al., 2015), using an overlap between the structures (residues 446–465 and 696–719) (Figure 4A). Next, we generated a *Pf* version of the homodimeric *Bs*SmcCC2 structure by simple amino acid substitutions taking into account a single amino acid insertion (around residue 290) in the corresponding part of the *Pf* Smc protein (Figure S4B). The dimer was then superimposed and connected to the ends of the above generated dimer and to the ends of two copies of *Py*SmcHd-CC80 using the *Bs*SmcCC1 structure as a guide. Only slight adjustment was necessary at the top of the *Py*SmcHd-CC80 coiled coil to avoid steric crash (Figure S4D).

The reconstituted dimer displays a straight Smc rod with closely juxtaposed coiled coils (Figure 4B). There is a total of seven contact interfaces between the two coiled coils in the dimer. Up to the 6th coil-coil contact (counting from the hinge), the N- and C-terminal helices alternatingly contact each other with the coiled-coil pitch of ~170 Å. At the head side, however, this regularity breaks due to the presence of the Smc joint, resulting in an additional contact (7th interface) between both N- and C-terminal segments of the two Smc joints. Importantly, the Smc joint tilts the coiled-coil segments right below it, ~11° relative to the central axis, resulting in the close juxtaposition of the Smc head domains (Figure 4B). Without this tilting, steric clashes between the two head domains are unavoidable. Therefore, one critical function of the Smc joint might be to properly orient the head domains (see below). The structure reflects the data of the *Bs* in vivo cysteine cross-linking screen well, as efficiently cross-linked positions are generally found close to the central symmetry axis (Figure S4E). However, the two hinge-proximal stretches of cross-linkable residues in the N-terminal α helix are not perfectly centered on the corresponding contact regions in the archaeal reconstruction (Figure 4C). This suggests that there might be slight differences in the coiled-coil arrangement between the *Pf*SmcH-CC60 crystal structure and *Bs* Smc-ScpAB holo-complexes in vivo. Nevertheless, our data demonstrate the existence of a structurally well-defined Smc coiled coil, which has likely been maintained in bacteria and archaea, i.e., over a remarkably long period of evolution, thus underscoring its physiological relevance.

in surface representation (right). Residues are color coded according to the cross-linking efficiency given in (A): blue, yellow, orange, and red colors indicate no, low, medium, and high cross-linking efficiency, respectively. Residues colored in gray have not been tested. The identity of selected residues with medium and high cross-link efficiency is denoted. A graph displaying the cross-linking efficiency (from A) relative to the distance of respective Cα atoms in the *Bs*SmcCC2 structure is shown (bottom). For selected residues, data points are denoted and labeled in orange colors. A dashed line indicates the linker length of BMOE plus the length of two cysteine side chains.
See also Figure S2 and Table S5.

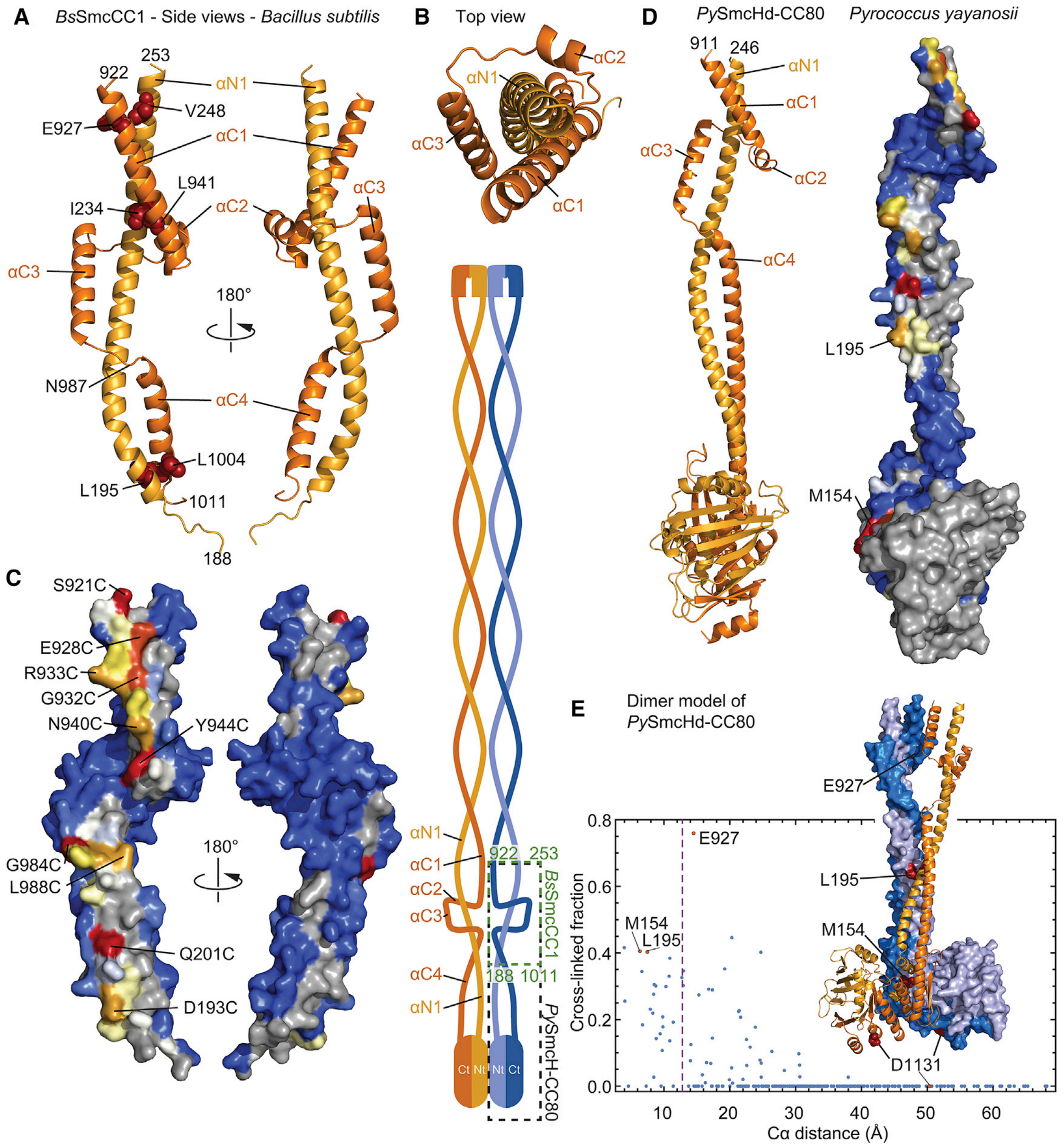

Figure 3. The Organization of the Head-Proximal Smc Joint

(A) Structure of *Bs*SmcCC1. Display and color coding as in Figure 2B. Front and back view are shown on the left and right, respectively.

(B) Top view of *Bs*SmcCC1. As in (A).

(C) Mapping of the in vivo cross-linking efficiency onto *Bs*SmcCC1. Surface representation of structural views shown in (A). Color coding as in Figure 2B.

(D) Crystal structure of *Py*SmcHd-CC80 shown in cartoon and surface representation on the left and right, respectively. Color coding as in Figure 2B. Mapping of the *Bs* Smc cross-linking efficiencies onto *Py*SmcHd-CC80 based on sequence alignments shown in Figure S4D. Please note that all cross-linking competent residues are arranged in a line on the surface of the molecule.

(E) Model of a *Py*SmcHd-CC80 dimer. Two copies of *Py*SmcHd-CC80 were aligned manually by minimizing the distance between cross-linking residues, thereby producing a tight dimer with little or no steric clashes. Monomers are displayed in cartoon and surface representation. Color coding as in Figure 2B. The graph displays the cross-linking efficiency of *Bs* Smc(Cys) residues given in Figure 2A versus the Cα-Cα distance of corresponding residues in the dimer model of *Py*SmcHd-CC80.

See also Figure S3 and Table S5.

Smc Heads Exist in Alternative Dimer States

We noticed that all residues on the Smc head domain, which were cross-linked by BMOE when substituted for cysteine (Figure 2A), map to a relatively small surface area (Figure 5A). This area overlaps with the ABC signature motif, implying that the signature motifs of the two heads are closely juxtaposed

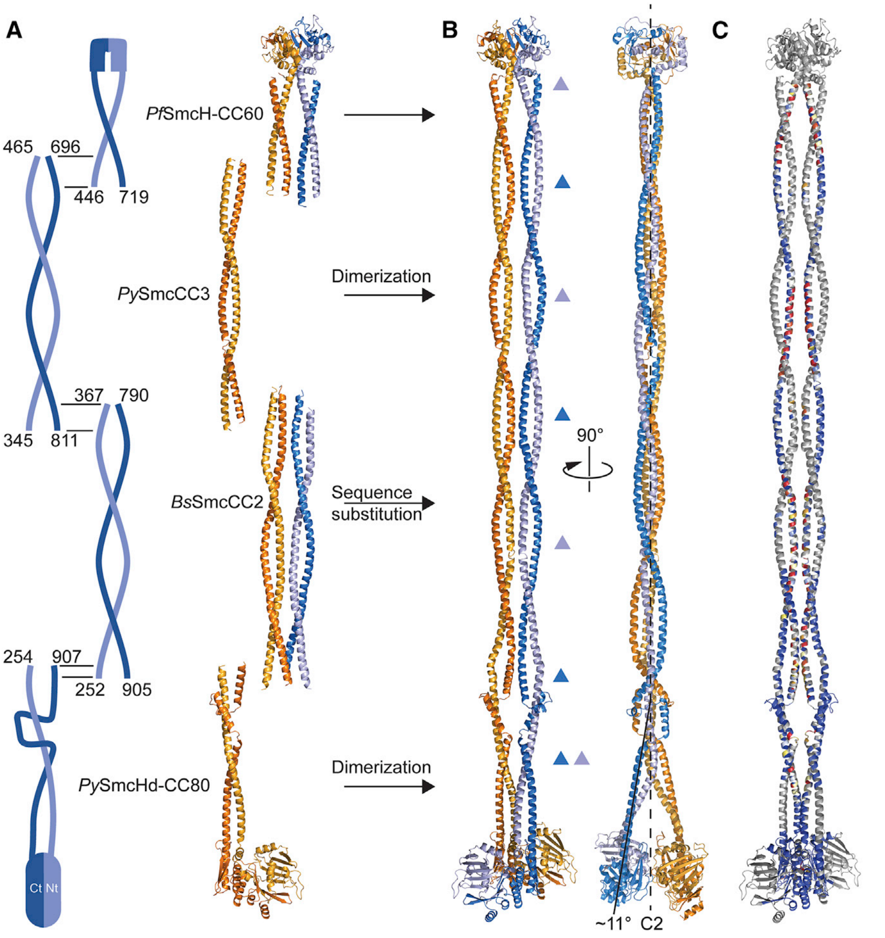

Figure 4. Construction of an Archaeal Smc Rod Model

(A) Schematic overview and cartoon representation of several Smc coiled-coil crystal structures used for Smc rod reconstruction. *Pf*SmcH-CC60 (PDB: 4RSJ) partially overlaps with *Py*SmcCC3 (Figure S4A). *Bs*SmcCC2 was transformed into a *Py* Smc model by sequence substitution. The product overlaps partially with *Py*SmcCC3 at its top and with *Py*SmcHd-CC80 at the bottom. All residue numbers correspond to the *Py* Smc sequence. Color coding as in Figure 2B.

(B) Reconstructed *Py* Smc rod in front and side views. Please note the angle in the otherwise straight Smc arm at the Smc joint (right). Color coding as in Figure 2B. The dashed line indicates the symmetry axis ("C2") of the Smc rod dimer. Triangles in light and dark blue colors denote interfaces at N- and C-terminal Smc residues, respectively (as in Figure 2A). The coordinates of full-length Smc are available in supplemental material (Table S6).

(C) Mapping of *Bs* Smc cross-linking onto the *Py* Smc rod model. Color coding as in Figure 2B using *Bs* and *Py* Smc sequence comparisons (Figures S3D and S4C).

See also Figure S4 and Tables S5 and S6.

and thus unable to align with their respective ATP-binding pocket.

To better define the structural differences between Smc heads aligned at the end of the Smc rod and those engaged via ATP, we solved another crystal structure: "*Bs*SmcHd-CC30:ATPγS-ScpA^{C}" comprising ATPγS bound *Bs* Smc(E1118Q) heads with 30-residue coiled coils associated with the C-terminal winged-helix domain of *Bs* ScpA (ScpA^{C}) (Table S1). The arrangement of the two head domains in *Bs*SmcHd-CC30:ATPγS-ScpA^{C} shows a high degree of similarity (RMSD < 1 Å) with a previously solved structure of an ATP dimer of *Pf* Smc heads lacking coiled coils and ScpA (Lammens et al., 2004).

We measured the Cα-Cα distances for S152-S152′ (located on the Smc head) and D193-D193′ (located at the head-proximal coiled coil) on the *Bs*SmcHd-CC30 dimer at 21.7 and 77.0 Å, respectively (Figure 5B). These large distances are incompatible with their efficient cross-linking by BMOE, implying that cysteine cross-linking takes place on Smc dimers with disengaged heads. If so, then the cross-linking of S152C and D193C should be highly sensitive to changes in the levels of Smc head engagement (Minnen et al., 2016). While an ATP-binding mutation (K37I) and a head engagement (S1090R) mutation did not substantially alter S152C-S152C′ and D193C-D193C′ cross-linking, the presence of a Smc ATP hydrolysis mutation (E1118Q) roughly halved cross-linking at both positions (Figure 5D). When combined with a dimerization-deficient hinge ("mH"), cross-linking is further reduced to about 20% of wild-type levels, presumably due to its destabilizing effect on the Smc rod (Figure 5D) (Minnen et al., 2016). On the contrary, cross-linking of head residue K1151C shows a strong increase in cross-linking in E1118Q and mH-E1118Q, while it is barely detectable in K37I and S1090R (Figure 5D).

Smc heads must therefore exist in two distinctive dimer states. The ATP-engaged state (PDB: 1XEX) (Figure 5B) shows a high degree of similarity to the corresponding conformation of ABC transporters, while the other state likely closely resembles the one shown in the Smc rod model (Figure 4B). Here, the ABC signature motifs of the two head domains are closely juxtaposed. Engagement of heads within and between Smc dimers is blocked by misalignment of the ATP-binding motifs and by steric occlusion, respectively. To convert the rod-shaped into the ATP-engaged state, we need two operations: the sliding of Smc heads toward each other along an axis connecting the two ATP-binding pockets by about 10 Å and the tilting of one Smc head relative to the other head by about 85° around a similar axis (Figures 5B and 5C; Figure S5E; Movies S1 and S2). Both transformations bring about the dissolution of the Smc rod by positioning the head-proximal Smc coiled coils at a distance.

A Tentative Model for the Ring-like Smc Dimer

Finally, we derived a model for the organization of the ATP-engaged state of the Smc dimer. We superimposed two Smc

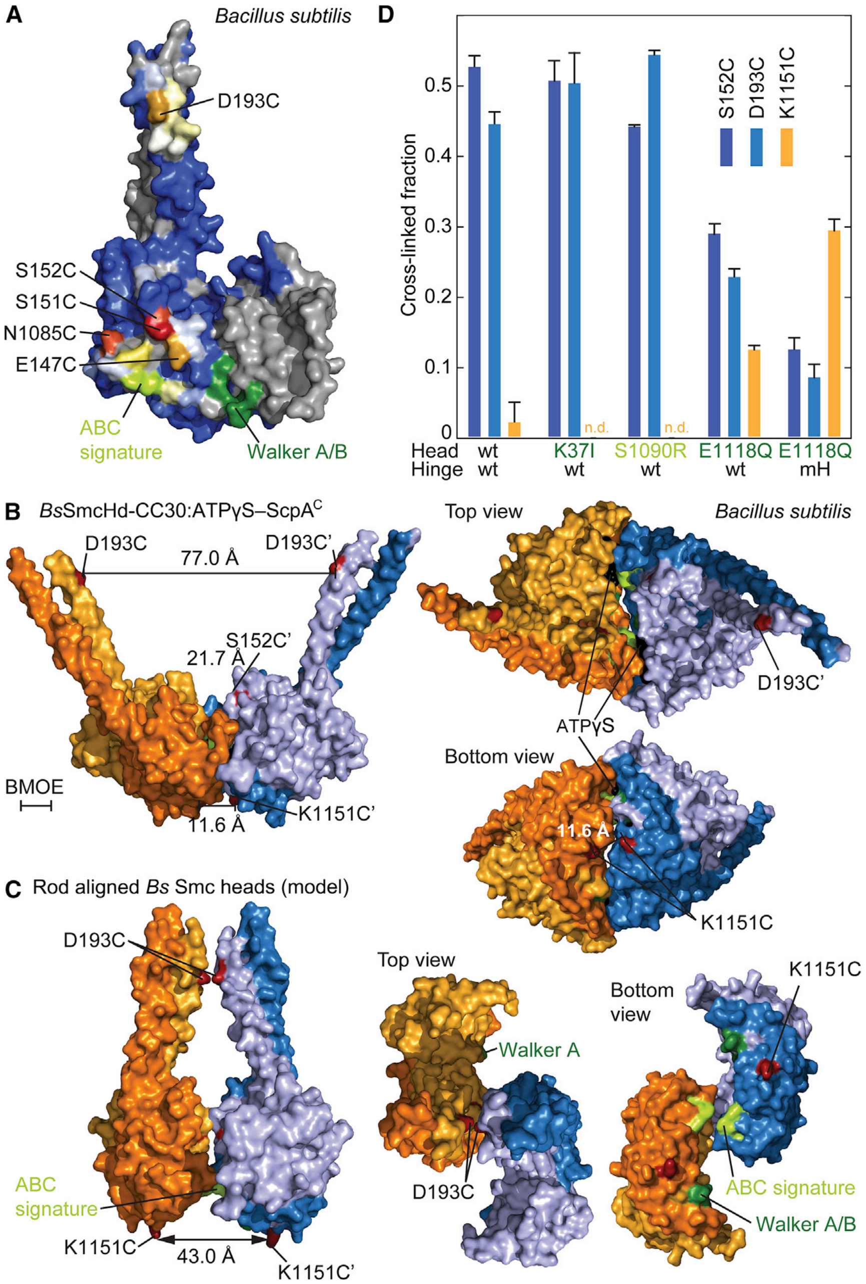

Figure 5. Distinct Dimer States of Smc Head Domains

(A) Mapping of Smc(Cys) cross-linking onto the *Bs* Smc head (PDB: 3ZGX). The N-terminal ScpA fragments is omitted from the view. Color coding as in Figure 2B. Selected residues displaying efficient cross-linking when mutated to cysteine are marked. In addition, residues of the ABC signature motif and the Walker box motifs are displayed in light and dark green colors, respectively.

(B) Crystal structure of *Bs*SmcHd-CC30:ATPγS–$ScpA^C$ in surface representation in front (left), top, and bottom views (right). The C-terminal ScpA fragments are omitted from all views. The Cα-Cα distance between pairs of selected residues (in red colors) across the ATP-engaged dimer is given. General color coding as in Figure 2B. ATPase motif residues are colored as in (A). ATPγS is displayed in spheres in black colors. For size reference, the scale of the cross-linker BMOE is displayed.

(C) A model of the *Bs* Smc head domain (PDB: 3ZGX) in the rod dimer configuration. The head dimer is constructed by superimposition onto the *Py* Smc rod structure (Figure 4B) and reflects the cysteine cross-linking data (Figure 2A) (see also Figure S5D). Display as in (B).

(D) In vivo cysteine cross-linking of Smc(Cys) proteins with wild-type and mutant ATPase domains. Cross-linking of S152C, D193C, and K1151C residues in Smc-HaloTag proteins bearing ATP binding (K37I), head engagement (S1090R), ATP hydrolysis (E1118Q), and hinge dimerization interface (G657A, G658A, G662A, G663A; "mH") mutations. In all strains, four endogenous cysteines were substituted for serines (Hirano and Hirano, 2006). Cell extracts were labeled with HaloTag-TMR substrate. Smc-HaloTag species were separated by SDS-PAGE and quantified by in-gel fluorescence scanning. Data are represented as mean values and standard deviation from biological replicates (duplicates containing technical triplicates).

See also Figure S5, Table S5, and Movies S1 and S2.

monomers taken from the reconstructed *Pf* Smc rod onto the structures of the ATP-engaged dimer of *Pf* Smc heads. The structure represents an open-arm conformation (Figure 6A). Intriguingly, while the Smc joint allows Smc heads to juxtapose at the end of the Smc rod, in the head-engaged state, it drives the coiled coils further apart by tilting them away from the symmetry axis (Figure 6A; Figure S6B). The joint might thus help to propagate rod dissolution from the Smc heads to the hinge domains.

Wide open coiled-coil arms immediately suggest that hinge domains detach from one another upon head engagement or that significant bending of the coiled coils occurs to generate ring structures instead of open dimers (Figure S6A). To discriminate between the two possibilities, we tested for simultaneous engagement of hinge and head domains using cysteine cross-linking. The introduction of cross-linkable cysteines into the hinge of a strain with a cross-linkable head interface produced additional slowly migrating species of Smc—presumably including covalently closed rings—in wild-type Smc and Smc(EQ) (Figure 6B; Figure S6C). In addition, the fraction of Smc cross-linked at heads only was significantly reduced in this strain, consistent with a conversion of this species into a double cross-linked form. These results indicate that *Bs* Smc proteins can form hinge domain dimers when the heads are engaged by ATP. However, they do not rule out the possibility that this is an energetically unfavorable process and that a certain, probably very small, fraction of Smc dimers might open the hinge when heads are engaged.

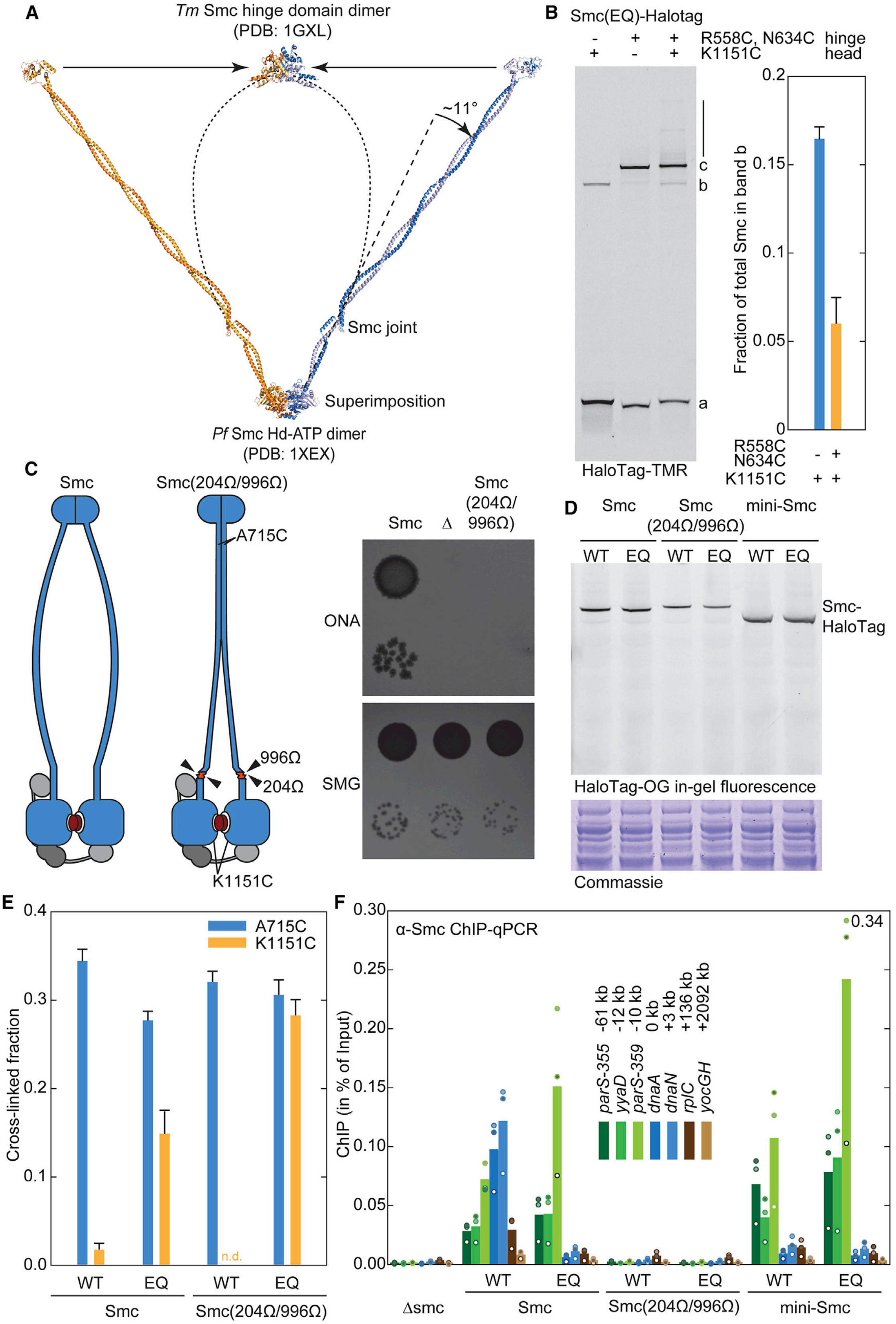

(legend on next page)

To create a model of ring-shaped Smc dimers, we introduced continuous ~9° bending over six α-helical turns to the *Pf* Smc coiled coil beyond the Smc joint. Additionally, slight inward tilting of the hinge-proximal coiled coils was used to connect the coiled coils at the hinge (Figure S6A). This model was subjected to energy minimization. The resulting model maintains the α-helical structure with reasonable stereochemistry (Figure S6B). This open-ring conformation fully exposes the positively charged surface at the bottom of the hinge, which is presumed to bind DNA (Hirano and Hirano, 2006; Soh et al., 2015). Such a ring structure is likely under significant tension due to the rigidity of the Smc coiled coil but is stabilized by the tight hinge-hinge and head-head interactions. If so, then Smc heads likely are rapidly torn apart upon ATP hydrolysis.

Artificial Uncoupling of Smc Head Engagement and Rod Dissolution Blocks Chromosomal Localization

Targeting of Smc-ScpAB to chromosomal loading sites depends on Smc head engagement, which conceivably supports rod dissolution and thereby allows for ParB/*parS* interactions (Minnen et al., 2016). The mechanical dissolution of the Smc rod as proposed above necessitates some level of rigidity in the Smc arm. Loss of arm rigidity may therefore uncouple head engagement from rod dissolution and block targeting of Smc to the chromosome. Consistent with the notion of rigid Smc arms, we recently found, by a random peptide insertion screen, that the arms are particularly sensitive to the insertion of peptide sequences at any position except in parts of the Smc joint domain and at the hinge-proximal end of the coiled coil (Bürmann et al., 2017). Two such insertions (at residue 394 and 479), however, do not interfere with the initial recruitment of Smc to the chromosome but rather block the downstream event of Smc relocation from *parS* sites (Bürmann et al., 2017). This suggests that arm rigidity is either dispensable for chromosome targeting or that the two tested peptide insertions do not sufficiently compromise arm rigidity to prevent rod dissolution. To discriminate between these possibilities, we constructed a Smc protein with two peptide insertions: one insertion in each α helix located at corresponding positions between the head and the joint domains (Figure 6C). The double peptide insertion renders Smc non-functional, but the protein accumulates at near wild-type levels, being indicative of proper protein folding (Figures 6C and 6D). Notably, the double insertion markedly increases the fraction of Smc(EQ) dimers with engaged Smc head domains (Figure 6E). The improved efficiency of head engagement in double insertion Smc(EQ), however, does not translate into opening of the Smc rod as measured by cysteine cross-linking of residue A715C located at the arm/arm interface near the Smc hinge (Figure 6E). These findings support the notion that rigidity in the Smc arm is required for rod dissolution. Importantly, the mutant protein fails to localize to the chromosomal loading site at *parS-359* or to other chromosomal loci as determined by ChIP-qPCR (Figure 6F; Figure S6D), despite the high levels of head engagement. These results demonstrate that head engagement—albeit being essential—is not sufficient for chromosome targeting and that Smc arm integrity is critical in this process. Together, our results support the view that Smc arms mechanically promote chromosome targeting and relocation using distinct mechanisms: chromosome targeting is relatively robust, only being blocked by a double insertion in the Smc arm (Figure 6F), while Smc relocation is sensitive to single peptide insertions as well as to illegitimate Smc arm shortening (Bürmann et al., 2017).

DISCUSSION

Here, we established the overall architecture of the prokaryotic Smc rod and elucidated a conformational switch at the Smc head domains that uses the energy from ATP binding and hydrolysis to drive large-scale transitions between a rod and a ring state. The direct coupling of Smc head engagement and rod

Figure 6. Opening of the Smc Rod Is a Prerequisite for Chromosomal Targeting

(A) Superimposition of two Smc monomers taken from the reconstructed Smc rod (Figure 4B) with an ATP dimer structure of the *Pf* Smc head (PDB: 1XEX). Significant bending of the coiled coils is required to generate a closed ring-shaped Smc dimer. The Smc joint bends the Smc coiled coil away from the central symmetry axis by about 11°.

(B) ATP engagement of Smc heads in Smc proteins with intact hinge dimers. Smc(EQ)-HaloTag cells with cysteines at the hinge and/or head were cross-linked by BMOE. HaloTag protein was labeled by Halo-TMR and analyzed by SDS-PAGE (left). Smc monomer (a), head-head dimer (b), and hinge-hinge dimer (c) bands are marked. The vertical line denotes additional species appearing in the presence of hinge and head cysteines. These likely represent circular species as well as larger oligomers. Please note that the contrast is enhanced to display low-abundance species in the figure. Quantification of the fraction of Smc protein in the head-head band (b) is shown (right). Mean and standard deviation were calculated from two biological replicates. An analogous experiment with wild-type Smc ATPase in shown is Figure S6C.

(C) Double insertion of peptide sequences (in red color) interferes with Smc function. Schematic view (left). Colony formation of strains harboring wild-type Smc, a Smc in-frame deletion ("Δ"), or a Smc with peptide insertions ("204Ω/996Ω") at position 204 (residues SGPGGGGGRQVEP) and at position 996 (residues SGPGGGGGRQFER) on nutrient-poor (SMG) and nutrient-rich (ONA) medium.

(D) Cellular expression of Smc proteins harboring modified coiled coils. Smc-HaloTag proteins were labeled in crude *Bs* cell extracts by HaloTag-Oregon green and analyzed by in-gel fluorescence. To control for protein extraction, we stained an equivalent protein gel with Coomassie (bottom). "EQ" denotes the Smc(E1118Q) ATP hydrolysis mutation. "Mini-Smc" indicates a non-functional Smc variant with shortened coiled coil (CC293; Bürmann et al., 2017).

(E) In vivo cysteine cross-linking of Smc proteins harboring peptide insertions. Cross-linking of A715C and K1151C residues in Smc-HaloTag proteins bearing peptide insertions at positions 204 and 996. Data generation and display as in Figure 5D.

(F) Chromosome localization of Smc proteins with modified coiled coils. ChIP was performed with an antiserum raised against *Bs* Smc. Selected genomic positions were analyzed by quantitative PCR. The amount of ChIP DNA is given as percentage of input. The mean was calculated from three biological replicates. Data from individual experiments are displayed as dots with white, light gray, and dark gray filling, respectively. The same strains are also used in (D) and (E) and harbor a C-terminal HaloTag and the K1151C mutation. Equivalent results were obtained with a set of strains with untagged Smc and lacking additional Smc mutations (Figure S6D).

See also Figure S6.

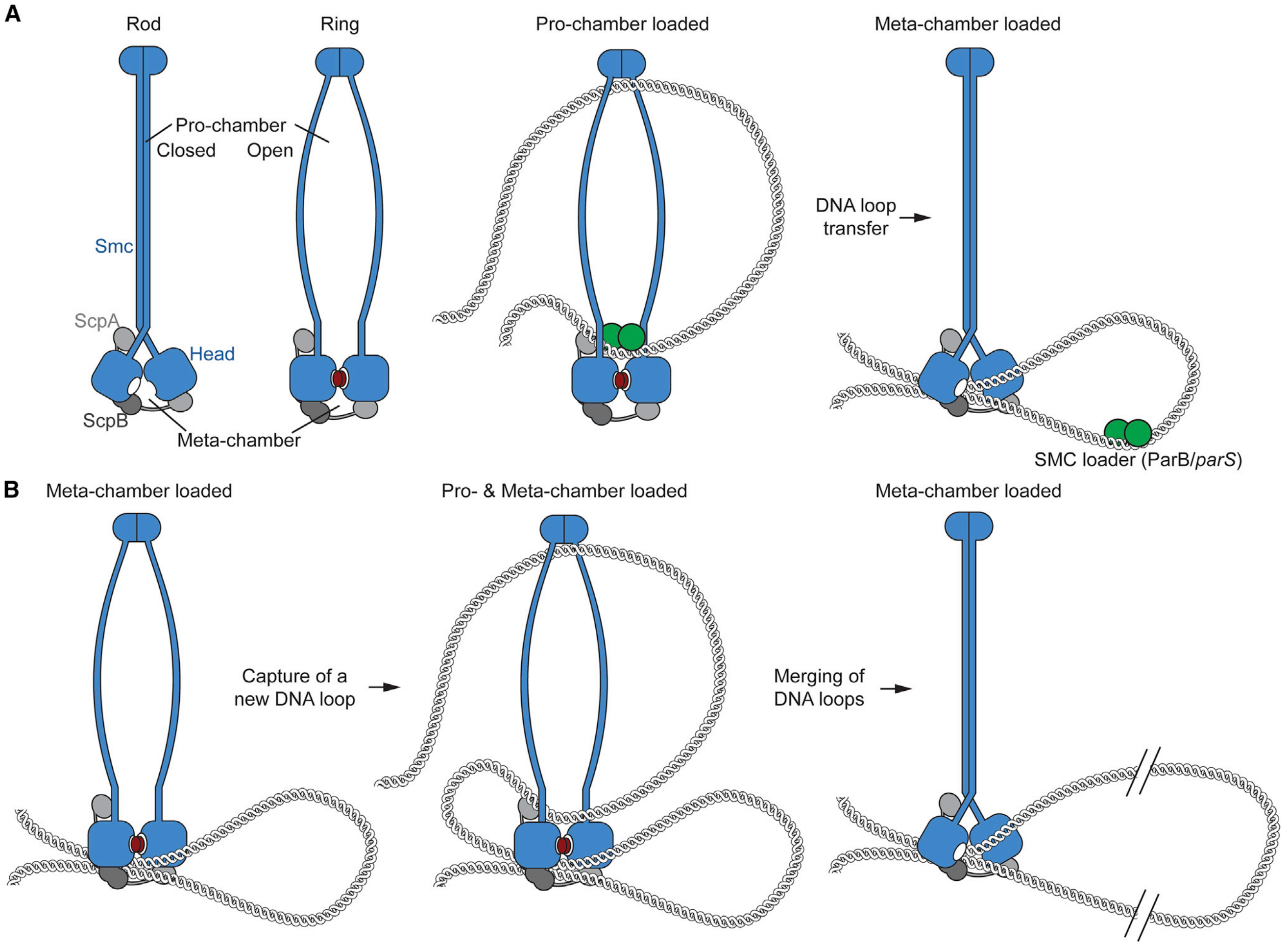

Figure 7. A Tentative Model for DNA Loop Extrusion by a Double-Chamber SMC Complex

(A) Chromosomal loading. The head-engaged open form of Smc-ScpAB initiates chromosomal DNA transactions by capturing a DNA loop in its DNA pro-chamber, i.e., the Smc interarm space. The loading machinery (in green colors) provides specificity by binding to the open Smc arms. ATP hydrolysis destabilizes the open Smc ring and triggers re-formation of a Smc rod starting from the Smc hinge. Rod formation closes the pro-chamber and pushes DNA into the meta-chamber, i.e., the inter-head/kleisin/kite space.

(B) Processive DNA loop extrusion. After successful loading, the subsequent loop capture-merging cycles start with the engagement of the Smc heads to open up the pro-chamber for DNA loading. To drive directional DNA extrusion, the newly captured DNA must be derived from DNA flanking the already captured DNA loop rather than the DNA loop itself. The asymmetry of Smc-ScpAB might ensure the directionality of this step. Upon ATP hydrolysis, the newly captured DNA is then merged with the DNA loop previously loaded into the meta-chamber to generate a larger DNA loop. A related model for DNA translocation of SMC double-ring "handcuff" complexes is shown in Figure S7.

See also Figure S7.

dissolution provides a simple means to regulate DNA binding at the distantly located Smc hinge (Soh et al., 2015) and targeting to the chromosomal *parS* loading sites (Minnen et al., 2016). Our results offer a first glimpse into the mechanochemical action of SMC proteins and, together with other recent findings, allow us to propose a molecular mechanism for DNA loop extrusion by SMC (Figure 7).

Distinct Conformations of SMC-Kleisin Complexes

SMC-kleisin complexes of different types and diverse origins have been visualized as open ring- and closed rod-like structures by electron and atomic force microscopy, often with a clear preference for one or the other form. While others have attributed the perceived structural heterogeneity to an intrinsic flexibility of the SMC coiled coils, we argue here that the prokaryotic Smc complex exists in at least two well-defined conformations with unique features and specific functions. Based on cysteine cross-linking of wild-type and ATPase mutant Smc proteins, we conclude that the prevalent form of Smc-ScpAB in vivo is a straight rod with juxtaposed, but misaligned, Smc head domains, while the more open conformation with engaged Smc heads appears to be rare under normal conditions. The strict functional requirement for ATP hydrolysis implies that the latter must exist at least transiently during the Smc ATPase cycle. It

presumably serves as an essential intermediate, which is involved in chromosome targeting (Minnen et al., 2016) and possibly also processive relocation.

What might be the functional role of such striking conformational changes? SMC complexes processively move along chromosomal DNA to bring together DNA distantly flanking the loading sites (Minnen et al., 2016; Wang et al., 2017). Switching between the rod and the ring conformation may drive the extrusion of DNA by SMC complexes via the capture and merging of DNA loops as described below (Figure 7). Prior to any loop extrusion, SMC complexes must target to rare chromosomal loading sites. To get to these sites, SMC complexes must exhibit high affinity for the recruitment machinery. Release from the loading sites, on the other hand, requires the elimination of a pro-formed and presumably tight interaction. The structural transitions described here might govern the switching between high and low affinity for the loading complex. Consistent with this notion, a segment of the Smc arm is critical for targeting of Smc to *parS* sites (Minnen et al., 2016).

A Ring-like Intermediate for DNA Loop Capture

In our model of the Smc rod, the Smc protein forms a straight object. When superimposed onto ATP-engaged Smc heads (or the open Smc hinge structure), such Smc proteins give rise to a wide open V-shaped structure (Figure 6A). If the coiled coils were rigid, then head engagement would require prior opening of the Smc-ScpAB ring by the detachment of the Smc hinge domains (Buheitel and Stemmann, 2013; Gruber et al., 2006). However, cysteine cross-linking experiments support the notion of simultaneous engagement of hinge and heads within a Smc dimer (Figure 6B). Thus, opening of the hinge does not seem to be a prerequisite for head engagement. Without hinge opening, significant deformation of the straight Smc protein must occur prior to or during the engagement of Smc head domains. These deformations will presumably lead to a wide open Smc ring structure, which might be ideally suited for the capture of large chromosomal DNA loops, presumably supported by DNA-binding surfaces located at the inner surface of the ring at the hinge and at the head domains (Hirano and Hirano, 2006; Soh et al., 2015; Woo et al., 2009). While we put forward a possible ring structure based on available crystal structures (Figure S6A), the exact nature of the head-engaged ring conformation(s) remain(s) unknown. Rotary-shadowed electron micrographs of purified *Bs* Smc dimers and holo-complexes frequently display ring-like structures with apparently engaged heads and hinges (Kamada et al., 2013, 2017; Melby et al., 1998). In these images, the coiled coils often display kinks rather than continuous bending. Such kinks could either help to dissipate or alternatively amplify strain within the arms. The strain will promote opening of the interarm space and may in addition destabilize the Smc hinge dimer to allow for DNA entry (Buheitel and Stemmann, 2013; Gruber et al., 2006).

A Rod-Shaped Smc Intermediate for DNA Loop Transfer

We have reconstructed the structure of a rod-shaped prokaryotic Smc dimer from four crystal structures and validated its overall architecture by cross-linking data. The two coiled coils of a Smc dimer are arranged side by side over 330 residues, creating seven contact regions alternatingly located on the N- and C-terminal helices. Together, these contacts probably hinder the dissociation of the Smc hinge observed in vitro (Figure 1) and contribute to the dimerization of hinge-less or hinge mutant Smc in *Bs* (Minnen et al., 2016). At least when kept at high local concentration by the Smc hinge, the two Smc arms are stably anchored onto each other, thus fixing the Smc head domains in a misconfiguration blocking ATP-dependent head engagement.

The high prevalence of the rod-form implies that at least a fraction of chromosomal Smc-ScpAB adopts a rod-like conformation too. Strikingly, chambers large enough to accommodate a DNA double helix are absent in our model of the Smc rod. We imagine that the rod conformation plays a critical role during the processive extrusion of DNA loops by SMC (Bürmann et al., 2017). The closure of the Smc arms upon ATP hydrolysis may dislodge DNA double helices from the interarm space and push them past the Smc head domains into a chamber formed by the Smc heads and the ScpAB sub-complex. The latter may conceivably be dedicated to the safekeeping of DNA loops (Figure 7) or DNA double helices (Figure S7). During most of Smc's mechanochemical cycle, the close juxtaposition of the Smc heads may keep this chamber shut to prevent DNA from escaping (Figure 7). Only during ATP hydrolysis, the head gate may transiently open to allow for the passage of DNA from the interarm pro-chamber to the meta-chamber. The directionality of DNA transport may be determined by rod formation starting from the hinge, but not the heads, possibly explaining the observed tight restrictions on Smc arm length (Bürmann et al., 2017). Alternating between the capture of new DNA loops and the merging of two pre-existing DNA loops driven by engagement and disengagement of Smc heads can provide a simple means for the stepwise addition of DNA segments to an ever-growing DNA loop (Figure 7) or for the step-by-step translocation of Smc along a chromosomal DNA double helix (Figure S7). In addition, the entrapment of DNA within a tight channel may prevent DNA tracking motors from slipping through the Smc-ScpAB complex. Thus, their movement along DNA can be harnessed by Smc-ScpAB to further support the DNA extrusion process. The asymmetry of Smc-ScpAB may ensure capture of new DNA segments only from one side of the ring to drive directional DNA transport (Bürmann et al., 2013).

Implications for Other SMC-Kleisin Complexes

It is currently unclear whether all SMC complexes form rod-like structures. In case of condensin in eukaryotes, the evidence supporting the prevalence of rods is solid, being based on several independent experimental approaches (EM, crystallography, cysteine, and lysine cross-linking) (Anderson et al., 2002; Barysz et al., 2015; Soh et al., 2015). Cohesin (like Smc-ScpAB) frequently adopts ring-like architectures during rotary-shadowing EM, while negative stain EM images, lysine cross-linking data, and single-molecule experiments are consistent with cohesin rods (Anderson et al., 2002; Huis in 't Veld et al., 2014; Stigler et al., 2016). Since Smc-ScpAB is frequently observed in V- or O-shaped configurations by rotary shadowing (Kamada et al., 2013, 2017; Melby et al., 1998), despite appearing to preferentially adopt I-shapes in vivo (Figure 5), this technique might have an intrinsic bias for open forms of Smc-ScpAB

and possibly also cohesin. Recently, the SMC-like Rad50 protein and the Smc5/6 complex have been proposed to form similar rods based on crystal structures of the respective dimerization domains (Alt et al., 2017; Park et al., 2017).

Regardless of any structural details, we propose that all SMC-kleisin complexes harbor two distinct states: an intrinsically favorable conformation, which is incompatible with ATP-dependent head engagement, and a shorter-lived conformation that is stabilized by ATP sandwiching between the two SMC heads. At least in Smc-ScpAB, the latter may capture a new DNA segment, while the former may merge a newly captured DNA segment with a pre-existing DNA loop. Recurrent switching between such states may thereby suffice to drive chromosomal loading and organization in all domains of life. Our work should facilitate related structural and mechanistic studies on other SMC and SMC-like protein complexes.

STAR★METHODS

Detailed methods are provided in the online version of this paper and include the following:

- KEY RESOURCES TABLE
- CONTACT FOR REAGENT AND RESOURCE SHARING
- EXPERIMENTAL MODEL AND SUBJECT DETAILS
  - *Bacillus subtilis* Strains and Growth
- METHOD DETAILS
  - Protein Turnover Measurements
  - High-Throughput Allelic Replacement Screening
  - In Vivo Cysteine Cross-Linking
  - Protein Production
  - Crystallization, X-ray Data Collection, and Structure Determination
  - Energy Minimization Calculation for the Ring-Shaped *Pf* Smc Dimer
  - Chromatin Immunoprecipitation
  - Colony Formation Assay
- QUANTIFICATION AND STATISTICAL ANALYSIS
  - Analysis of Cross-Linking Efficiencies
  - Analysis of Chromatin-Immunoprecipitation Efficiencies
- DATA AND SOFTWARE AVAILABILITY

SUPPLEMENTAL INFORMATION

Supplemental Information includes seven figures, eight tables, and two movies and can be found with this article online at http://dx.doi.org/10.1016/j.molcel.2017.06.010.

AUTHOR CONTRIBUTIONS

M.-L.D.-D., structures of *Bs*SmcCC1 and *Bs*SmcCC2 with support from J.B.; M.-L.D.-D., ChIP experiments; L.B.R.A., HTP genetic engineering and cysteine mapping with help from A.B.; A.B., generation of Smc insertion construct; H.L., structure of *Py*SmcHd-CC80 and reconstructions; H.N., structure of *Py*SmcCC3; H.-C.S., structure of *Bs*SmcHd-CC30:ATPγS-ScpA^{C}; F.B., development of HTP genetic engineering and modeling; F.P.B., protein turnover; A.D., protein purification; H.I. and S.H., MD simulations; M.-L.D.-D., H.L., L.B.R.A., B.-H.O., S.G., conception of experiments and preparation of the manuscript.

ACKNOWLEDGMENTS

We are grateful to Kim Nasmyth for critical comments on the manuscript and for suggesting names for DNA chambers in SMC. The X-ray diffraction data for *Py*SmcHd-CC80, *Py*SmcCC3, and *Bs*SmcHd(EQ)-CC30:ATPγS–ScpA^{C} were collected on the Beamline 5C at Pohang Accelerator Laboratory and on the Beamline BL5A at the Photon Factory. Diffraction data for *Bs*SmcCC1 and *Bs*SmcCC2 were obtained at DESY P11 (Hamburg, Germany) and at SLS PXII (Villigen, Switzerland), respectively. We thank all members of the Gruber and Oh labs for fruitful discussions. We are grateful to Elena Conti, her department, and the MPIB Crystallization Facility for help and advice. M.-L.D.-D. was supported by an EMBO long-term fellowship. This work was supported by the National Research Foundation of Korea (No. 2015R1A2A1A01007447 to B.-H.O.), a Samsung Science and Technology Foundation Grant (No. SSTF-BA1401-13 to S.H.), a European Research Council Starting Grant (724482 to S.G.), the Max Planck Society, and the University of Lausanne.

Received: March 2, 2017
Revised: May 3, 2017
Accepted: June 9, 2017
Published: July 6, 2017

REFERENCES

Adams, P.D., Afonine, P.V., Bunkóczi, G., Chen, V.B., Davis, I.W., Echols, N., Headd, J.J., Hung, L.W., Kapral, G.J., Grosse-Kunstleve, R.W., et al. (2010). PHENIX: a comprehensive Python-based system for macromolecular structure solution. Acta Crystallogr. D Biol. Crystallogr. *66*, 213–221.

Alt, A., Dang, H.Q., Wells, O.S., Polo, L.M., Smith, M.A., McGregor, G.A., Welte, T., Lehmann, A.R., Pearl, L.H., Murray, J.M., and Oliver, A.W. (2017). Specialized interfaces of Smc5/6 control hinge stability and DNA association. Nat. Commun. *8*, 14011.

Anderson, D.E., Losada, A., Erickson, H.P., and Hirano, T. (2002). Condensin and cohesin display different arm conformations with characteristic hinge angles. J. Cell Biol. *156*, 419–424.

Arumugam, P., Gruber, S., Tanaka, K., Haering, C.H., Mechtler, K., and Nasmyth, K. (2003). ATP hydrolysis is required for cohesin's association with chromosomes. Curr. Biol. *13*, 1941–1953.

Barysz, H., Kim, J.H., Chen, Z.A., Hudson, D.F., Rappsilber, J., Gerloff, D.L., and Earnshaw, W.C. (2015). Three-dimensional topology of the SMC2/SMC4 subcomplex from chicken condensin I revealed by cross-linking and molecular modelling. Open Biol. *5*, 150005.

Brünger, A.T., Adams, P.D., Clore, G.M., DeLano, W.L., Gros, P., Grosse-Kunstleve, R.W., Jiang, J.S., Kuszewski, J., Nilges, M., Pannu, N.S., et al. (1998). Crystallography & NMR system: a new software suite for macromolecular structure determination. Acta Crystallogr. D Biol. Crystallogr. *54*, 905–921.

Buheitel, J., and Stemmann, O. (2013). Prophase pathway-dependent removal of cohesin from human chromosomes requires opening of the Smc3-Scc1 gate. EMBO J. *32*, 666–676.

Bürmann, F., Shin, H.C., Basquin, J., Soh, Y.M., Giménez-Oya, V., Kim, Y.G., Oh, B.H., and Gruber, S. (2013). An asymmetric SMC-kleisin bridge in prokaryotic condensin. Nat. Struct. Mol. Biol. *20*, 371–379.

Bürmann, F., Basfeld, A., Vazquez Nunez, R., Diebold-Durand, M.L., Wilhelm, L., and Gruber, S. (2017). Tuned SMC arms drive chromosomal loading of prokaryotic condensin. Mol. Cell *65*, 861–872.e9.

Case, D.A., Babin, V., Berryman, J.T., Betz, R.M., Cai, Q., Cerutti, D.S., Cheatham, T.E., Darden, T.A., Duke, R.E., Gohlke, H., et al. (2014). AMBER 14 (San Francisco: University of California).

Eeftens, J.M., Katan, A.J., Kschonsak, M., Hassler, M., de Wilde, L., Dief, E.M., Haering, C.H., and Dekker, C. (2016). Condensin Smc2-Smc4 dimers are flexible and dynamic. Cell Rep. *14*, 1813–1818.

Emsley, P., Lohkamp, B., Scott, W.G., and Cowtan, K. (2010). Features and development of Coot. Acta Crystallogr. D Biol. Crystallogr. *66*, 486–501.

Gligoris, T.G., Scheinost, J.C., Bürmann, F., Petela, N., Chan, K.L., Uluocak, P., Beckouët, F., Gruber, S., Nasmyth, K., and Löwe, J. (2014). Closing the cohesin ring: structure and function of its Smc3-kleisin interface. Science *346*, 963–967.

Gruber, S., and Errington, J. (2009). Recruitment of condensin to replication origin regions by ParB/SpoOJ promotes chromosome segregation in B. subtilis. Cell *137*, 685–696.

Gruber, S., Arumugam, P., Katou, Y., Kuglitsch, D., Helmhart, W., Shirahige, K., and Nasmyth, K. (2006). Evidence that loading of cohesin onto chromosomes involves opening of its SMC hinge. Cell *127*, 523–537.

Gruber, S., Veening, J.W., Bach, J., Blettinger, M., Bramkamp, M., and Errington, J. (2014). Interlinked sister chromosomes arise in the absence of condensin during fast replication in B. subtilis. Curr. Biol. *24*, 293–298.

Haering, C.H., Löwe, J., Hochwagen, A., and Nasmyth, K. (2002). Molecular architecture of SMC proteins and the yeast cohesin complex. Mol. Cell *9*, 773–788.

Hirano, T. (2016). Condensin-based chromosome organization from bacteria to vertebrates. Cell *164*, 847–857.

Hirano, M., and Hirano, T. (2002). Hinge-mediated dimerization of SMC protein is essential for its dynamic interaction with DNA. EMBO J. *21*, 5733–5744.

Hirano, M., and Hirano, T. (2006). Opening closed arms: long-distance activation of SMC ATPase by hinge-DNA interactions. Mol. Cell *21*, 175–186.

Hirano, M., Anderson, D.E., Erickson, H.P., and Hirano, T. (2001). Bimodal activation of SMC ATPase by intra- and inter-molecular interactions. EMBO J. *20*, 3238–3250.

Hopfner, K.P. (2016). Invited review: architectures and mechanisms of ATP binding cassette proteins. Biopolymers *105*, 492–504.

Huis in 't Veld, P.J., Herzog, F., Ladurner, R., Davidson, I.F., Piric, S., Kreidl, E., Bhaskara, V., Aebersold, R., and Peters, J.M. (2014). Characterization of a DNA exit gate in the human cohesin ring. Science *346*, 968–972.

Hutchison, C.A., 3rd, Chuang, R.Y., Noskov, V.N., Assad-Garcia, N., Deerinck, T.J., Ellisman, M.H., Gill, J., Kannan, K., Karas, B.J., Ma, L., et al. (2016). Design and synthesis of a minimal bacterial genome. Science *351*, aad6253.

Jeppsson, K., Kanno, T., Shirahige, K., and Sjögren, C. (2014). The maintenance of chromosome structure: positioning and functioning of SMC complexes. Nat. Rev. Mol. Cell Biol. *15*, 601–614.

Kabsch, W. (2010). Xds. Acta Crystallogr. D Biol. Crystallogr. *66*, 125–132.

Kamada, K., Miyata, M., and Hirano, T. (2013). Molecular basis of SMC ATPase activation: role of internal structural changes of the regulatory subcomplex ScpAB. Structure *21*, 581–594.

Kamada, K., Su'etsugu, M., Takada, H., Miyata, M., and Hirano, T. (2017). Overall shapes of the SMC-ScpAB complex are determined by balance between constraint and relaxation of its structural parts. Structure *25*, 603–616.e4.

Lammens, A., Schele, A., and Hopfner, K.P. (2004). Structural biochemistry of ATP-driven dimerization and DNA-stimulated activation of SMC ATPases. Curr. Biol. *14*, 1778–1782.

Locher, K.P. (2016). Mechanistic diversity in ATP-binding cassette (ABC) transporters. Nat. Struct. Mol. Biol. *23*, 487–493.

Marbouty, M., Le Gall, A., Cattoni, D.I., Cournac, A., Koh, A., Fiche, J.B., Mozziconacci, J., Murray, H., Koszul, R., and Nollmann, M. (2015). Condensin- and replication-mediated bacterial chromosome folding and origin condensation revealed by Hi-C and super-resolutioni. Mol. Cell *59*, 588–602.

Melby, T.E., Ciampaglio, C.N., Briscoe, G., and Erickson, H.P. (1998). The symmetrical structure of structural maintenance of chromosomes (SMC) and MukB proteins: long, antiparallel coiled coils, folded at a flexible hinge. J. Cell Biol. *142*, 1595–1604.

Merkenschlager, M., and Nora, E.P. (2016). CTCF and cohesin in genome folding and transcriptional gene regulation. Annu. Rev. Genomics Hum. Genet. *17*, 17–43.

Minnen, A., Bürmann, F., Wilhelm, L., Anchimiuk, A., Diebold-Durand, M.L., and Gruber, S. (2016). Control of Smc coiled coil architecture by the ATPase heads facilitates targeting to chromosomal ParB/parS and release onto flanking DNA. Cell Rep. *14*, 2003–2016.

Park, Y.B., Hohl, M., Padjasek, M., Jeong, E., Jin, K.S., Krężel, A., Petrini, J.H., and Cho, Y. (2017). Eukaryotic Rad50 functions as a rod-shaped dimer. Nat. Struct. Mol. Biol. *24*, 248–257.

Peters, J.M., and Nishiyama, T. (2012). Sister chromatid cohesion. Cold Spring Harb. Perspect. Biol. *4*, 4.

Roversi, P., Blanc, E., Johnson, S., and Lea, S.M. (2012). Tetartohedral twinning could happen to you too. Acta Crystallogr. D Biol. Crystallogr. *68*, 418–424.

Schneider, C.A., Rasband, W.S., and Eliceiri, K.W. (2012). NIH Image to ImageJ: 25 years of image analysis. Nat. Methods *9*, 671–675.

Sheldrick, G.M. (2010). Experimental phasing with SHELXC/D/E: combining chain tracing with density modification. Acta Crystallogr. D Biol. Crystallogr. *66*, 479–485.

Soh, Y.M., Bürmann, F., Shin, H.C., Oda, T., Jin, K.S., Toseland, C.P., Kim, C., Lee, H., Kim, S.J., Kong, M.S., et al. (2015). Molecular basis for SMC rod formation and its dissolution upon DNA binding. Mol. Cell *57*, 290–303.

Stigler, J., Çamdere, G.O., Koshland, D.E., and Greene, E.C. (2016). Single-molecule imaging reveals a collapsed conformational state for DNA-bound cohesin. Cell Rep. *15*, 988–998.

Studier, F.W. (2005). Protein production by auto-induction in high density shaking cultures. Protein Expr. Purif. *41*, 207–234.

Sullivan, N.L., Marquis, K.A., and Rudner, D.Z. (2009). Recruitment of SMC by ParB-parS organizes the origin region and promotes efficient chromosome segregation. Cell *137*, 697–707.

Waldman, V.M., Stanage, T.H., Mims, A., Norden, I.S., and Oakley, M.G. (2015). Structural mapping of the coiled-coil domain of a bacterial condensin and comparative analyses across all domains of life suggest conserved features of SMC proteins. Proteins *83*, 1027–1045.

Wang, X., Tang, O.W., Riley, E.P., and Rudner, D.Z. (2014). The SMC condensin complex is required for origin segregation in Bacillus subtilis. Curr. Biol. *24*, 287–292.

Wang, X., Le, T.B., Lajoie, B.R., Dekker, J., Laub, M.T., and Rudner, D.Z. (2015). Condensin promotes the juxtaposition of DNA flanking its loading site in Bacillus subtilis. Genes Dev. *29*, 1661–1675.

Wang, X., Brandão, H.B., Le, T.B., Laub, M.T., and Rudner, D.Z. (2017). Bacillus subtilis SMC complexes juxtapose chromosome arms as they travel from origin to terminus. Science *355*, 524–527.

Weitzer, S., Lehane, C., and Uhlmann, F. (2003). A model for ATP hydrolysis-dependent binding of cohesin to DNA. Curr. Biol. *13*, 1930–1940.

Wilhelm, L., Bürmann, F., Minnen, A., Shin, H.C., Toseland, C.P., Oh, B.H., and Gruber, S. (2015). SMC condensin entraps chromosomal DNA by an ATP hydrolysis dependent loading mechanism in Bacillus subtilis. eLife *4*, 4.

Woo, J.S., Lim, J.H., Shin, H.C., Suh, M.K., Ku, B., Lee, K.H., Joo, K., Robinson, H., Lee, J., Park, S.Y., et al. (2009). Structural studies of a bacterial condensin complex reveal ATP-dependent disruption of intersubunit interactions. Cell *136*, 85–96.

Zhao, S., and Fernald, R.D. (2005). Comprehensive algorithm for quantitative real-time polymerase chain reaction. J. Comput. Biol. *12*, 1047–1064.

CellPress

STAR★METHODS

KEY RESOURCES TABLE

REAGENT or RESOURCE	SOURCE	IDENTIFIER
Antibodies		
Anti-Smc polyclonal rabbit antibody, affinity purified	Gruber Lab	COD008
Chemicals, Peptides, and Recombinant Proteins		
Adenosine 5′-[g-thio]triphosphate	Sigma-Aldrich	Cat #A1388
Benzonase	Sigma-Aldrich	Cat #A1014
Bis(maleimido)ethane (BMOE)	Thermo Scientific	Cat #22323
Breathe-Easy	Diversified Biotech	Cat #BEM-1
BsaI	New England Biolabs	Cat #R0535L
Dynabeads Protein G	Life Technologies	Cat #10004D
Erythromycin	AppliChem	Cat #A2275
GlycoBlue	Ambion	Cat #AM9515
HaloTag TMR Ligand	Promega	Cat #G825A
His-Tev protease	MPIB Core Facility	His-Tev
HisTrap 5 mL	GE Healthcare	Cat #17-5247-01
HiTrap Q HP	GE Healthcare	Cat #17-1153-01
HiLoad 16/60 S200	GE Healthcare	Cat #28989335
HiLoad 26/60 S200	GE Healthcare	Cat #28989336
HisPur Cobalt Resin	Thermo Scientific	Cat #89966
Lincomycin	AppliChem	Cat #A7697
L-Selenomethionine	Merck	Cat #561505
Phusion Hot Start II DNA Polymerase	Thermo Scientific	Cat #F-549L
Phytic acid	Sigma-Aldrich	Cat #68388
Protease Inhibitor Cocktail	Sigma-Aldrich	Cat #P8849
Ready-Lyse Lysozyme Solution	Epicenter	Cat #R1810M
RNase A	Sigma-Aldrich	Cat #R5125
SmDNase	MPIB Core Facility	SmDNase
T4 DNA ligase	Thermo Scientific	Cat #EL0016
Critical Commercial Assays		
MasterBlock 96 well, 2 mL	Greiner Bio One	Cat #780270
Takyon No ROX SYBR MasterMix blue dTTP	Eurogentec	Cat #UF-NSMT-B0702
NucleoFast 96 PCR Plate	Macherey Nagel	Cat #743100.1
NuPAGE 4%–12% Bis-Tris Gels	Life Technologies	Cat #NP0323BOX
NuPAGE 3%–8% Tris-Acetate Gels	Life Technologies	Cat #EA03755BOX
QIAquick PCR Purification Kit	QIAGEN	Cat #28106
Deposited Data		
*Bs*SmcCC2	Protein Data Bank	PDB: 5NNV
*Bs*SmcCC1	Protein Data Bank	PDB: 5NMO
*Py*SmcHd-CC80	Protein Data Bank	PDB: 5XEI
*Py*SmcCC3	Protein Data Bank	PDB: 5XG2
*Bs*SmcHd(EQ)-CC30:ATP-ScpA(C)	Protein Data Bank	PDB: 5XG3
*Pf*SmcHd-CC25-ScpA(C)	Protein Data Bank	PDB: 5XNS
Original data	Mendeley Data	http://dx.doi.org/10.17632/c2nmr3yhnp.1
Experimental Models: Organisms/Strains		
E. coli and *B. subtilis* strains, see Table S7	Gruber Lab	N/A

(*Continued on next page*)

Continued		
REAGENT or RESOURCE	SOURCE	IDENTIFIER
Oligonucleotides		
qPCR primers, see Table S3	Gruber Lab	N/A
PCR primers for HTP cysteine cross-linking screen, see Table S4	This paper	N/A
Recombinant DNA		
Plasmid DNA, see Table S8	Gruber Lab	N/A
Software and Algorithms		
SHELX	Sheldrick, 2010	http://shelx.uni-ac.gwdg.de/SHELX/
XDS	Kabsch, 2010	http://xds.mpimf-heidelberg.mpg.de/
Phenix	Adams et al., 2010	https://www.phenix-online.org/
CNS	Brünger et al., 1998	http://structure.usc.edu/cns/about_cns/frame.html
Coot	Emsley et al., 2010	https://www2.mrc-lmb.cam.ac.uk/personal/pemsley/coot/
AMBER 14	Case et al., 2014	http://ambermd.org/
ImageJ	Schneider et al., 2012	https://imagej.nih.gov/ij/
Real Time PCR Miner	Zhao and Fernald, 2005	http://ewindup.info/miner/
Other		
pRSFDuet-CPD	Oh Lab	N/A
pJK-CPD	Oh Lab	N/A

CONTACT FOR REAGENT AND RESOURCE SHARING

Further information and requests for reagents may be directed to, and will be fulfilled by the Lead Contact, Stephan Gruber (stephan.gruber@unil.ch).

EXPERIMENTAL MODEL AND SUBJECT DETAILS

Bacillus subtilis Strains and Growth

B. subtilis strains are derived from the isolate 1A700 (BGSC, Bacillus Genetic Stock Centre). Strain usage for the reported experiments is listed in Table S1. Transformation of naturally competent *B. subtilis* cells was performed following a 2-step starvation protocol previously described (Bürmann et al., 2013) with extended growth and starvation periods for high-efficiency transformation of *smc* mutant strains. Cells were grown overnight in 10 mL competence medium composed of SMM solution (15 mM ammonium sulfate, 80 mM dipotassium hydrogen phosphate, 44 mM potassium dihydrogen phosphate, 3.4 mM trisodium citrate, 0.8 mM magnesium sulfate 6 g l^{-1} potassium hydrogen phosphate) supplemented with 5 g l^{-1} glucose, 20 mg l^{-1} tryptophan, 20 mg l^{-1} casamino acids, 6 mM magnesium sulfate and 110 mg l^{-1} ferric ammonium citrate. 600 µL were diluted into 10 mL fresh competence medium for 5 hr at 37°C. 10 mL of prewarmed starvation medium (SMM solution supplemented with 5 g l^{-1} glucose and 6 mM magnesium sulfate) was added. After 2 hr at 37°C, cells (100 µl) were mixed with DNA and incubated for 3 hr at 37°C in a 96-well plate. Transformants were selected by plating on Oxoid nutrient agar (ONA) or SMG agar supplemented with 0.4 mg ml^{-1} erythromycin and 10 mg ml^{-1} lincomycin.

METHOD DETAILS

Protein Turnover Measurements

His-tagged versions of *Bs*SmcH-CC8 and *Bs*SmcH-CC300 proteins harboring single or double cysteines for cross-linking were purified by metal-affinity chromatography and gel filtration essentially as described previously for corresponding constructs lacking cross-linking cysteines (Soh et al., 2015). However, *E. coli* cell extracts were prepared by a swing mill rather than sonication. Equal volumes of protein solution (at equal concentration in 50 mM Tris-HCl pH 7.4, 200 mM NaCl, 1 mM TCEP) were mixed on ice and then incubated at 37°C. Aliquots were taken at the indicated time points and mixed on ice with BMOE to a final concentration of 200 µM. After 1 min, the cross-linking reaction was quenched by the addition of 24 mM 2-Mercaptoethanol and proteins denatured by heating in SDS loading buffer. Samples were analyzed by SDS-PAGE and on an Agilent 2100 Bioanalyzer using the Protein 230 Chip kit per manufacturer's instruction.

High-Throughput Allelic Replacement Screening

Smc(Cys) mutations were generated in high-throughput essentially as described for Smc gene truncations (Bürmann et al., 2017). PCR primers were designed for Smc coiled coil residues except for those predicted to occupy heptad positions ‘a’ and ‘d’ (Table S4). PCR reactions for 5′- and 3′-regions of the *smc* gene were performed in 96-well plates using Phusion Hot Start II DNA Polymerase. DNA was purified in NucleoFast 96 PCR plates and quantified using Quant-iT fluorescence spectroscopy. Circular targeting constructs were assembled in Golden Gate reactions using BsaI and T4 DNA ligase with cloned and sequence verified modules for the downstream ftsY gene (pSG849), an ermB marker cassette (pSG682), a downstream homology region (pSG841), and a non-replicating plasmid backbone containing a mazF toxin gene (pSG1525) (Figure S2A). The mazF gene was used to efficiently counter-select single-crossover integration. Reaction mixtures were transformed into a *smc* deletion strain (BSG1919). Transformants were selected on Oxoid nutrient agar (ONA) with 0.4 mg/mL erythromycin and 10 mg/mL lincomycin at 37°C. Plates were transferred to 4°C 36 hr after transformation and a single colony was randomly picked for cysteine cross-linking as described below. Please note that the strains obtained by HTP engineering were not confirmed by sequencing. Most clones were successfully regrown after extensive periods of incubation on agar plates at 4°C and subsequently stored as glycerol stocks (Table S4).

In Vivo Cysteine Cross-Linking

Cross-linking experiments were performed in cells grown in liquid SMG medium as described previously (Bürmann et al., 2013; Soh et al., 2015). For HTP cysteine cross-linking the protocol was adapted for growth of cells in liquid LB Miller medium. Briefly, cells bearing the cysteine mutation and a Smc-HaloTag fusion were grown to mid-exponential phase (OD_{600} of 0.4) in a 96-deep well plates, harvested by centrifugation and resuspended in ice-cold PBS with 0.1% glycerol (PBSG). Thiol-reactive cross-linker bismaleimidoethane (BMOE) was added to a final concentration of 0.5 mM from a 20 mM stock in DMSO followed by an incubation of 10 min on ice. Any remains of the cross-linking agent were quenched by the addition of 2-mercarptoethanol (2-ME) to a final concentration of 14 mM. Samples were incubated on ice for 10 min. Cell suspensions were incubated for 15 min at 37°C in PBSG buffer containing protease inhibitor cocktail, benzonase or SmDNase, Ready-Lyse lysozyme solution and 15 μM HaloTag-TMR substrate. Samples were heated to 95°C for 5 min in LDS Sample buffer, loaded onto Tris-Acetate gels (3%–8% Novex) and run for 2.5 hr at 35 mA per gel at 4°C. Gels were scanned on a Typhoon FLA-9500 scanner (GE healthcare) with Cy3 DIGE filter setup. Bands intensities were quantified in ImageJ with background correction.

Protein Production

Bs*SmcCC1 and* Bs*SmcCC2*

The *Bs*SmcCC1 (*Bs* Smc (188-253)/SGGSGGS/(922-1011)) and *Bs*SmcCC2 fragments (*Bs* Smc (246-379)/SGGSGGS/(793-929)) were cloned in pET-22b vector with an N-terminal His6 purification tag followed by a TEV protease cleavage site. Proteins were expressed in *E.coli* BL21[DE3] cells using an auto-induction medium for native proteins (Studier, 2005) and minimal medium for selenomethionylated proteins.

The purification protocol is similar for both constructions. Cells were resuspended in a lysis buffer (200mM NaCl, 50mM Tris pH 7.4, 5mM Imidazole) and lysed by sonication. After high speed centrifugation (40000 g, 1h), soluble fraction was injected on a His-Trap column (GE Healthcare). After extensive washes with lysis buffer, the protein was eluted using an elution buffer (200mM NaCl, 50mM Tris pH 7.4, 250mM Imidazole). After concentration of the protein and buffer exchange on a MonoQ column (GE Healthcare), TEV protease was added. Uncut protein was removed by application of the sample on a HisTrap column and injected on a HiLoad 16/60 Superdex 200 gel filtration column equilibrated in a buffer containing 200mM NaCl and 25mM Tris pH 7.4. Selected fractions were pooled and concentrated on Vivaspin Turbo 15 (Sartorius).

Py*SmcHd-CC80*

*Py*SmcHd-CC80 (residues 1-254, 907-1177 and a SGGS linker) was expressed in the *E. coli* BL21(DE3) RIPL strain (Novagen) as a fusion protein connected to a cysteinyl protease domain (CPD)-10xHis tag at the C terminus. Bacterial cell lysates were prepared by sonication in Buffer A (20 mM Tris-HCl pH 7.5, 50 mM NaCl and 1 mM NaN_3) containing 5 mM β-mercaptoethanol (β-ME). The supernatant was applied to a gravity flow column filled with HisPur cobalt resin (Thermo). The CPD-His_{10} tag was removed by on-gel digestion by addition of 0.1 mM phytate, and *Py*SmcHd-CC80 was eluted from the column with Buffer A. The protein was further purified with a HiTrap Q anion exchange column (GE Healthcare) and a HiLoad 26/60 Superdex 200 gel filtration column (GE Healthcare), equilibrated with Buffer A containing 1 mM DTT.

Py*SmcCC3*

*Py*SmcCC3 (residues 345-468, 694-814 and a SGGS linker) was expressed in the *E. coli* BL21(DE3) RIPL strain (Novagen) as a fusion protein connected to a CPD-10xHis tag at the C terminus. The protein was purified in the same manner as used for *Py*SmcHd-CC80.

Bs*SmcHd(EQ)-CC30:ATP–ScpA*^{C}

*Bs*SmcHd(EQ)-CC30 (residues 1-219/SGGS linker/975-1186, E1118Q mutation) and *Bs*ScpA^C (residues 167-251) were co-expressed in the pRSFDuet CPD-10xHis plasmid in the *E. coli* BL21 (DE3) strain at 18°C. From this vector, *Bs*SmcHd(EQ)-CC30 was expressed as a fusion protein with a CPD-10xHis tag at the C terminus. Cell lysates were prepared by sonication in buffer B composed of 20 mM Tris-HCl pH 7.5 and 100 mM NaCl. The *Bs*SmcHd(EQ)-CC30–ScpA^C heterodimer was purified by using HisPur Cobalt resin (Thermo Scientific) and a HiTrap Q anion exchange column (GE Healthcare) operated with a linear NaCl gradient (50-500 mM) in buffer B. Purified complex was incubated with 5 mM $MgCl_2$ and 2 mM ATPγS tetralithium salt (Li ATPγS) (Sigma-Aldrich)

in Buffer B at 37°C for 30 min to induce dimerization of *Bs*SmcHd(EQ)-CC30–ScpA^C. This sample was loaded on a HiLoad Superdex 200 gel filtration column (GE Healthcare) and the complex was eluted with Buffer B containing additional 5 mM $MgCl_2$. The purified complex was concentrated to 20 mg/ml and treated with 2 mM Li ATPγS.

Crystallization, X-ray Data Collection, and Structure Determination

Crystallographic data statistics are summarized in Table S2.

*Bs*SmcCC1 and *Bs*SmcCC2

The *Bs*SmcCC2 (25mg/mL) crystals grew at 4°C in a condition containing 8%–14% PEG 3350, 50mM Tris pH8, 4% MPD and 200mM Calcium Acetate and the *Bs*SmcCC1 (26 mg/mL) crystals grew at 10°C in a condition containing 27%–30% PEG 3350, 50mM Tris pH8, 6%–10% MPD and 160-200mM Ammonium Acetate. In both cases the hanging-drop vapor diffusion technic was used. The crystals were flash frozen in the crystallization solution supplemented with 30% Ethylene Glycol. SAD datasets were collected at 3.0Å resolution for *Bs*SmcCC2 at SLS PXII (Villigen, Switzerland) and for *Bs*SmcCC1 at 1.9Å for native dataset and 2.4 Å for SeMet dataset at DESY P11 (Hamburg, Germany) (Table S1). Data were processed with XDS (Kabsch, 2010). *Bs*SmcCC2 crystallizes in P1 space group with tetartohedral twinning (Roversi et al., 2012). Heavy atoms sites and initial density map were calculated using SHELX (Sheldrick, 2010). Automatic building was performed using PHENIX *autobuild* (Adams et al., 2010). For *Bs*SmcCC1, the initial model calculated with SeMet dataset was used as a search model for molecular replacement in the native dataset. The models were manually reconstructed using Coot (Emsley et al., 2010) and further refined using PHENIX *refine* with twinning operators (-h, k, -l) for the *Bs*SmcCC2 structure (Adams et al., 2010).

*Py*SmcHd-CC80

Crystals of *Py*SmcHd-CC80 grew in a precipitant solution containing 4% Tacsimate buffer pH 4.0 (Hampton) and 7% PEG3350 (Hampton) at 22°C. Crystals were soaked briefly in a cryo-protectant containing 70% Tacsimate buffer pH 4.0 and flash cooled in liquid nitrogen before crystal mounting. The structure of *Py*SmcHd-CC80 was determined by molecular replacement using the structure of *Pf*Smc-CC25–ScpA^C (PDB: 5XNS) as a search model with the program PHENIX (Adams et al., 2010). The final refined model does not include *Py* Smc residues 239-254, 907-916, 939-940 and 1164-1169, whose electron densities were missing or too weak.

*Py*SmcCC3

*Py*SmcCC3 (24 mg/ml) crystallized in a precipitant solution containing 0.18 M HEPES (pH 7.0) and 49% (+/−)-2-Methyl-2,4-pentanediol (MPD) at 20°C. Crystals were flash cooled in liquid nitrogen before crystal mounting. The crystal contained one molecule of *Py*SmcCC3 in the asymmetric unit. The crystal structure of *Py*SmcCC3 was determined by molecular replacement using the polyalanine-substituted coordinate of the coiled coil portion (residues 446-498, 663-712) of *Pf* Smc hinge dimer (Soh et al., 2015) as a search model with the program PHENIX (Adams et al., 2010).

*Bs*SmcHd(EQ)-CC30:ATPγS–ScpA^C

Crystals of the *Bs*SmcHd(EQ)-CC30:ATPγS–ScpA^C complex grew in a precipitant solution containing 10% PEG3000, 0.1 M imidazole pH 8.0, 0.2 M Li_2SO_4, and 0.05 M hexamine cobalt (III) chloride. The asymmetric unit of the crystal contained one molecule of dimerized *Bs*SmcHd(EQ)-CC30:ATPγS–ScpA^C. The structure of the complex was determined by molecular replacement using the structure of the head domain of *Pf* Smc (PDB entry: 1XEX) (Lammens et al., 2004). The final refined model does not include *Bs*Smc residues 16, 50-56, 60, 110, 130-133, 136, 204-219, 975-990, 1066-1067, 1080, 1084 and 1178-1186 in chain A, 25, 51-57, 133, 136, 198-219, 975-989, 994, 1066, 1069 and 1178-1186 in chain B and *Bs*ScpA^C residues 167-175, 208-210, 246-251 in chain C, 167-178, 191-197, 207-210, 229, 242-251 in chain D. The electron densities for these residues were missing or too weak. X-ray data were collected on either the Beamline 5C at the Pohang Accelerator Laboratory or the BL5A at the Photon Factory. Iterative model building and structure refinement were performed using the program *CNS* (Brünger et al., 1998).

Energy Minimization Calculation for the Ring-Shaped *Pf* Smc Dimer

We performed energy minimization for the open ring form of the *Pf* Smc dimer in gas phase by using SANDER module of AMBER 14 package with the FF14SB protein force field. The system was energy-minimized for 5,000 steps of the steepest descent minimization to remove strains. The non-bonded interactions were calculated by using the cutoff value of 12 Å.

Chromatin Immunoprecipitation

ChIP-qPCR was performed essentially as described in (Minnen et al., 2016) and (Bürmann et al., 2017). Cultures of 400 mL SMG were inoculated to OD_{600} = 0.004 and grown to OD_{600} = 0.02 at 37°C. Cells were fixed by addition of 40 mL of buffer F (50 mM Tris-HCl pH 7.4/24°C, 100 mM NaCl, 0.5 mM EGTA pH 8.0/24°C, 1 mM EDTA pH 8.0/24°C, 10% Formaldehyde) and incubation for 30 min at room temperature. Cells were harvested by filtration and washed in PBS. Cells were re-suspended in 1 mL TSEMS (50 mM Tris pH 7.4/24°C, 50 mM NaCl, 10 mM EDTA pH 8.0/24°C, 0.5 M sucrose, protease inhibitor cocktail) containing 20 mg/mL lysozyme. Protoplasting was done by shaking at 37°C for 30 min. Protoplasts were washed once in 2 mL TSEMS, re-suspended in TSEMS, split into aliquots corresponding to 1ml at OD_{600} = 2 and pelleted. Pellets were frozen in liquid nitrogen and stored at −80°C.

Pellets were re-suspended in 2 mL buffer L (50 mM HEPES-KOH pH 7.5/24°C, 140 mM NaCl, 1 mM EDTA pH 8.0/24°C, 1% Triton X-100, 0.1% Na-deoxycholate) containing 0.1 mg/mL RNase A and protease inhibitor cocktail and transferred into 5 mL round bottom tubes. The suspension was sonicated 3x for 20 s on a Bandelin Sonoplus sonicator using a MS-72 tip with 90% pulse time and 35% power output. The extract was centrifuged at 4° and 20,000 × g and 200 μL were kept as input reference.

For immunoprecipitation, 800 μL of the extract were loaded on 50 μL Dynabeads Protein-G charged with 50 μL Anti-Smc antiserum and incubated for 2 hr on a rotating wheel at 4°C. Beads were washed at room temperature in 1 mL each of buffer L, buffer L5 (buffer L containing 500 mM NaCl) and buffer W (10 mM Tris-HCl pH 8.0/24°C, 250 LiCl, 0.5% NP-40, 0.5% Na-Deoxycholate, 1 mM EDTA pH 8.0/24°C). Beads were resuspended in 520 μL buffer TES (50 mM Tris-HCl pH 8.0/24°C, 10 mM EDTA pH 8.0/24°C, 1% SDS). The reference sample was mixed with 300 μL buffer TES and 20 μL 10% SDS. Cross-links were reversed over-night at 65°C with shaking.

For phenol/chloroform extraction, 500 μL samples and reference samples were cooled to room temperature, vigorously mixed with 500 μL phenol equilibrated with buffer (10 mM Tris-HCl pH 8.0, 1 mM EDTA) and centrifuged for 10 min at 20,000 × g. Then, 450 μL of the supernatant was vigorously mixed with 450 μL chloroform and centrifuged for 10 min at 20,000 × g. For DNA precipitation, 400 μL of the supernatant were mixed with 3 μL GlycoBlue, 40 μL of 3 M Na-Acetate pH 5.2/24°C and 1 mL ethanol and incubated for 20 min at −20°C. Samples were centrifuged at 4°C and 20,000 × g for 10 min, and the precipitate was dissolved in 150 μL TE buffer (10mM Tris-HCl pH8.0, 1mM EDTA pH8.0) for 15 min at 55°C, purified with a PCR purification kit, and eluted in 30 μL buffer EB.

For qPCR, samples were diluted in water (1:10 for IP and 1:500 for input), and duplicate 10 μL reactions (5 μL master mix, 1 μL of 3 μM primer mix, 4 μL sample) were run in a Rotor-Gene Q device using qPCR MasterMix using primer pairs described in Bürmann et al., 2017 and listed in Table S3.

Colony Formation Assay

Cells were pre-grown in a 96-well plates in SMG medium for 24 hr at 37°C (Minnen et al., 2016). Overnight cultures were diluted 81-fold (high density spots) and 59049-fold (low density spots) and spotted onto ONA (5 μL per spot) and SMG (7.5 μL per spot) agar plates. Plates were incubated at 37°C for 14 hr on ONA or 24 hr on SMG.

QUANTIFICATION AND STATISTICAL ANALYSIS

Analysis of Cross-Linking Efficiencies

HaloTag-TMR and HaloTag-OG in-gel fluorescence was quantified with ImageJ 1.49v software using the built-in gel-analyzer function (Schneider et al., 2012). Briefly, relative band intensities (fraction of cross-linked protein) were calculated using a graphical method that involves generating lane profile plots, manually delineating peaks of interest, and then integrating peak areas. Two biological replicates (with three technical replicates each) were used to calculate mean and standard deviation reported in the corresponding figures and tables (except for the HTP screen (Figure 2A) where single measurements are reported).

Analysis of Chromatin-Immunoprecipitation Efficiencies

C_T values were extracted from qPCR curves by automatic fitting using the online software *Real-time PCR miner* (Zhao and Fernald, 2005). ChIP/input ratios were calculated as $\alpha\ 2^{\Delta CT}$, where $\Delta C_T = C_T$ (Input) – C_T (ChIP) and α is given by the extraction volumes and sample dilutions. All data are derived as mean of duplicate qPCR reactions. Means and standard deviations for ChIP efficiencies were calculated from three biological replicates (Figure 6F).

DATA AND SOFTWARE AVAILABILITY

The coordinates of the structures together with the structure factors are deposited at the Protein Data Bank. Accession codes are available in the Key Resources Table. Original data is available at Mendeley: http://dx.doi.org/10.17632/c2nmr3yhnp.1.

Molecular Cell, Volume *67*

Supplemental Information

Structure of Full-Length SMC and Rearrangements Required for Chromosome Organization

Marie-Laure Diebold-Durand, Hansol Lee, Laura B. Ruiz Avila, Haemin Noh, Ho-Chul Shin, Haeri Im, Florian P. Bock, Frank Bürmann, Alexandre Durand, Alrun Basfeld, Sihyun Ham, Jérôme Basquin, Byung-Ha Oh, and Stephan Gruber

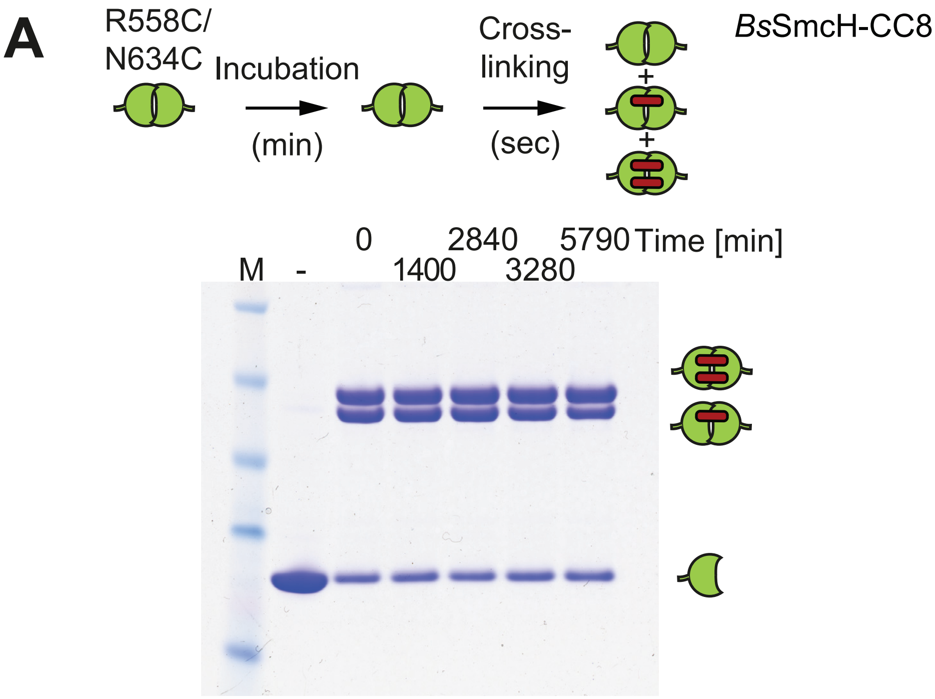
A
R558C/
N634C
Incubation
(min)
Cross-
linking
(sec)
BsSmcH-CC8
0
2840
5790
Time [min]
M
-
1400
3280

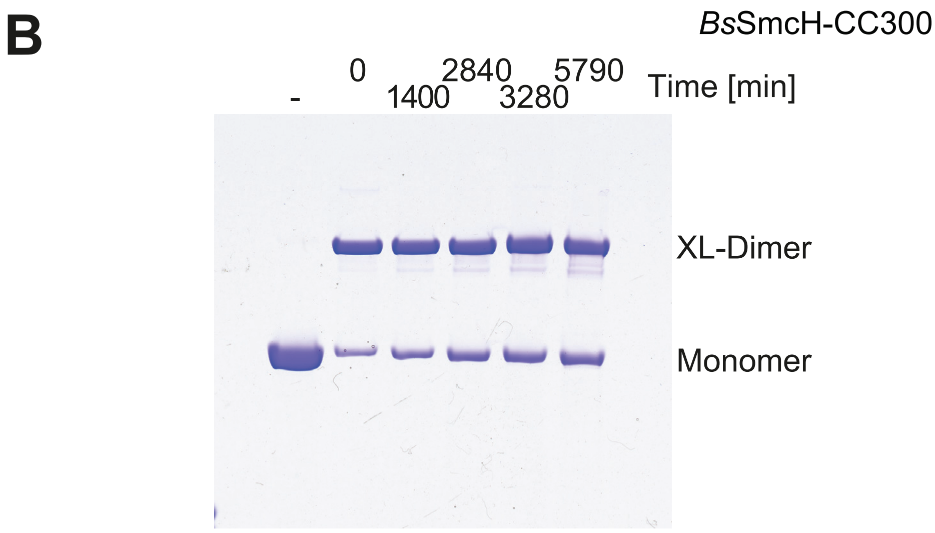
B
BsSmcH-CC300
0
2840
5790
Time [min]
-
1400
3280
XL-Dimer
Monomer

Figure S1

Figure S1. Robust long-term stability of cysteine cross-linking. Related to Figure 1.

(A) Purified preparations of *Bs*SmcH-CC8(R558C, N634C) were incubated at 37°C. At the indicated time points samples were cross-linked by BMOE. Monomer and cross-linked dimer species were separated by SDS-PAGE and detected by Coomassie staining. ´-´ denotes samples treated with DMSO instead of BMOE.

(B) Same as in (A) with *Bs*SmcH-CC300(R558C, N634C). Please note that robust cysteine cross-linking is observed after extended periods of incubation at 37°C. The lack of cross-linking observed in Figure 1C is thus due to slow subunit turn-over.

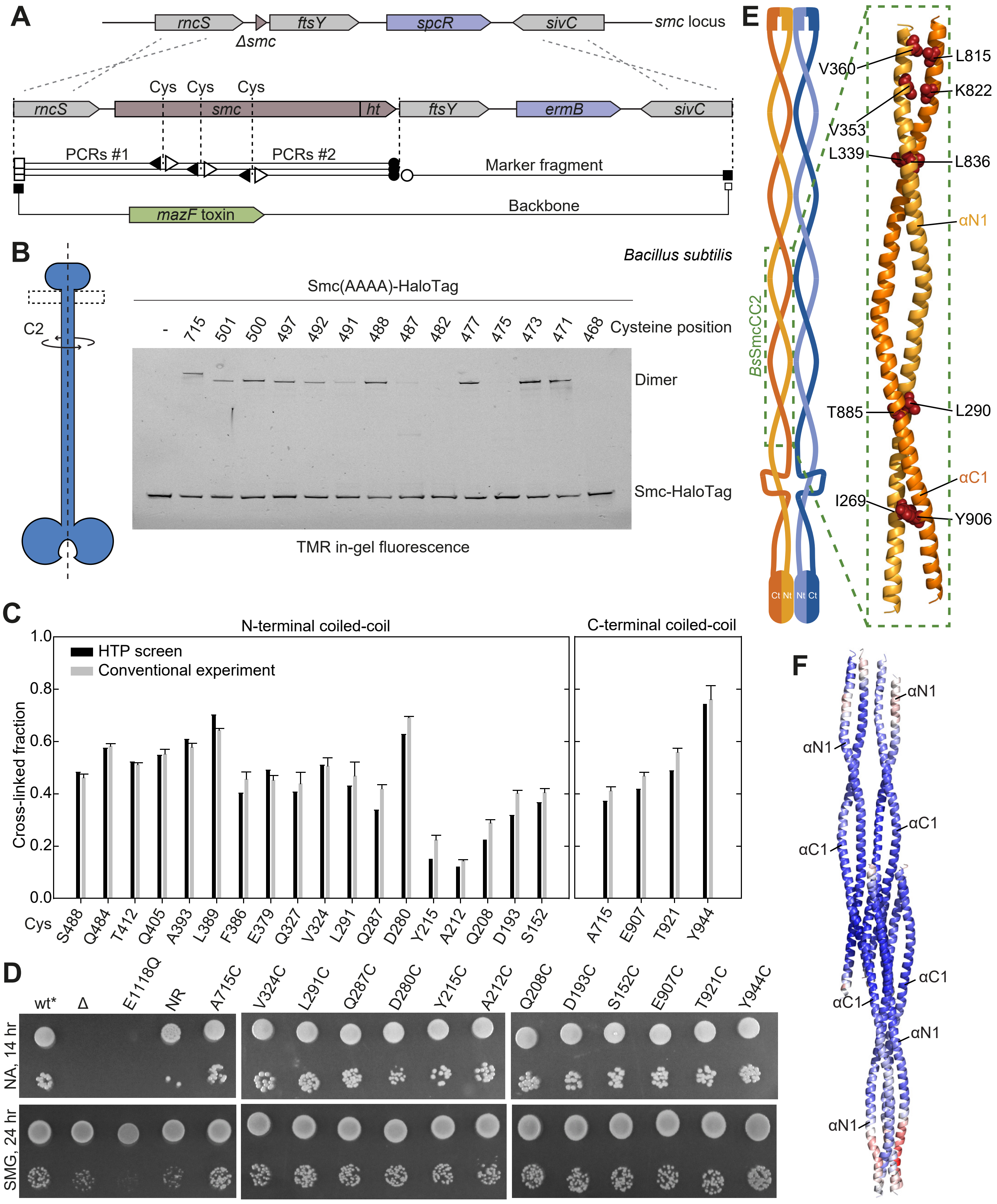
A
rncS
ftsY
spcR
sivC
smc locus
Δsmc
Cys Cys Cys
rncS
smc
ht
ftsY
ermB
sivC
PCRs #1
PCRs #2
Marker fragment
mazF toxin
Backbone
B
C2
Bacillus subtilis
Smc(AAAA)-HaloTag
- 715 501 500 497 492 491 488 487 482 477 475 473 471 468
Cysteine position
Dimer
Smc-HaloTag
TMR in-gel fluorescence
C
N-terminal coiled-coil
C-terminal coiled-coil
HTP screen
Conventional experiment
Cross-linked fraction
Cys
S488 Q484 T412 Q405 A393 L389 F386 E379 Q327 V324 L291 Q287 D280 Y215 A212 Q208 D193 S152
A715 E907 T921 Y944
D
wt* Δ E1118Q NR A715C V324C L291C Q287C D280C Y215C A212C Q208C D193C S152C E907C T921C Y944C
NA, 14 hr
SMG, 24 hr
E
BsSmcCC2
V360 L815
K822
V353
L339 L836
αN1
T885 L290
αC1
I269 Y906
Ct Nt Nt Ct
F
αN1
αN1
αC1
αC1
αC1
αC1
αN1
αN1

Figure S2

Figure S2. Mapping of the Smc rod by cysteine cross-linking and structural analysis.

Related to Figure 2.

(A) Gene targeting strategy.

(B) Exemplary output of cysteine mapping screen for Smc residues located close to the Smc hinge domain in the N-terminal α-helix. Smc-HaloTag species labelled by HaloTag-TMR are separated by SDS-PAGE and detected by in-gel fluorescence scanning. Smc(A715C) serves as a positive control (Soh et al., 2015). Please note that the migration of the cross-linked dimer fraction depends on the position of the cysteine residue. All original gel images are available on Mendeley Data.

(C) Selected Smc(Cys) mutants were generated by conventional allelic replacement without selection for Smc function during growth. Cysteine cross-linking efficiency was compared to corresponding results from the high-throughput screen ('HTP'). Mean and standard deviation from three technical replicates are shown for the conventional experiment.

(D) Growth of selected Smc(Cys) mutants on nutrient rich ('Oxoid nutrient agar', 'ONA') and nutrient poor medium ('SMG'). Serial dilutions of overnight-cultures were spotted. Strain denoted as 'NR' is not related to this study.

(E) Organization of the Smc coiled coil in *Bs*SmcCC2 in cartoon representation. N- and the C-terminal helices (residues 246-379 and 793-929, respectively) are colored in bright and dark orange colors, respectively. Previously identified heptad residues (positions 'a' and 'd') are displayed as spheres in red colors (Minnen et al., 2016; Waldman et al., 2015).

(F) Organization of the two dimers of BsSmcCC2 in the crystal unit. Molecules are colored according to the local B-factor, lowest in blue, highest in red. The two dimers are organized top-to-tail in the asymmetric unit, the head proximal region of the coiled coils are juxtaposed. Probably due to crystal packing, the electron density of the head-proximal region is better defined, the main- and side-chains being clearly visible in the density.

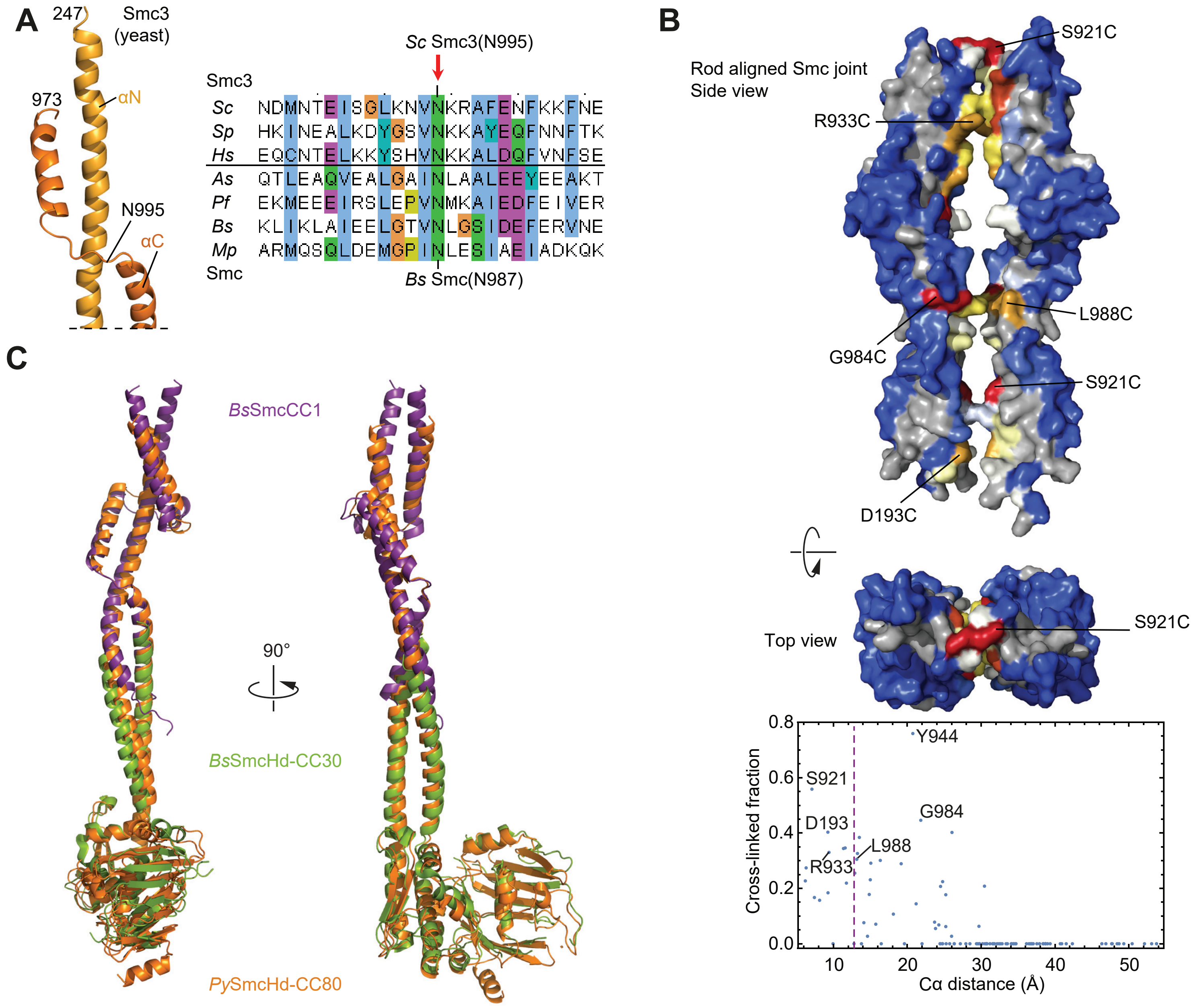

D

```
-MFLKRLDVIGFKSFAER-ISVDFVKGVTAVVGPNGSGKSNITDAIRWVLGEQSARSLRG Bs
MPYIEKLELKGFKSYGNRKVVIPFSKGFTAIVGANGSGKSNIGDAILFVLGGLSAKAMRA Py
1 ::::*:: ****:.:* : : * **.**:** ********* *** :***  **::::*.

59
GKMEDIIFAGSDSRKRLNLAEVTLTLDNDDHFLPIDFHEVSVTRRVYRSGESEFLINNQP Bs
TRISDLIFAGSKGEPPAKYAEVAIYFNNEDRGFPIDEDEVVIKRRVYPDGRSAYWLNGRR Py
 ::.*:*****...   : ***:: ::*:*: :*** .** :.**** .*.* : :*.:

119                            S152
CRLKDIIDLFMDSGLGKEAFSIISQGKVEEILSSKAEDRRSIFEEAAGVLKYKTRKKKAE Bs
ATRSEILDLLSAAMISPEGYNLVLQGDITKFIKMSPLERRQLIDEISGIAEYDAKKEKAL Py
.  .:*:**:  : :. *.:.:: **.: :::. .   :**.:::* :*: :*.::*:**
                                 M154

179         D193
NKLFETQDNLNRVEDILHELEGQVEPLKIQASIAKDYLEKKKELEHVEIALTAYDIEELH Bs
EELKQAEENLARVDLLIKEVKKQLDKLEKERNDALRYLDLKERLERARVALLLGEIKRLE Py
::* ::::** **: :::*:: *:: *: : . *  **: *:.**:..:**   :*:.*.
            L195

239
GKWSTLK Bs
SMIDEGE Py
241 .  :

928          Y944
EVKLGRMEVELDNLLQYLREEYSLSFEGAKEKYQLETDPEEARKRVKLIK Bs
KVKSAQLRIQLEEKRRELKH-FDAAL--IRSVKEVSLDLEVLRKEIEDME Py
:** .::.::*::  : *:. :. ::    :.  ::. * *  **.:: ::
911          E927

978
LAIEELGTVNLGSIDEFERVNERYKFLSEQKEDLTEAKNTLFQVIEEMDEEMTKRFNDTF Bs
AEIKALEPVNMKAIEDFEVVERRYLELKSKREKLEAEKESIIEFINEIEKEKKNVFMRTL Py
  *: *  **: :*::** *:.**  *..::*.*   *:::::.*:*:::* .: *  *:

1038
VQIRSHFDQVFRSLFGGGRAELRLTDPNDLLHSGVEIIAQPPGKKLQNLNLLSGGERALT Bs
EAIAKNFSELFAKLSPGGSARLILENPEDPFSGGLEIEAKPAGKDVKRIEAMSGGEKALT Py
  * .:*.::* .*  ** *.* * :*:* : .*:** *:* **.::.:: :****:***

1098                                                   K1151
AIALLFSILKVRPVPFCVLDEVEAALDEANVFRFAQYLKKYSSDTQFIVITHRKGTMEEA Bs
ALAFIFAIQRFKPAPFYLFDEIDAHLDDANVKRVADLIKESSRESQFIVITLRDVMMANA Py
*:*::*:* :.:*.** ::**::* **:*** *.*: :*: * ::****** *.   * :*
                                                       D1131

1158
DVLYGVTMQESGVSKVISVKLEETKEFVQ------------ Bs
DKIIGVSMRD-GVSRVVSLSLEKAMKILEEAKKRNEATIGG Py
*  : **:*:: ***:*:*:.**:: ::::
```

Figure S3

Figure S3. The Smc joint domain in *Bs* Smc and *Py* Smc. Related to Figure 3.

(A) Structure of part of the Smc3 joint domain present in PDB: 4UX3 (left panel). Sequence alignment of C-terminal SMC joint residues indicating highly-conserved residue including an invariable asparagine.

(B) Two monomers of *Bs*SmcCC1 (in side view and top view) were arranged side-by-side in a manner that minimizes the overall distances between cross-linkable cysteine side chains in the Smc joint domain and the adjacent coiled coil. Rendering, color coding and distance/cross-linking efficiency graph as in Figure 2B.

(C) Superimposition of *Py*SmcHd-CC80 (in orange colors) with *Bs*SmcCC1 (in purple colors) and the Smc moiety of *Bs*SmcHd-CC30:ATPγS–ScpA^{C} (in green colors). Two side views are displayed in the left and right panels. Overall, the structures display excellent overlap.

(D) *Bs* Smc residues corresponding to residues in *Py*SmcHd-CC80. N-terminal Smc sequences (left panel) and C-terminal Smc sequences (right panel) are displayed. Numbers above and below the sequence lines denote *Bs* and *Py* Smc positions, respectively. Selected residues are highlighted by red boxes.

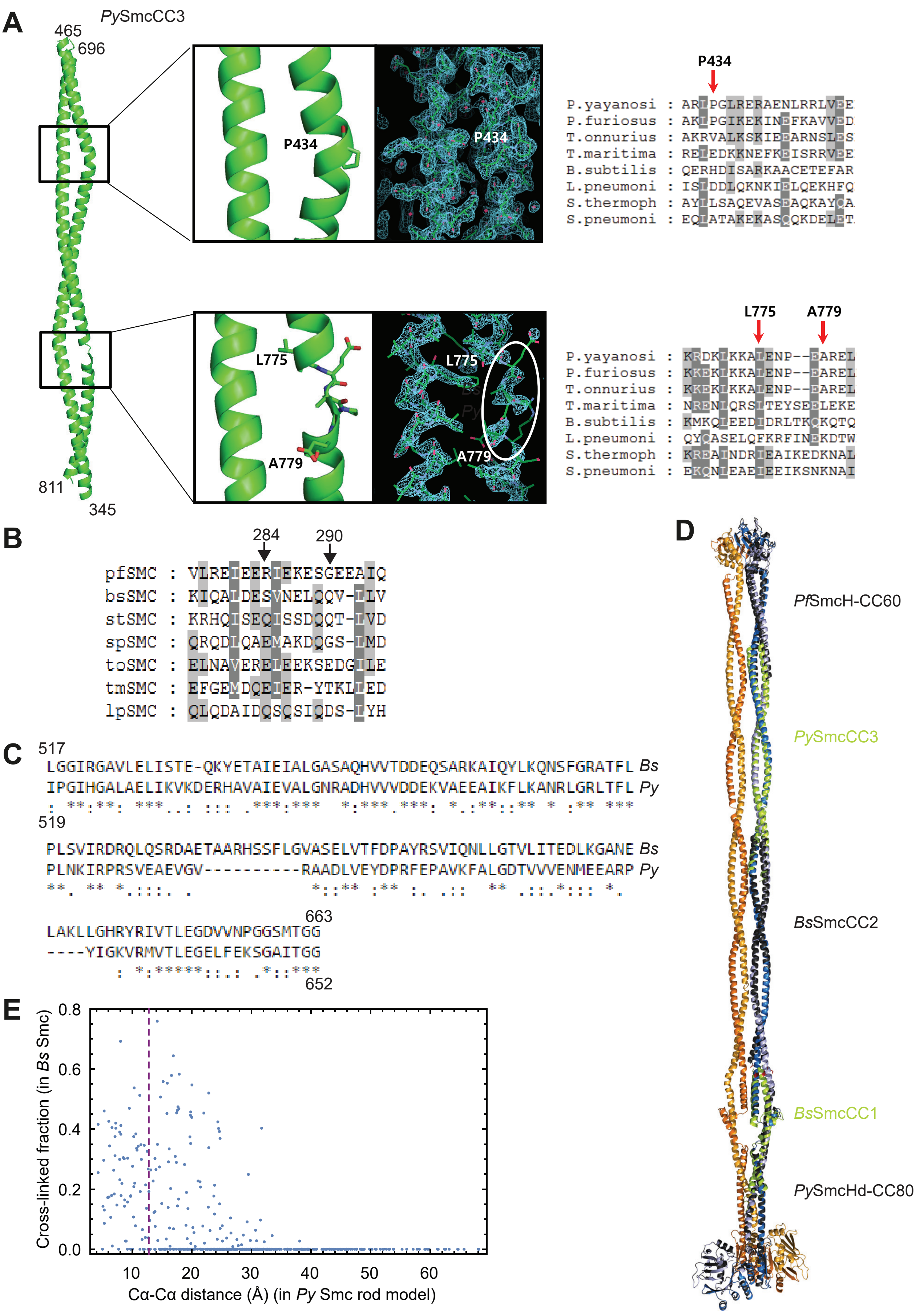
A
PySmcCC3
465
696
P434
811
345
L775
A779
P.yayanosi : ARIPGLRERAENLRRLVEE
P.furiosus : AKIPGIKEKINEFKAVVED
T.onnurius : AKRVALKSKIEEARNSLES
T.maritima : RELEDKKNEFKEISRRVEE
B.subtilis : QERHDISARKAACETEFAR
L.pneumoni : ISIDDLQKNKIELQEKHFQ
S.thermoph : AYILSAQEVASEAQKAYCA
S.pneumoni : EQIATAKEKASQQKDELET
P.yayanosi : KRDKIKKAIENP--EAREL
P.furiosus : KKEKIKKAIENP--EAREL
T.onnurius : KKEKIKKAIENP--EAREL
T.maritima : NRENIQRSITEYSEELEKE
B.subtilis : KMKQIEEDIDRLTKCKQTQ
L.pneumoni : QYCASELQFKRFINEKDTW
S.thermoph : KREAINDRIEAIKEDKNAL
S.pneumoni : EKQNIEAEIEEIKSNKNAI
B
284
290
pfSMC : VLREIEERIEKESGEEAIQ
bsSMC : KIQAIDESVNELQQV-ILV
stSMC : KRHQISEQISSDQQT-IVD
spSMC : QRQDIQAEMAKDQGS-IMD
toSMC : ELNAVEREIEEKSEDGILE
tmSMC : EFGEMDQEIER-YTKLIED
lpSMC : QLQDAIDQSQSIQDS-IYH
C
517
LGGIRGAVLELISTE-QKYETAIEIALGASAQHVVTDDEQSARKAIQYLKQNSFGRATFL Bs
IPGIHGALAELIKVKDERHAVAIEVALGNRADHVVVDDEKVAEEAIKFLKANRLGRLTFL Py
519
PLSVIRDRQLQSRDAETAARHSSFLGVASELVTFDPAYRSVIQNLLGTVLITEDLKGANE Bs
PLNKIRPRSVEAEVGV----------RAADLVEYDPRFEPAVKFALGDTVVVENMEEARP Py
663
LAKLLGHRYRIVTLEGDVVNPGGSMTGG
----YIGKVRMVTLEGELFEKSGAITGG
652
D
PfSmcH-CC60
PySmcCC3
BsSmcCC2
BsSmcCC1
PySmcHd-CC80
E
Cross-linked fraction (in Bs Smc)
0.0
0.2
0.4
0.6
0.8
10
20
30
40
50
60
Cα-Cα distance (Å) (in Py Smc rod model)

Figure S4

Figure S4. Reconstruction and evaluation of the Smc rod structure. Related to Figure 4.

(A) Structural overview (left panel) of *Py*SmcCC3. Regions displaying deviations from the canonical coiled coil architecture are shown in boxed insets (middle panels). Residues P434 and G435 result in the kinking of the N-terminal α–helix (top panel), while two residues lacking in the C-terminal helix cause a deformation of the α–helical pattern. Sequence alignments are shown for these selected regions (right panels).

(B) A single amino-acid insertion in the N-terminal *Pf* Smc coiled coil. Sequences were aligned taking into account the predicted coiled coil register.

(C) *Bs* Smc hinge domain residues corresponding to *Py* Smc residues. Numbers above and below the sequence lines denote *Bs* and *Py* Smc residues, respectively.

(D) Superimposition of the Smc rod model with individual crystal structures. Five structures were superimposed with the blue monomer of the Smc rod model shown in Figure 4B. The top and bottom ends of crystal structures were slightly adjusted manually to connect them into a straight molecule.

(E) Plot of Cα-Cα distance against cross-linking efficiency for the *Py* Smc rod model shown in Figure 4C. The data is available in Table S5.

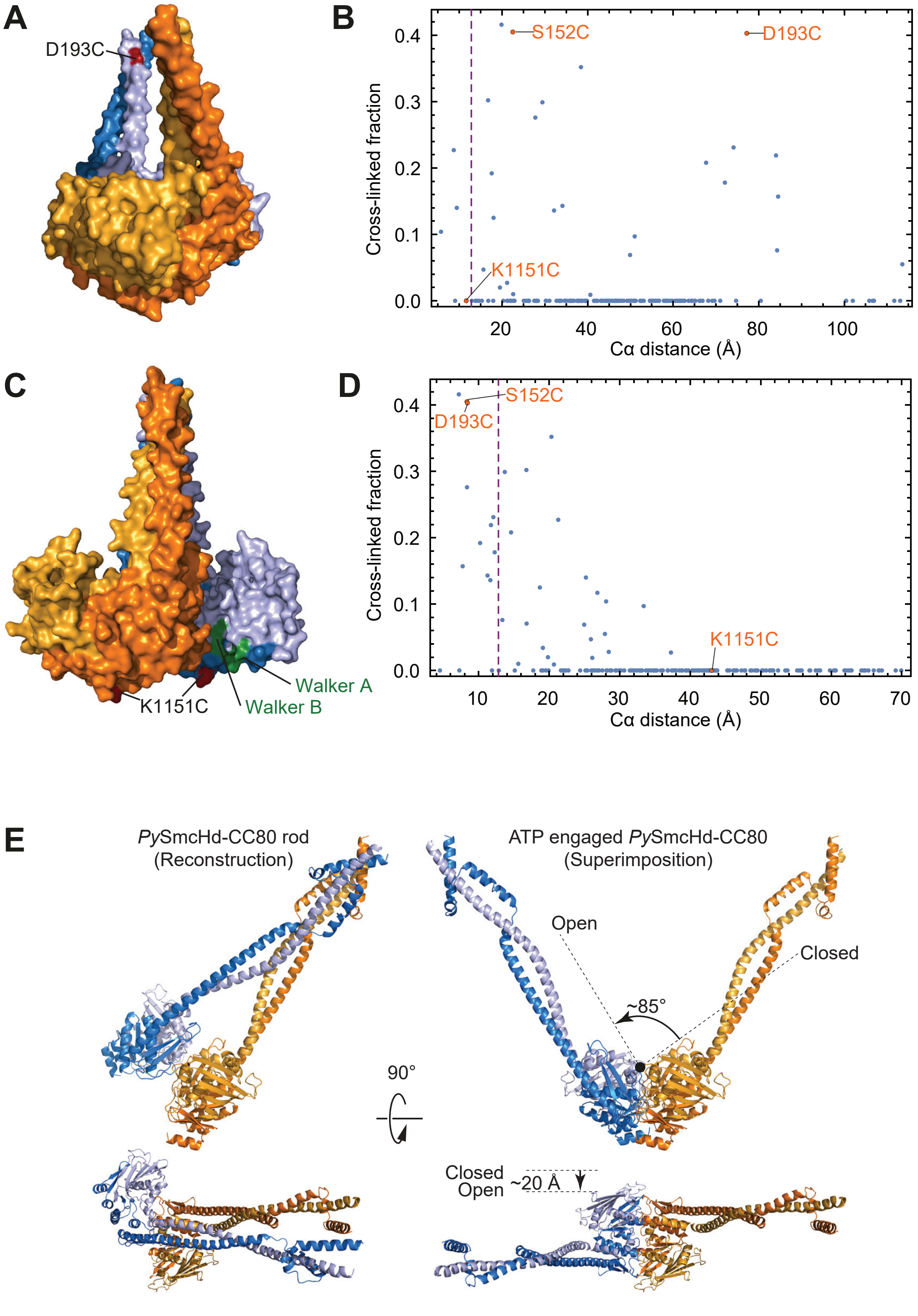
A
D193C
B
S152C
D193C
K1151C
Cross-linked fraction
0.0
0.1
0.2
0.3
0.4
20
40
60
80
100
Cα distance (Å)
C
K1151C
Walker A
Walker B
D
S152C
D193C
K1151C
Cross-linked fraction
10
20
30
40
50
60
70
Cα distance (Å)
E
PySmcHd-CC80 rod
(Reconstruction)
ATP engaged PySmcHd-CC80
(Superimposition)
Open
Closed
~85°
90°
Closed
Open
~20 Å

Figure S5

Figure S5. Structural comparison of rod-aligned and ATP-engaged Smc heads. Related to Figure 5.

(A-D) Characterization of engaged (A, B) and juxtaposed (C, D) Smc heads. Side views of models shown in Figure 5B and 5C (A, C, respectively). Plotting Cα-Cα distance distribution against cross-linking efficiencies shown in Figure 2A (B, D).

(E) Comparison of rod-aligned (left panels; taken from Figure 4B) and ATP-engaged *Py*SmcHd-CC80 (right panels; superimposed onto PDB: 1XEX) in side (top panels) and top views (bottom panels). The position and orientation of the monomer in orange colors in identical in the two top and the two bottom panels. The monomer in blue colors tilts by about 85° relative to the other monomer (top panel) and translates by about 20 Å.

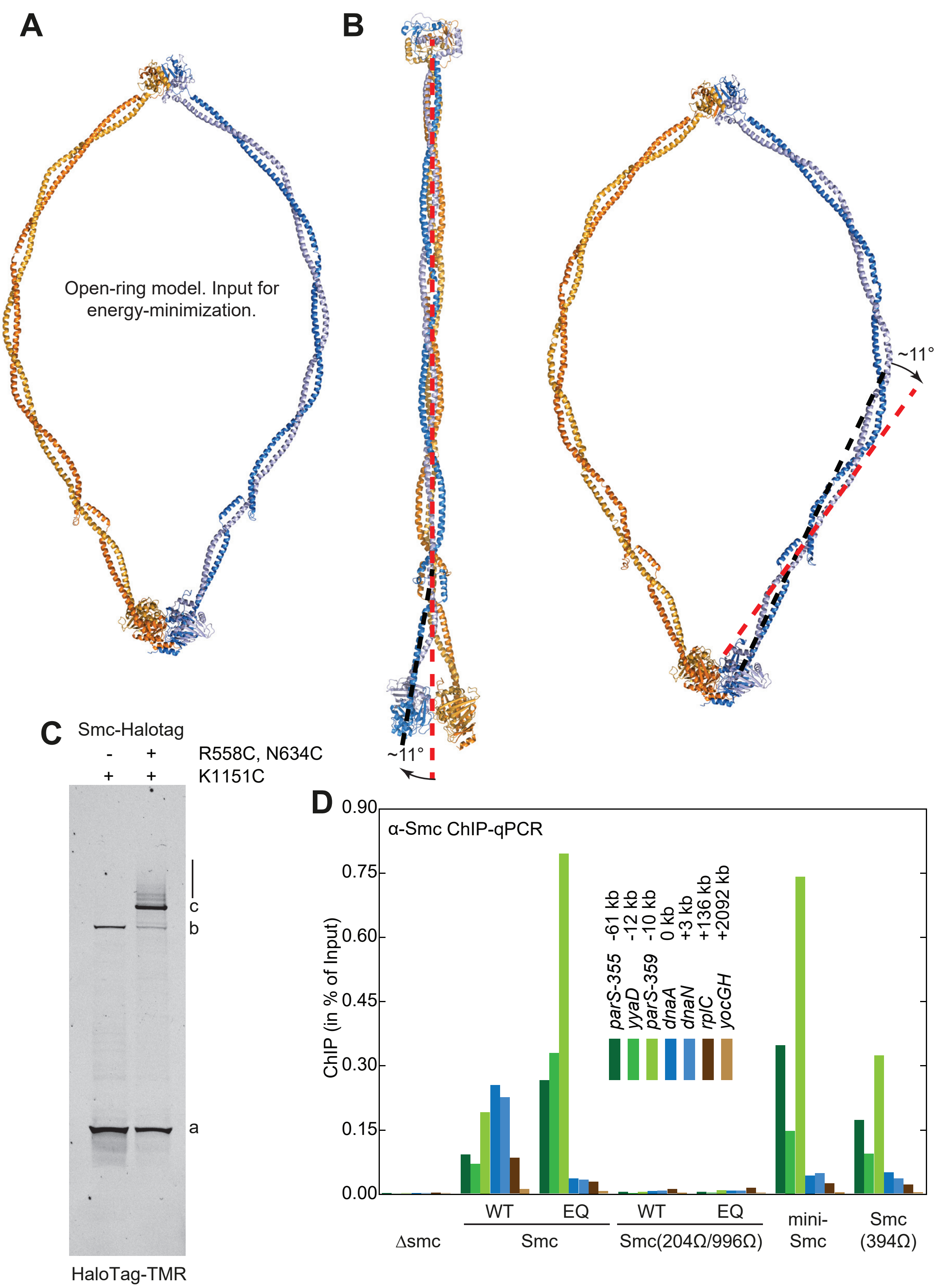
A
Open-ring model. Input for energy-minimization.
B
~11°
~11°
C
Smc-Halotag
- + R558C, N634C
+ + K1151C
c
b
a
HaloTag-TMR
D
α-Smc ChIP-qPCR
ChIP (in % of Input)
0.90
0.75
0.60
0.45
0.30
0.15
0.00
parS-355 -61 kb
yyaD -12 kb
parS-359 -10 kb
dnaA 0 kb
dnaN +3 kb
rplC +136 kb
yocGH +2092 kb
Δsmc
WT
EQ
Smc
WT
EQ
Smc(204Ω/996Ω)
mini-Smc
Smc (394Ω)

Figure S6

Figure S6. Model of an open Smc dimer. Related to Figure 6.

(A) Input model for energy-minimization constructed by bending of the coiled coil from the Smc hinge to the Smc joint.

(B) The Smc joint – as observed in the model of the Smc rod (left panel) – bends the coiled coil away from the symmetry axis possibly to support wide opening of the Smc ring upon head engagement (right panel – energy minimized model).

(C) Simultaneous engagement of the Smc hinge and ATP dimer Smc heads. Similar to the experiment shown in Figure 6B using Smc proteins with wild-type ATPase domain. Please note that the contrast is enhanced to display low-abundance species.

(D) Chromosome localization of Smc proteins with modified coiled coils. Similar to the experiment shown in Figure 6F using Smc proteins lacking a HaloTag and cysteine modifications.

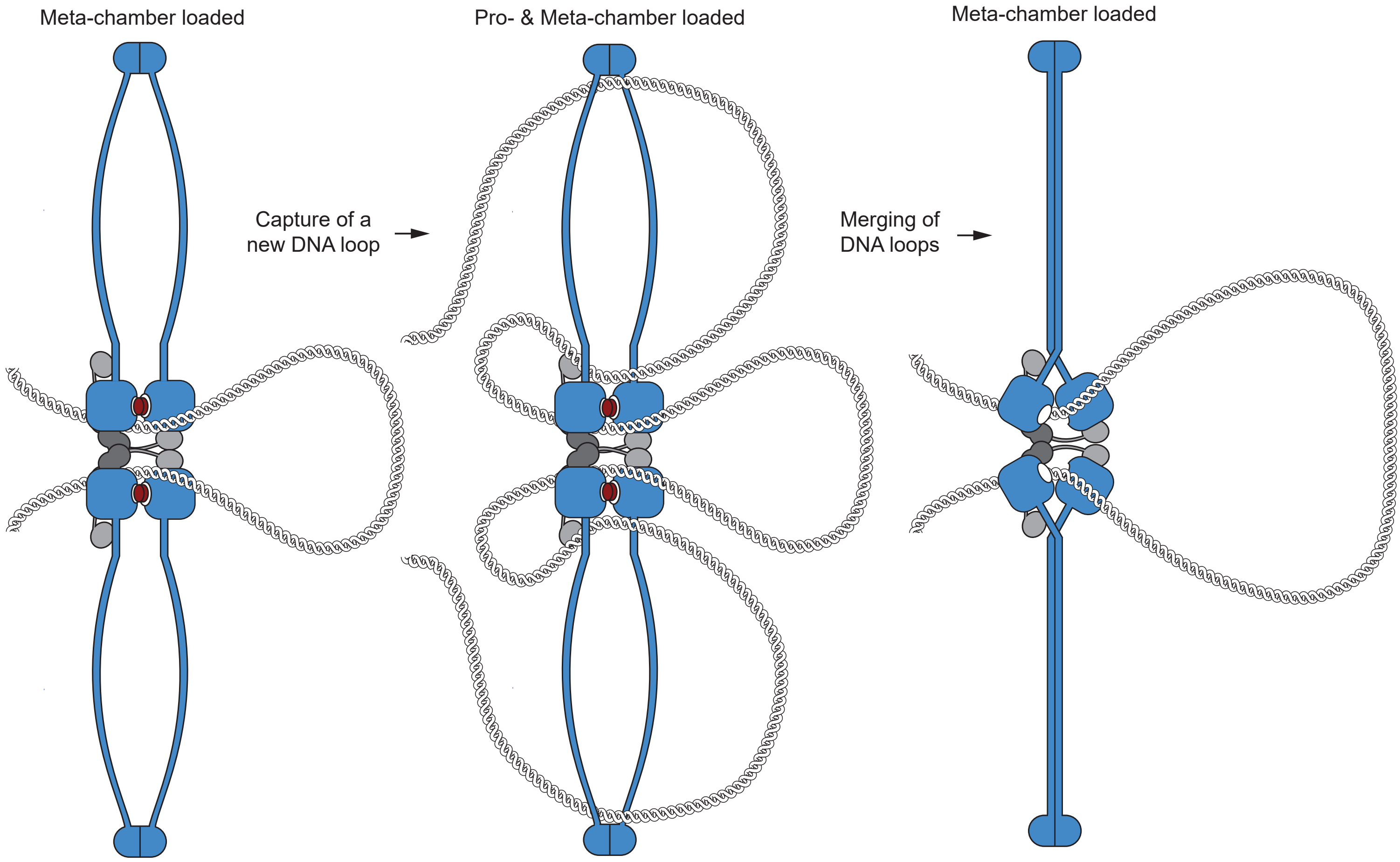
Meta-chamber loaded
Capture of a new DNA loop
Pro- & Meta-chamber loaded
Merging of DNA loops
Meta-chamber loaded

Figure S7

Figure S7. A tentative SMC handcuff model. Related to Figure 7.

As in Figure 7, involving a putative ‘handcuff’ Smc-ScpAB dimer. The two Smc-ScpAB complexes translocate in opposite orientation on the DNA, each being topologically loaded onto a single DNA double helix.

Table S1, related to Figure 2 and 3. Data collection and structure refinement statistics.

Data Collection	***Bs*SmcCC2 SeMet**	***Bs*SmcCC1 SeMet**	***Bs*SmcCC1 native**
X-ray source	SLS PXII XD10SA[a]	DESY BL P11[b]	DESY BL P11[b]
Space group	P1	$P2_12_12$	$P2_12_12$
a, b, c (Å)	44.30, 42.73, 174.60	64.88, 77.32, 83.98	64.83, 78.03, 83.86
α, β, γ (°)	89.97, 93.64, 89.97	90, 90, 90	90, 90, 90
Wavelength (Å)	0.9792	0.9793	0.9806
Resolution (Å)	87.12 - 2.892	50.0-2.38	50.0-1.8
R_{sym} (%)[d]	8.3 (61)	5.8 (84.4)	4.0 (76.0)
$I/\sigma(I)$	12.33 (2.58)	20.45 (2.03)	16.95 (1.75)
Completeness (%)	98.54 (96.20)	99.8 (99)	99.7 (99.2)
Redundancy	3.6 (3.6)	6.9 (6.9)	4.3 (4.2)
Refinement			
Resolution (Å)	50.00 - 3.295		50.00 - 1.898
No. of reflections	37323		65454
R_{work} / R_{free} (%)	27.27 / 29.73		18.27 / 21.35
bond lengths (Å) / angles (°)	0.003 / 0.84		0.011 / 1.035
Average B-values (Å^2)	109.6		
Favored	98.29		98.63
allowed	1.49		1.03
Disallowed	0.21		0.34

[a]Villigen, Switzerland, [b]Hamburg Germany

[d]The numbers in parentheses are the statistics from the highest resolution shell.

Table S1, related to Figure 3, 4 and 5. Continued.

Data Collection	**PySmcHd-CC80**	***Bs*SmcHd-CC30: ATPγS–ScpA[c]**	***Py*SmcCC3**
X-ray source	BL5C, PAL[a]	BL5A, PF[b]	BL5C, PAL[a]
Space group	C2	$P2_12_12_1$	$P2_1$
a, b, c (Å)	120.32, 50.84, 122.06	88.10, 104.78, 185.22	36.18, 53.71, 71.40
α, β, γ (°)	90, 118.09, 90	90, 90, 90	90, 90.83, 90
Wavelength (Å)	0.97941	1.0000	0.97941
Resolution (Å)	50.0-2.6	50.0-3.5	50.0-2.0
R_{sym} (%)[d]	7.9 (32.4)	5.6 (27.9)	5.4 (23.5)
$I/\sigma(I)$	13.7 (2.6)	12.3 (3.7)	23.8 (2.1)
Completeness (%)	92.8 (69.1)	92.6 (86.5)	94.4 (78.5)
Redundancy	4.2 (2.0)	5.4 (1.8)	4.3 (2.5)
Refinement			
Resolution (Å)	50.0-2.6	50.0-3.5	50.0-2.0
No. of reflections	18871	35074	17586
R_{work} / R_{free} (%)	23.1 / 28.6	25.2 / 30.1	22.8 / 24.7
bond lengths (Å) / angles (°)	0.006 / 0.878	0.009 / 1.256	0.006 / 0.975
Average B-values (Å^2)	69.6	57.51	49.6
Most favored/Favored	89.6 / 9.0	87.6 / 10.6	97.7 / 2.3
Generously allowed	1.1	1.3	0.0
Disallowed	0.4	0.4	0.0

[a]Pohang Accelerator Laboratory, [b]Photon Factory

[d]The numbers in parentheses are the statistics from the highest resolution shell.

Table S2, related to Figures 2, 5 and 6. Strain usage.

Figure	Strains
5D	BSG2264, BSG2300, BSG1457, BSG2588, BSG2608, BSG1607, BSG2589, BSG2606, BSG1600, BSG2265, BSG2313, BSG1488, BSG2314, BSG2315, BSG1598
6B	BSG1488, BSG1640, BSG1666
6C	BSG1457, BSG1007, BSG2694
6D	BSG1457, BSG1488, BSG2694, BSG2693, BSG2408, BSG2135
6E	BSG1921, BSG1457, BSG1922, BSG1488, BSG2680, BSG2694, BSG2692, BSG2693
6F	BSG1007, BSG1457, BSG1488, BSG2694, BSG2693, BSG2408, BSG2135
S2C	BSG2326, BSG2324, BSG2382, BSG2381, BSG2270, BSG2322, BSG2063, BSG2320, BSG2413, BSG2299, BSG2379, BSG2378, BSG2267, BSG2377, BSG2376, BSG2375, BSG2300, BSG2264, BSG1921, BSG2383, BSG2385, BSG2386
S2D	BSG1360, BSG1007, BSG1008, BSG1921, BSG2299, BSG2379, BSG2378, BSG2267, BSG2377, BSG2376, BSG2375, BSG2300, BSG2264, BSG2383, BSG2385, BSG2386
S6C	BSG1638, BSG2638
S6D	BSG1007, BSG1002, BSG1008, BSG2648, BSG2688, BSG2091, BSG2018

Table S3, related to STAR methods. Primers used for qPCR

**Locus**	**Primer 1**	**Primer 2**
parS-355	taattcatcatcgcgctcaa	aatgccgattacgagtttgc
yyaD	cttgcgatttttgcttctcc	acatcaccatacgtggacga
parS-359	aaaaagtgattgcggagcag	agaaccgcatctttcacagg
dnaA	gatcaatcggggaaagtgtg	gtagggcctgtggatttgtg
dnaN	gaattccttcaggccattga	gatttctggcgaattggaag
rplC	ttgacgacaagcgtgaaaag	ttcatacgcatccatttcca
yocGH	tccatatcctcgctcctacg	attctgctgatgtgcaatgg
